# Ultrasound‐Activated Ce(IV)‐Integrated Biomimetic Nanoplatform for Oxygenation‐Reinforced Gas‐Sonodynamic Ferroptosis and cGAS–STING‐Driven Antitumor Immunity

**DOI:** 10.1002/advs.78187

**Published:** 2026-10-31

**Authors:** Mengli Ma, Yingying Wu, Yunjie Wang, Yang Li, Yijia Zhang, Zhibo Zhang, Jinzhi Peng, Xiangyu Wu, Yi Luo, Weijing Zhang, Xin Ye, Weisong Lai, Shengyu Wang, Dan Du, Wengang Li, Nan Zheng, Fanghong Luo, Ting Wu

**Affiliations:** ^1^ Cancer Research Center School of Medicine Xiamen University Xiamen People's Republic of China; ^2^ Department of Basic Medicine School of Medicine Xiamen University Xiamen People's Republic of China; ^3^ State Key Laboratory of Structural Chemistry CAS Key Laboratory of Design and Assembly of Functional Nanostructures Fujian Institute of Research on the Structure of Matter Chinese Academy of Sciences Fuzhou People's Republic of China; ^4^ School of Basic Medicine Fujian Medical University Fuzhou Fujian People's Republic of China; ^5^ Department of Hepatobiliary Surgery School of Medicine Xiang'an Hospital of Xiamen University Xiamen University Xiamen People's Republic of China; ^6^ School of Chemical Engineering Dalian University of Technology Dalian Liaoning People's Republic of China

**Keywords:** antitumor immunity, biomimetic nanoplatforms, carbon monoxide therapy, cGAS–STING, ferroptosis, sonodynamic therapy

## Abstract

Immunologically “cold” tumors remain poorly responsive to immunotherapy because hypoxia‐limited oxidative therapy, insufficient immunogenic tumor‐cell death, and dysfunctional T‐cell responses restrict the transition from local tumor destruction to systemic antitumor immunity. Here, an ultrasound‐responsive Ce(IV)‐integrated biomimetic nanoplatform, TcEM@Ce‐P4CO@M, is developed to address these barriers. Ce^4+^/Ce^3+^ redox cycling promotes H_2_O_2_ decomposition and O_2_ generation, sustaining ultrasound‐triggered reactive oxygen species production under hypoxic conditions. Concurrently, P4CO‐derived carbon monoxide contributes to mitochondrial oxidative stress and lipid peroxidation. Endogenous iron‐homeostasis remodeling and impairment of the SLC7A11/glutathione (GSH)/glutathione peroxidase 4 (GPX4) antioxidant defense further favor ferroptosis‐associated tumor‐cell injury. Mitochondrial dysfunction and DNA damage are accompanied by enhanced stimulator of interferon genes (STING)‐associated signaling, while co‐delivered MSA‐2 further reinforces STING‐mediated innate immune activation, collectively contributing to an immunogenic environment that supports dendritic‐cell maturation. The engineered tumor‐derived membrane promotes tumor‐associated delivery and provides CD86‐mediated costimulation and anti‐TIM‐3‐mediated checkpoint modulation. In 4T1 tumor models, this coordinated cascade suppresses primary tumors, inhibits non‐irradiated distant tumors, reduces experimental pulmonary metastatic burden, and remodels the tumor immune microenvironment. Collectively, TcEM@Ce‐P4CO@M converts hypoxia‐limited local sonodynamic injury into ferroptosis‐linked innate‐to‐adaptive systemic antitumor immunity.

## Graphical abstract

A Ce(IV)‐integrated biomimetic nanoplatform coordinates tumor oxygenation, ultrasound‐triggered carbon monoxide release, sonodynamic therapy, ferroptosis amplification, and cyclic GMP–AMP synthase (cGAS)–stimulator of interferon genes (STING) immune activation. Engineered membrane camouflage further provides CD86‐mediated costimulation and anti‐T‐cell immunoglobulin and mucin‐domain containing‐3 (TIM‐3) checkpoint modulation, converting local oxidative injury into systemic antitumor immunity and resulting in primary tumor inhibition, distant tumor suppression, and reduced lung metastasis.

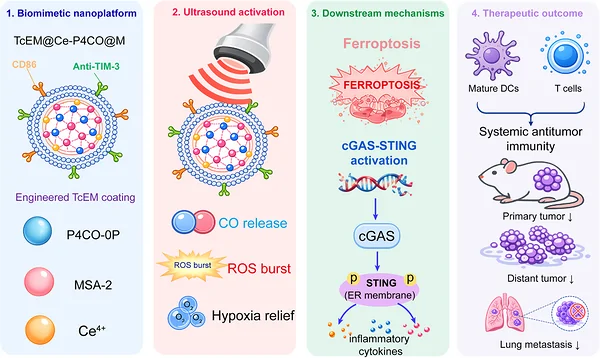

## 1 Introduction

Immune checkpoint blockade (ICB) has transformed cancer therapy, but its efficacy remains limited in immunologically “cold” tumors, where severe hypoxia, insufficient immunogenic tumor‐cell injury, and dysfunctional T‐cell responses collectively restrict durable antitumor immunity [1, 2]. These barriers are mechanistically interconnected: hypoxia compromises oxygen‐dependent tumor damage, insufficient immunogenic injury limits the generation of innate immune‐activating signals, and persistent inhibitory signaling further weakens effector T‐cell function. Therefore, effective treatment of cold tumors requires not only enhanced local tumor destruction, but also efficient conversion of this injury into innate immune activation followed by reinforcement of adaptive antitumor responses.

Sonodynamic therapy (SDT) provides a noninvasive approach for generating reactive oxygen species (ROS), yet its efficacy is strongly constrained by tumor hypoxia [3]. Carbon monoxide (CO) can perturb mitochondrial respiration and redox homeostasis, thereby sensitizing tumor cells to oxidative stress, whereas ferroptosis is characterized by iron‐dependent lipid peroxidation together with collapse of antioxidant defense systems, particularly the SLC7A11/glutathione (GSH)/glutathione peroxidase 4 (GPX4) axis [4]. Cerium‐based catalytic systems offer a potential means to overcome the upstream oxygen limitation because reversible Ce^4+^/Ce^3+^ cycling can promote H_2_O_2_ decomposition and local O_2_ generation, thereby restoring oxygen‐dependent sonodynamic activity [5, 6]. Beyond local tumor killing, oxidative and ferroptosis‐associated mitochondrial and DNA injury can enhance damage‐associated molecular pattern (DAMP) signaling and cytosolic DNA‐associated innate immune sensing, including activation of the cyclic GMP–AMP synthase (cGAS)–stimulator of interferon genes (STING) pathway and downstream dendritic‐cell maturation [7, 8]. Pharmacological STING agonists such as MSA‐2 may further reinforce this conversion from local tumor injury to innate immune activation [9]. However, effective antitumor immunity also requires subsequent recovery of T‐cell function, suggesting that upstream microenvironmental modulation, immunogenic tumor‐cell injury, innate immune sensing, and adaptive immune regulation should ideally be coordinated rather than addressed as isolated therapeutic events.

Herein, we developed an ultrasound‐responsive Ce(IV)‐integrated biomimetic nanoplatform, TcEM@Ce‐P4CO@M, to address these interconnected therapeutic barriers. The coordination‐assembled core incorporates the sonosensitizer/CO‐donor precursor P4CO, the STING agonist MSA‐2, and Ce species, and is further coated with engineered tumor‐derived membranes displaying CD86 and anti–T‐cell immunoglobulin and mucin‐domain containing‐3 (TIM‐3). The tumor‐derived membrane promotes homologous tumor‐associated delivery, while Ce‐mediated oxygenation sustains sonodynamic ROS generation under hypoxic conditions. Upon ultrasound irradiation, sonodynamic ROS and CO jointly promote mitochondrial oxidative stress and ferroptosis‐associated tumor‐cell injury, accompanied by endogenous iron‐homeostasis remodeling, DAMP exposure/release, and DNA‐damage‐associated cGAS–STING activation that is further reinforced by MSA‐2. Meanwhile, CD86‐mediated costimulation and anti‐TIM‐3‐mediated checkpoint modulation support subsequent T‐cell activation. As illustrated in Scheme 1, this spatiotemporally coordinated therapeutic cascade is designed to convert hypoxia‐limited local sonodynamic injury into ferroptosis‐linked innate and adaptive antitumor immune activation, thereby suppressing primary tumors, inhibiting distant tumors, and restraining lung metastasis.

**SCHEME 1:**
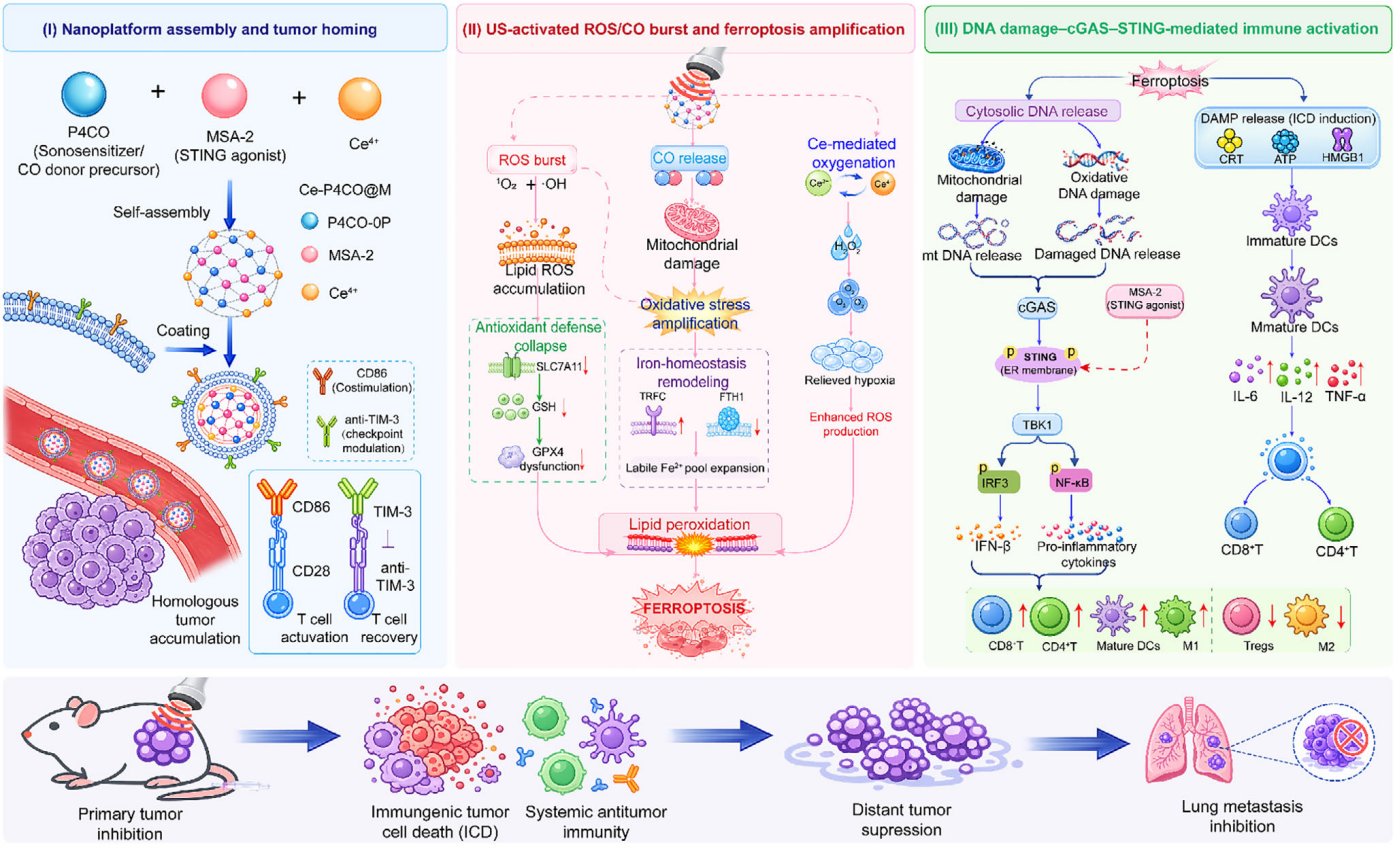
Schematic illustration of TcEM@Ce‐P4CO@M‐mediated oxygenation‐reinforced gas‐sonodynamic ferroptosis and cGAS–STING‐driven antitumor immunity.

## 2 Results

### 2.1 Construction and Physicochemical Characterization of TcEM@Ce‐P4CO@M

TcEM@Ce‐P4CO@M was constructed through a stepwise coordination‐assembly and membrane‐coating strategy (Figure 1A). The previously reported ultrasound‐responsive CO‐releasing porphyrin P4CO‐0P (hereafter referred to as P4CO) [10] and the STING agonist MSA‐2 were incorporated into a Ce(IV)‐mediated coordination core to form Ce‐P4CO@M, which was subsequently coated with the dual‐engineered tumor‐derived membrane (TcEM). Dynamic light scattering (DLS) analysis showed a shift in hydrodynamic size after TcEM coating, while TEM imaging revealed well‐dispersed spherical nanoparticles and a discernible membrane‐associated peripheral structure in the coated formulation (Figure 1B,C), together supporting successful formation of the biomimetic nanoplatform.

**FIGURE 1:**
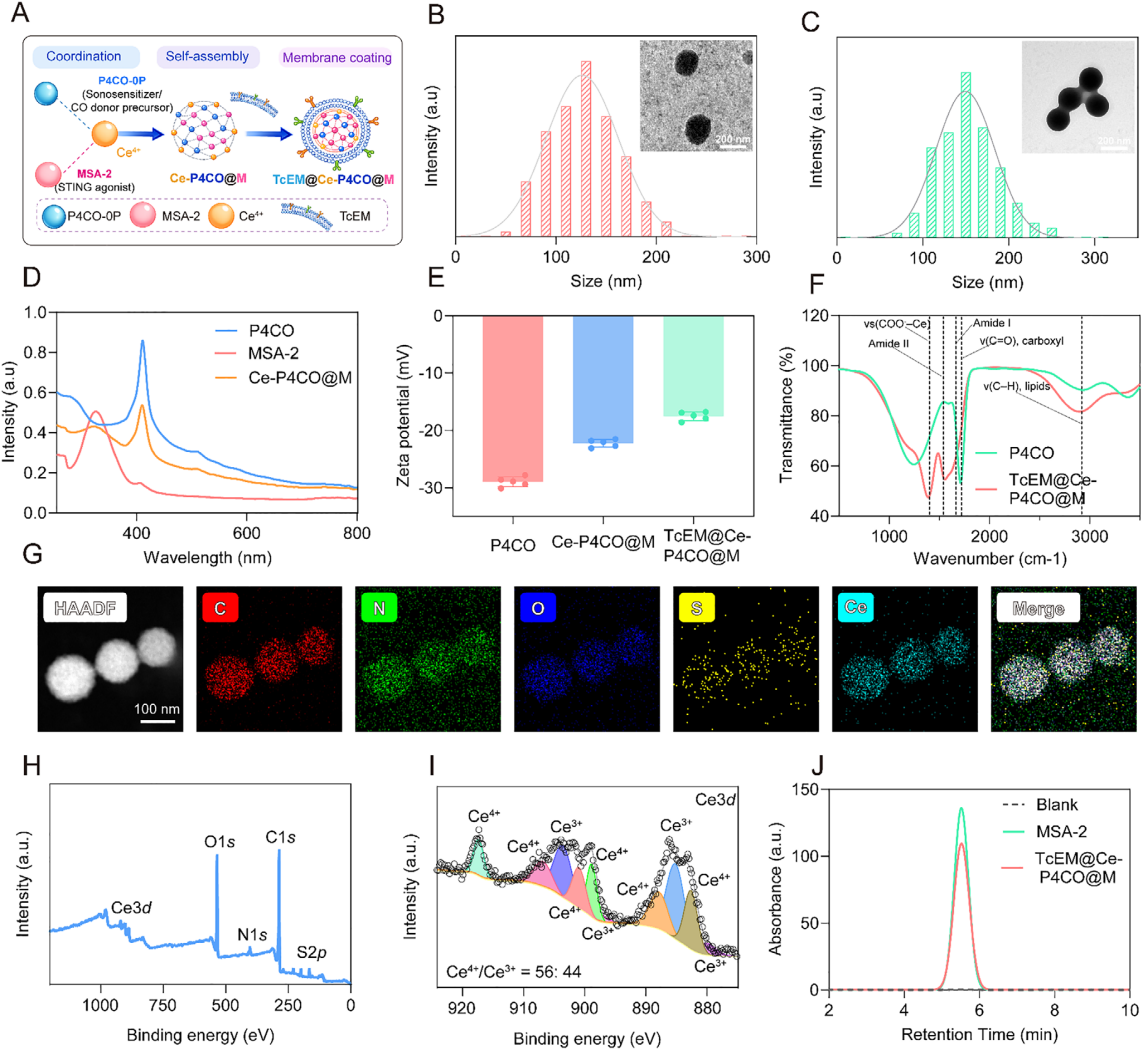
Fabrication and physicochemical characterization of TcEM@Ce‐P4CO@M. (A) Schematic illustration of the stepwise fabrication of TcEM@Ce‐P4CO@M through Ce(IV)‐mediated coordination assembly of P4CO and MSA‐2 followed by coating with the engineered tumor‐derived membrane TcEM. (B, C) DLS hydrodynamic size distributions of Ce‐P4CO@M and TcEM@Ce‐P4CO@M, respectively; corresponding TEM images are shown as insets. (D) UV–vis absorption spectra of P4CO, MSA‐2, and Ce‐P4CO@M. (E) Zeta potentials of P4CO, Ce‐P4CO@M, and TcEM@Ce‐P4CO@M, showing stepwise surface‐charge changes during coordination assembly and membrane coating. Data in (E) are presented as mean ± SD (*n* = 5 independent measurements). (F) FT–IR spectra of P4CO and TcEM@Ce‐P4CO@M showing characteristic spectral changes after assembly and membrane coating. (G) HAADF‐STEM image and corresponding EDS elemental mappings of C, N, O, S, and Ce in TcEM@Ce‐P4CO@M. Scale bar: 100 nm. (H) XPS survey spectrum of TcEM@Ce‐P4CO@M showing the major constituent elements. (I) High‐resolution Ce 3d XPS spectrum showing coexistence of Ce4+ and Ce3+ species, with an approximate Ce^4+^/Ce^3+^ ratio of 56:44. (J) HPLC chromatograms of the blank control, free MSA‐2, and the TcEM@Ce‐P4CO@M extract. The chromatographic peak of the nanoparticle extract at a retention time corresponding to free MSA‐2 supports successful incorporation of MSA‐2.

The stepwise assembly was further examined by spectroscopic and surface‐charge analyses. Ce‐P4CO@M retained the characteristic Ultraviolet–visible (UV–vis) absorption features associated with P4CO and MSA‐2 (Figure 1D), while the progressive change in zeta potential from P4CO to Ce‐P4CO@M and subsequently to TcEM@Ce‐P4CO@M was consistent with coordination assembly followed by membrane coating (Figure 1E). Fourier‐transform infrared (FT–IR) spectroscopy further showed P4CO‐associated spectral features together with membrane‐derived protein/lipid‐related signals in TcEM@Ce‐P4CO@M (Figure 1F). In addition, sodium dodecyl sulfate–polyacrylamide gel electrophoresis (SDS‐PAGE) demonstrated that the membrane‐coated nanoparticles retained the major protein profile of TcEM (Figure S1), further supporting retention of membrane‐associated proteins after coating.

We next examined the incorporation and valence state of Ce, because the Ce^4+^/Ce^3+^ redox couple provides the chemical basis for the subsequent catalytic oxygenation function of the platform. High‐angle annular dark‐field scanning transmission electron microscopy/energy‐dispersive X‐ray spectroscopy (HAADF‐STEM/EDS) mapping showed spatially distributed Ce signals together with C, N, O, and S within the nanoparticles (Figure 1G). Consistently, the X‐ray photoelectron spectroscopy (XPS) survey spectrum confirmed the presence of Ce and the major constituent elements (Figure 1H). High‐resolution Ce 3d analysis revealed the coexistence of Ce^4+^ and Ce^3+^ species, with an approximate Ce^4+^/Ce^3+^ ratio of 56:44 (Figure 1I), confirming the mixed‐valence Ce state that provides the chemical basis for subsequent redox cycling. Additional high‐resolution O 1s, S 2p, and N 1s spectra are provided in Figure S2A–C as complementary chemical‐state characterization.

The successful incorporation of MSA‐2 was further verified by high‐performance liquid chromatography (HPLC). The extract from TcEM@Ce‐P4CO@M exhibited a chromatographic peak at a retention time consistent with that of free MSA‐2, whereas no corresponding peak was detected in the blank control (Figure 1J). The resulting formulation also maintained relatively stable hydrodynamic size, zeta potential, and polydispersity index (PDI) during incubation in phosphate‐buffered saline (PBS), Dulbecco's modified Eagle medium (DMEM), and 10% fetal bovine serum (FBS)‐containing DMEM (Figure S3A–C), indicating favorable colloidal stability under the tested media conditions. Moreover, MSA‐2 release was enhanced under acidic and GSH‐containing conditions compared with physiological pH conditions (Figure S4), indicating microenvironment‐responsive release behavior.

Collectively, these results confirm the successful construction of a membrane‐coated, mixed‐valence Ce‐containing nanoplatform integrating the ultrasound‐responsive P4CO module and MSA‐2, thereby establishing the physicochemical basis for the subsequent oxygenation, gas‐sonodynamic, and immunomodulatory studies.

### 2.2 Ultrasound‐Triggered CO Release and ROS Generation by TcEM@Ce‐P4CO@M

After confirming the successful construction of TcEM@Ce‐P4CO@M, we next investigated whether the P4CO module retained its ultrasound‐responsive gas‐sonodynamic properties after incorporation into the nanoplatform. The previously reported P4CO undergoes ultrasound‐triggered structural transformation accompanied by CO release, as schematically illustrated in Figure 2A. The corresponding time‐dependent spectral evolution of P4CO under different acoustic power densities is provided in Figure S5. TcEM@Ce‐P4CO@M exhibited progressively increased CO release during ultrasound (US) irradiation, and the release process could be repeatedly regulated by alternating ultrasound on/off cycles (Figure 2B). Under continuous irradiation, cumulative CO release increased with increasing acoustic power density (Figure 2C), supporting acoustic‐power‐dependent control of CO release. The released CO was further verified using a myoglobin (Mb)‐based spectroscopic assay [11], which showed time‐dependent spectral changes consistent with CO binding to Mb after ultrasound activation (Figure 2D). Collectively, these results demonstrate that incorporation of P4CO into TcEM@Ce‐P4CO@M preserves its controllable ultrasound‐triggered CO‐releasing behavior.

**FIGURE 2:**
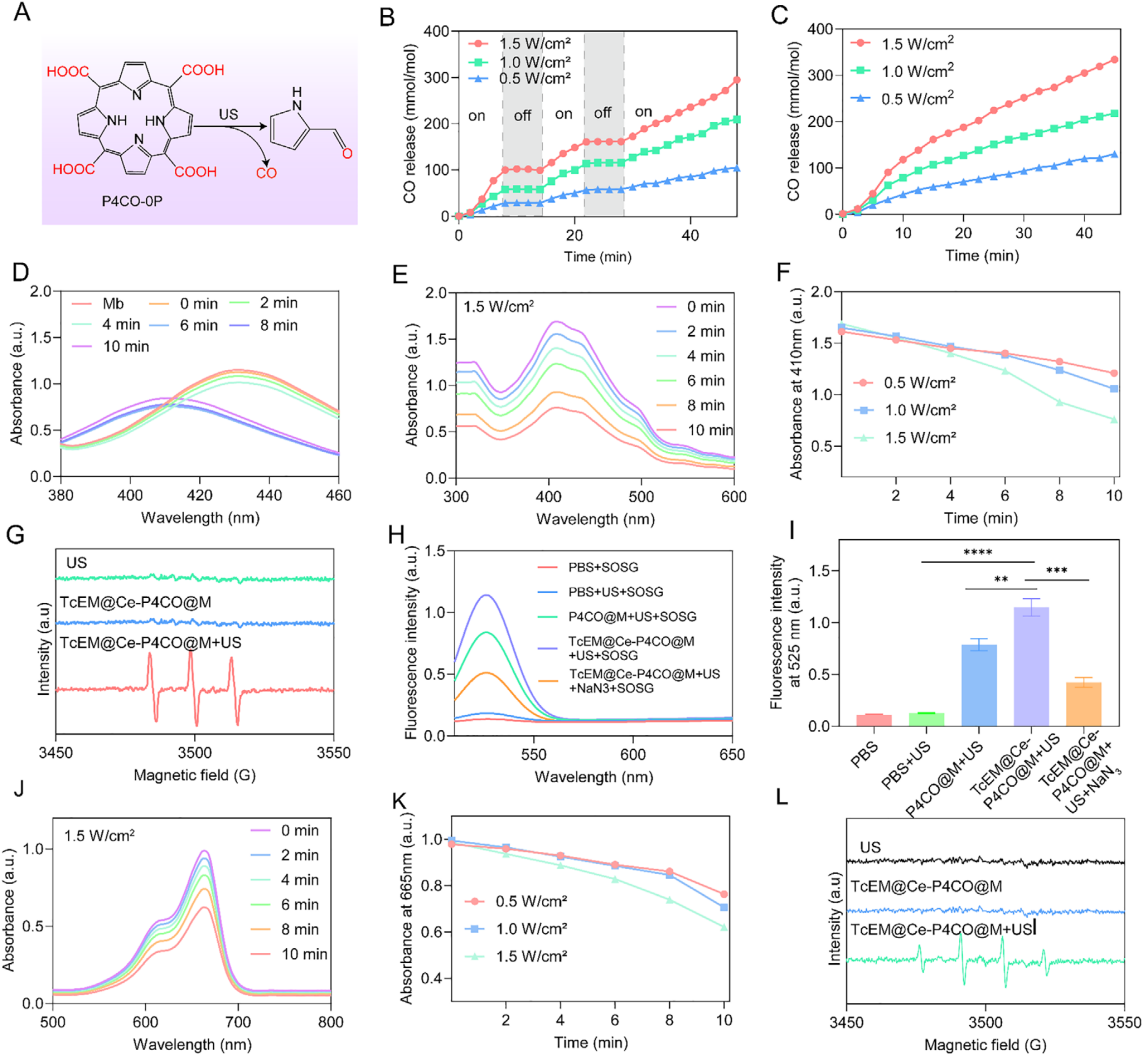
Ultrasound‐triggered CO release and ROS generation by TcEM@Ce‐P4CO@M. (A) Schematic illustration of the ultrasound‐triggered structural transformation of P4CO‐0P accompanied by CO release. (B) Ultrasound on/off‐controlled CO release from TcEM@Ce‐P4CO@M at acoustic power densities of 0.5, 1.0, and 1.5 W cm^−2^. Shaded regions indicate ultrasound‐off periods. (C) Cumulative CO release from TcEM@Ce‐P4CO@M during continuous ultrasound irradiation at the indicated acoustic power densities. (D) Time‐dependent UV–vis absorption spectra of myoglobin (Mb) during ultrasound‐triggered CO release, showing spectral changes associated with CO binding to Mb. (E) Time‐dependent DPBF absorption spectra during ultrasound irradiation at 1.5 W cm^−2^. (F) Corresponding changes in DPBF absorbance at 410 nm under ultrasound irradiation at 0.5, 1.0, and 1.5 W cm^−2^. (G) ESR spectra obtained under the indicated treatment conditions, showing ultrasound‐triggered spin‐adduct formation. (H) SOSG fluorescence spectra under the indicated treatment conditions. (I) Quantification of SOSG fluorescence intensity at 525 nm. The reduction in SOSG fluorescence after NaN_3_ treatment supports the involvement of singlet oxygen (^1^O_2_). (J) Time‐dependent methylene blue (MB) absorption spectra during ultrasound irradiation at 1.5 W cm^−2^. (K) Corresponding changes in MB absorbance at 665 nm under ultrasound irradiation at 0.5, 1.0, and 1.5 W cm^−2^. (L) ESR spectra obtained under the indicated treatment conditions using an additional spin‐trapping system to further verify ultrasound‐triggered ROS generation. Data in (I) are presented as mean ± SD (*n* = 3 independent measurements). Statistical significance was determined by one‐way ANOVA followed by Tukey's multiple‐comparisons test. Brackets indicate the specific group comparisons shown in the figure. ^**^ *p* < 0.01, ^***^ *p* < 0.001, ^****^ *p* < 0.0001.

We next evaluated the ultrasound‐triggered ROS‐generating capability of the nanoplatform using complementary probe‐based assays and electron spin resonance (ESR) spectroscopy. 1,3‐Diphenylisobenzofuran (DPBF) absorbance progressively decreased during ultrasound irradiation, with a more pronounced reduction at 410 nm at higher acoustic power densities (Figure 2E,F). The corresponding complete spectra obtained at 0.5 and 1.0 W cm^−2^ are provided in Figure S6, further confirming the acoustic power‐dependent oxidative response.

To further examine the involvement of singlet oxygen (^1^O_2_), singlet oxygen sensor green (SOSG) was used together with the ^1^O_2_ quencher NaN_3_ [12]. Only low SOSG fluorescence was observed in the PBS and PBS + US controls, whereas P4CO@M + US produced a pronounced increase in fluorescence intensity. A further increase was observed for TcEM@Ce‐P4CO@M + US. Importantly, addition of NaN_3_ substantially attenuated the SOSG signal generated by TcEM@Ce‐P4CO@M + US (Figure 2H,I), supporting the involvement of ^1^O_2_ in the ultrasound‐triggered oxidative response.

Methylene blue (MB) bleaching provided additional evidence of ultrasound‐dependent oxidative activity. MB absorbance progressively decreased with irradiation time, and the reduction became more pronounced at higher acoustic power densities (Figure 2J,K). The corresponding complete spectra at lower acoustic powers are provided in Figure S7. ESR spin‐trapping measurements further showed distinct spin‐adduct signals after ultrasound activation of TcEM@Ce‐P4CO@M [13], whereas ultrasound alone or TcEM@Ce‐P4CO@M without irradiation produced negligible signals (Figure 2G,L), providing orthogonal evidence for ultrasound‐triggered ROS generation.

Complementary 5,5′‐dithiobis(2‐nitrobenzoic acid) (DTNB)‐based measurements further showed time‐ and acoustic power‐dependent changes consistent with enhanced thiol oxidation following ultrasound activation (Figure S9), while the corresponding reaction schemes of the DPBF‐, MB‐, and DTNB‐based assays are provided in Figure S8. Collectively, these physicochemical measurements establish TcEM@Ce‐P4CO@M as an ultrasound‐activatable platform capable of controllable CO release and ultrasound‐triggered ROS generation. Because oxygen availability is a major determinant of sonodynamic ROS production, we next investigated whether Ce‐mediated oxygenation could preserve sonodynamic oxidative activity under hypoxic conditions.

### 2.3 Ce‐Mediated Oxygenation Attenuates Hypoxia‐Associated Responses and Preserves Sonodynamic ROS Generation

Although the preceding results established that TcEM@Ce‐P4CO@M retains ultrasound‐triggered CO release and ROS‐generating activity, oxygen‐dependent sonodynamic reactions are strongly compromised under hypoxic conditions. Cerium‐based catalytic systems offer a potential strategy to overcome this limitation because Ce^4+^/Ce^3+^ redox cycling can facilitate H_2_O_2_ decomposition and O_2_ generation [6]. We therefore investigated whether Ce incorporation could provide functional oxygenation and preserve sonodynamic oxidative activity under hypoxia (Figure 3A).

**FIGURE 3:**
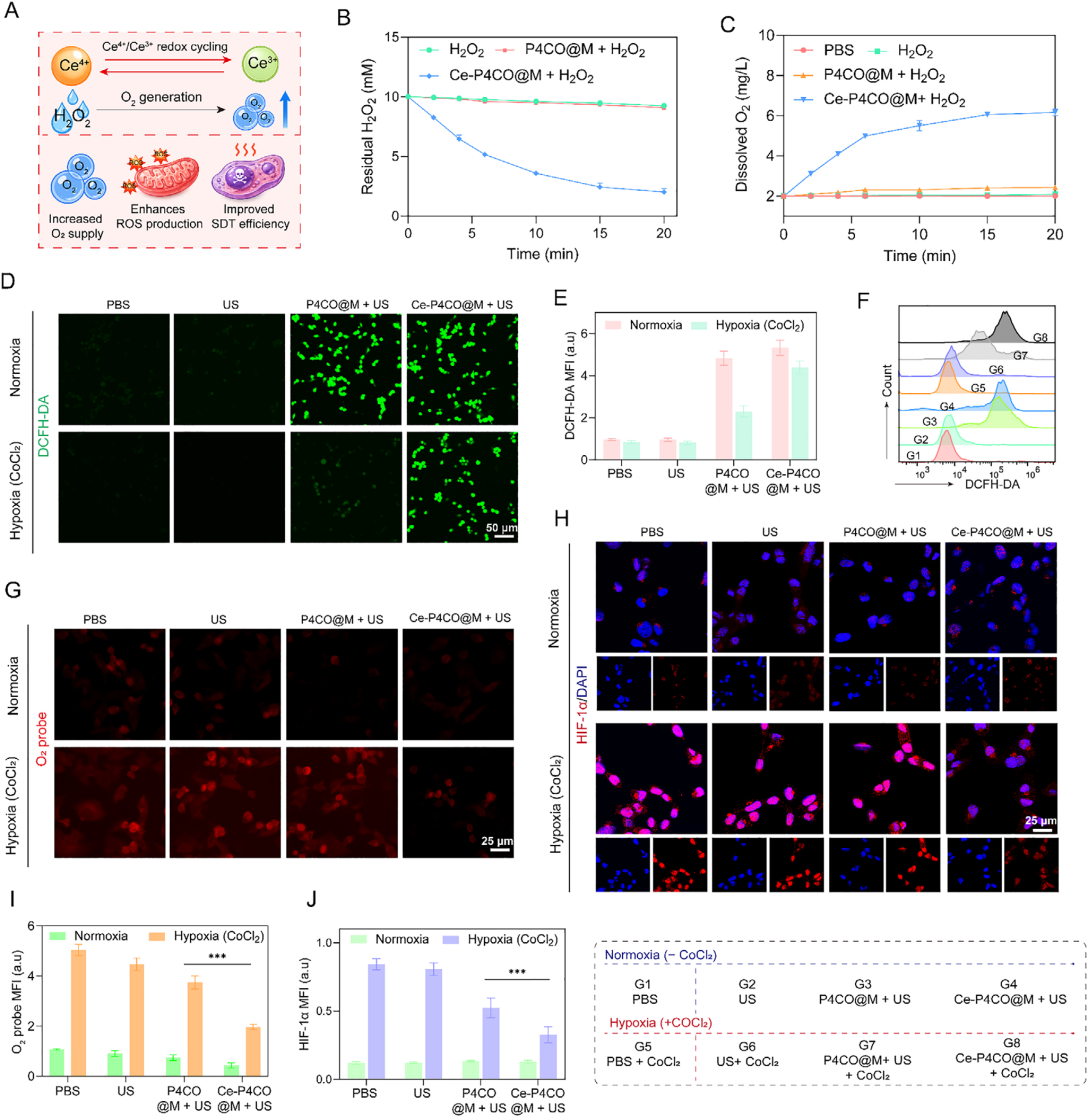
Ce‐mediated oxygenation attenuates hypoxia‐associated responses and preserves sonodynamic ROS generation. (A) Schematic illustration of Ce^4+^/Ce^3+^ redox cycling, H_2_O_2_ conversion, and O_2_ generation, and the proposed enhancement of oxygen‐dependent sonodynamic activity. (B) Time‐dependent residual H_2_O_2_ concentrations in H_2_O_2_ alone, P4CO@M + H_2_O_2_, and Ce‐P4CO@M + H_2_O_2_ groups. (C) Time‐dependent dissolved O_2_ concentrations in PBS, H_2_O_2_, P4CO@M + H_2_O_2_, and Ce‐P4CO@M + H_2_O_2_ groups. (D) Representative DCFH‐DA fluorescence images after the indicated treatments under normoxic and CoCl_2_‐induced hypoxia‐mimetic conditions. Scale bar: 50 µm. (E) Quantification of DCFH‐DA mean fluorescence intensity (MFI) corresponding to (D). (F) Flow cytometric analysis of DCFH‐DA fluorescence across the corresponding normoxic and CoCl_2_‐treated groups. (G) Representative hypoxia‐sensitive fluorescence images under normoxic and CoCl_2_‐induced hypoxia‐mimetic conditions. Scale bar: 25 µm. (H) Representative HIF‐1α immunofluorescence images under the indicated conditions. Nuclei were counterstained with DAPI. Scale bar: 25 µm. (I) Quantification of the hypoxia‐associated fluorescence signal corresponding to (G). (J) Quantification of HIF‐1α fluorescence intensity corresponding to (H). Groups: G1, PBS; G2, US; G3, P4CO@M + US; G4, Ce‐P4CO@M + US; G5, PBS + CoCl_2_; G6, US + CoCl_2_; G7, P4CO@M + US + CoCl_2_; G8, Ce‐P4CO@M + US + CoCl_2_. Quantitative data are presented as mean ± SD (*n* = 3 independent experiments). Statistical significance for the quantitative fluorescence analyses in (E), (I), and (J) was determined by two‐way ANOVA followed by Tukey's multiple‐comparisons test. Brackets indicate the specific group comparisons shown in the figure. ^***^ *p* < 0.001.

We first evaluated H_2_O_2_ consumption and dissolved O_2_ generation in cell‐free systems. H_2_O_2_ alone remained largely unchanged during the observation period, whereas P4CO@M produced only a limited decrease in residual H_2_O_2_. In contrast, Ce‐P4CO@M markedly accelerated H_2_O_2_ depletion over time (Figure 3B). Consistently, dissolved O_2_ concentrations remained nearly unchanged in the PBS and H_2_O_2_ controls and increased only slightly in the P4CO@M + H_2_O_2_ group, whereas Ce‐P4CO@M + H_2_O_2_ induced a pronounced time‐dependent increase in dissolved O_2_ (Figure 3C). These results indicate that the major oxygen‐generating activity of the platform is associated with the Ce‐containing formulation rather than the P4CO@M component alone.

We next examined whether Ce‐mediated oxygenation could preserve intracellular ROS generation under CoCl_2_‐induced hypoxia‐mimetic conditions. Under normoxic conditions, both P4CO@M + US and Ce‐P4CO@M + US induced substantial 2′,7′‐dichlorodihydrofluorescein diacetate (DCFH‐DA)fluorescence, whereas PBS and US alone produced only weak signals (Figure 3D,E). Following CoCl_2_ treatment, the DCFH‐DA signal generated by P4CO@M + US was markedly attenuated. In comparison, Ce‐P4CO@M + US maintained substantially stronger fluorescence under the same conditions. Flow‐cytometric analysis of the corresponding eight treatment groups showed a consistent pattern (Figure 3F). These results support that Ce incorporation partially preserves ultrasound‐triggered ROS generation under hypoxia‐associated conditions.

To further evaluate the cellular hypoxic response, a hypoxia‐sensitive fluorescent probe and hypoxia‐inducible factor 1α (HIF‐1α) expression were examined. CoCl_2_ treatment markedly increased the hypoxia‐associated probe signal, whereas Ce‐P4CO@M + US produced the greatest reduction in this signal among the CoCl_2_‐treated groups (Figure 3G,I). HIF‐1α, a major regulator of cellular adaptation to oxygen deprivation and a well‐established mediator of tumor progression and therapeutic resistance [14], showed a similar response. HIF‐1α immunofluorescence was strongly increased following CoCl_2_ exposure but was reduced after Ce‐P4CO@M + US treatment (Figure 3H,J). Western blotting provided independent molecular validation, showing elevated HIF‐1α expression under CoCl_2_‐induced hypoxia‐mimetic conditions and a pronounced reduction following Ce‐P4CO@M + US treatment; densitometric analysis from three independent experiments is provided in Figure S10.

Collectively, these results demonstrate that Ce incorporation promotes H_2_O_2_ conversion and O_2_ generation, attenuates hypoxia‐associated cellular responses, and preserves ultrasound‐triggered ROS production under hypoxia‐mimetic conditions. These findings establish the functional basis for the subsequent investigation of oxidative stress and ferroptosis‐associated tumor‐cell injury.

### 2.4 Cellular Internalization and Ultrasound‐Triggered Intracellular Activation of TcEM@Ce‐P4CO@M

Having established the oxygenation‐enhanced sonodynamic activity of TcEM@Ce‐P4CO@M, we next investigated its cellular internalization and intracellular fate in 4T1 tumor cells. Cancer cell membrane‐coated nanoplatforms can retain membrane‐associated recognition and adhesion properties from their source cells, thereby facilitating homologous tumor‐associated interactions [15]. Time‐dependent confocal imaging showed a progressive increase in nanoparticle‐associated fluorescence in 4T1 cells from 1 to 12 h, indicating efficient cellular internalization of TcEM@Ce‐P4CO@M (Figure 4A). Subsequent colocalization analysis revealed partial intracellular distribution of the nanoparticles in lysosomes, the endoplasmic reticulum, and mitochondria (Figure 4B), indicating access to multiple subcellular compartments relevant to subsequent ultrasound‐triggered oxidative injury.

**FIGURE 4:**
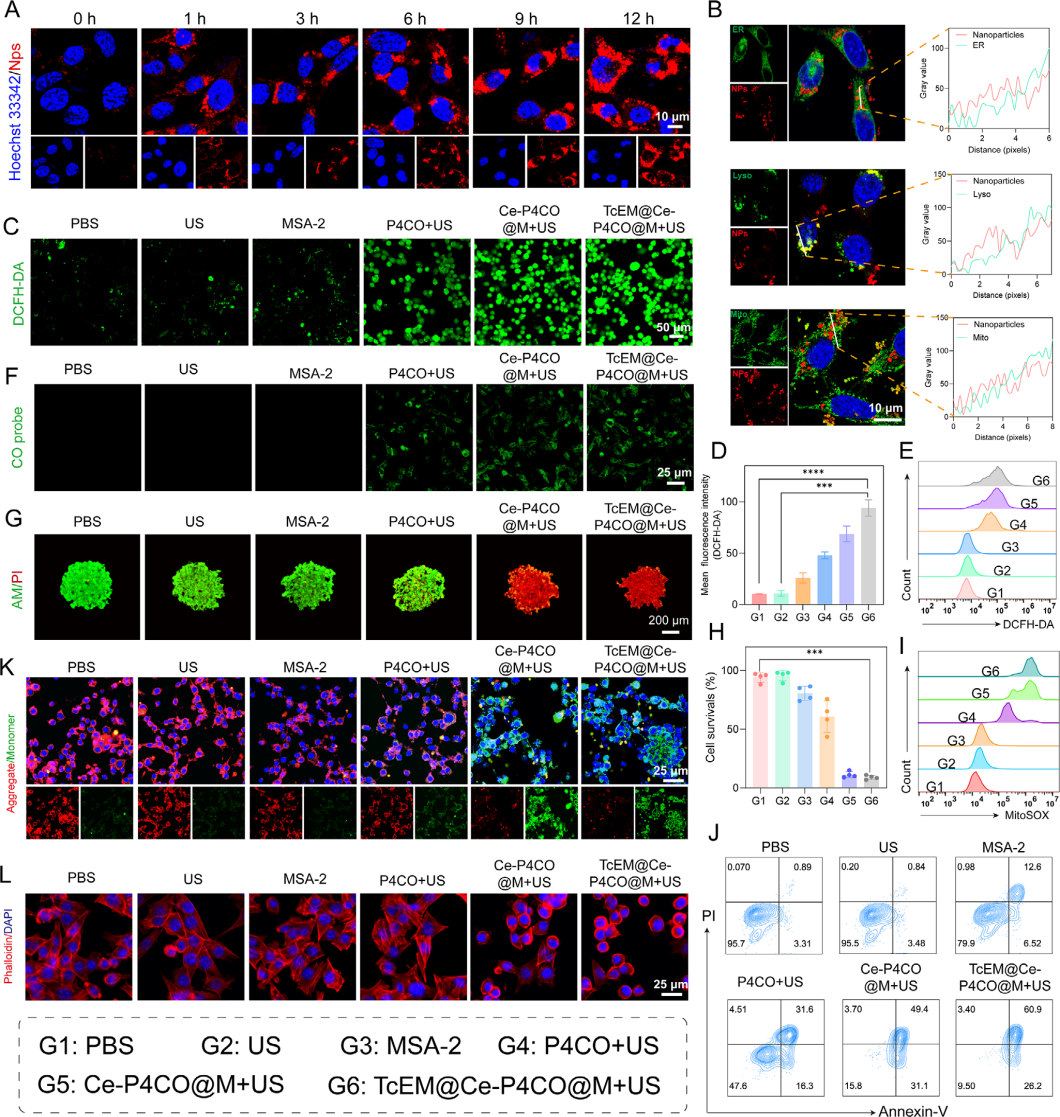
Cellular internalization and ultrasound‐triggered intracellular activation of TcEM@Ce‐P4CO@M. (A) Time‐dependent confocal fluorescence images of fluorescently labeled TcEM@Ce‐P4CO@M in 4T1 cells after incubation for 0, 1, 3, 6, 9, and 12 h. Nuclei were stained with Hoechst 33342. Scale bar: 10 µm. (B) Confocal colocalization analysis of intracellular TcEM@Ce‐P4CO@M with lysosomes, the endoplasmic reticulum (ER), and mitochondria, together with representative fluorescence line‐scan profiles. Scale bar: 10 µm. (C) Representative DCFH‐DA fluorescence images showing intracellular ROS‐associated signals after the indicated treatments. Scale bar: 50 µm. (D) Quantification of mean DCFH‐DA fluorescence intensity corresponding to (C). (E) Flow cytometric analysis of DCFH‐DA fluorescence after the indicated treatments. (F) Representative fluorescence images of intracellular CO‐associated signals detected using a CO‐sensitive probe. Scale bar: 25 µm. (G) Representative Calcein‐AM/PI fluorescence images of 3D 4T1 tumor spheroids after the indicated treatments. Scale bar: 200 µm. (H) CCK‐8 cell‐viability assay of 3D 4T1 tumor spheroids after the indicated treatments. (I) Flow cytometric analysis of mitochondrial ROS using MitoSOX Red. (J) Annexin V/PI flow cytometric analysis of cell‐death‐associated populations after the indicated treatments. (K) JC‐1 fluorescence images showing changes in mitochondrial membrane potential (ΔΨm). JC‐1 aggregates and monomers are shown in red and green, respectively. Scale bar: 25 µm. (L) Phalloidin staining of F‐actin organization after the indicated treatments. Nuclei were counterstained with DAPI. Scale bar: 25 µm. Groups: G1, PBS; G2, US; G3, MSA‐2; G4, P4CO + US; G5, Ce‐P4CO@M + US; G6, TcEM@Ce‐P4CO@M + US. Quantitative data are presented as mean ± SD. For the CCK‐8 cell‐viability assay in (H), n = 4 independent experiments; for the other quantitative assays, *n* = 3 independent experiments. Statistical significance was determined by one‐way ANOVA followed by Tukey's multiple‐comparisons test. Brackets indicate the specific group comparisons shown in the figure. ^***^ *p* < 0.001, ^****^ *p* < 0.0001.

To directly evaluate the contribution of TcEM coating and clarify the cellular preference of the nanoplatform, fluorescently labeled Ce‐P4CO@M and TcEM@Ce‐P4CO@M were further compared in homologous 4T1 tumor cells and CD8^+^ T cells. At 4°C, where energy‐dependent internalization is minimized, TcEM coating markedly increased nanoparticle association with 4T1 cells relative to Ce‐P4CO@M, whereas the corresponding enhancement in CD8^+^ T cells was comparatively modest (Figure S11A,B). Direct comparison further showed greater association of TcEM@Ce‐P4CO@M with 4T1 cells than with CD8^+^ T cells. A similar pattern was observed at 37°C, with TcEM@Ce‐P4CO@M showing substantially greater uptake by 4T1 cells than the uncoated formulation, while the increase in CD8^+^ T cells remained limited (Figure S11C,D). These results support the conclusion that TcEM coating predominantly enhances homologous tumor‐associated interaction and uptake rather than functioning as a preferential T‐cell‐targeting moiety. Accordingly, the membrane‐displayed CD86 and anti‐TIM‐3 are interpreted primarily as local immunoregulatory interfaces after tumor‐associated delivery rather than as determinants of systemic T‐cell targeting.

We next examined whether the ultrasound‐responsive CO/ROS‐generating properties established at the physicochemical level were retained following cellular internalization. DCFH‐DA imaging showed weak intracellular ROS‐associated fluorescence in the PBS, US, and MSA‐2 groups, whereas ultrasound treatment of the P4CO‐containing formulations produced markedly stronger signals (Figure 4C,D). Among these groups, TcEM@Ce‐P4CO@M + US generated the highest DCFH‐DA fluorescence, and flow‐cytometric analysis showed a consistent trend (Figure 4E). In parallel, a CO‐sensitive fluorescent probe revealed pronounced intracellular CO‐associated fluorescence after ultrasound activation of P4CO‐containing formulations, with the strongest signal observed in the TcEM@Ce‐P4CO@M + US group (Figure 4F). These results demonstrate that TcEM@Ce‐P4CO@M retains effective ultrasound‐triggered CO release and ROS generation following tumor‐cell internalization.

To evaluate treatment efficacy in a three‐dimensional tumor model, 4T1 multicellular spheroids were subsequently used. Three‐dimensional tumor spheroids reproduce several features absent from conventional monolayer cultures, including extensive cell–cell interactions, diffusion barriers, and spatial gradients of oxygen and nutrients [16]. Calcein‐AM/PI staining showed predominantly viable spheroids in the PBS, US, and MSA‐2 groups, whereas ultrasound activation of P4CO‐containing formulations progressively increased PI‐positive regions and reduced spheroid viability; consistent with these findings, Cell Counting Kit‐8 (CCK‐8) analysis showed a corresponding reduction in spheroid viability (Figure 4G,H). TcEM@Ce‐P4CO@M + US produced the most pronounced spheroid damage among the tested groups, supporting effective ultrasound‐triggered tumor‐cell injury in a three‐dimensional model.

Because CO can modulate mitochondrial respiration and redox homeostasis, while sonodynamic ROS impose additional oxidative stress [17], we next examined mitochondrial dysfunction following intracellular ultrasound activation. MitoSOX analysis showed increased mitochondrial ROS in the P4CO + US and Ce‐P4CO@M + US groups, with the strongest signal observed after TcEM@Ce‐P4CO@M + US treatment (Figure 4I). Consistently, JC‐1 staining revealed an increased monomer‐associated signal and reduced aggregate‐associated fluorescence in the TcEM@Ce‐P4CO@M + US group, indicating dissipation of the mitochondrial membrane potential (ΔΨm) (Figure 4K). These findings support pronounced mitochondrial oxidative dysfunction following ultrasound‐triggered intracellular activation.

Annexin V/PI analysis further demonstrated an increase in cell‐death‐associated populations after ultrasound‐activated nanoparticle treatment, particularly in the TcEM@Ce‐P4CO@M + US group (Figure 4J). Phalloidin staining additionally revealed reduced and disorganized filamentous actin (F‐actin) structures following treatment (Figure 4L), consistent with oxidative stress‐associated cytoskeletal remodeling [18]. Together, these cellular readouts indicate that tumor‐associated internalization and ultrasound‐triggered intracellular activation of TcEM@Ce‐P4CO@M culminate in mitochondrial dysfunction and extensive tumor‐cell injury.

Collectively, these results establish a cellular sequence in which TcEM coating promotes tumor‐associated uptake, followed by ultrasound‐triggered intracellular CO/ROS generation, mitochondrial oxidative dysfunction, and tumor‐cell injury. These findings provide the cellular basis for subsequently examining whether the resulting oxidative stress is translated into ferroptosis‐associated lipid damage.

### 2.5 Ultrasound‐Triggered ROS/CO Stress Promotes Ferroptosis Through Lipid Peroxidation and Endogenous Iron‐Homeostasis Remodeling

Following the intracellular ROS/CO generation and mitochondrial dysfunction established above, we next investigated whether this oxidative stress was translated into ferroptotic tumor‐cell injury. Ferroptosis is characterized by iron‐dependent phospholipid peroxidation together with failure of cellular antioxidant‐defense systems [19, 20]. C11‐BODIPY staining showed minimal lipid oxidation in the PBS, US, and MSA‐2 groups, whereas P4CO + US markedly increased the oxidized fluorescence signal (Figure 5A–C). A further increase was observed following Ce‐P4CO@M + US and TcEM@Ce‐P4CO@M + US treatment. Consistently, intracellular malondialdehyde (MDA), a representative product of lipid peroxidation, was elevated in the ultrasound‐activated groups, with the highest level observed after TcEM@Ce‐P4CO@M + US treatment (Figure 5D). Together, these results demonstrate progressive amplification of lipid‐peroxidative stress following ultrasound activation of the integrated nanoplatform.

**FIGURE 5:**
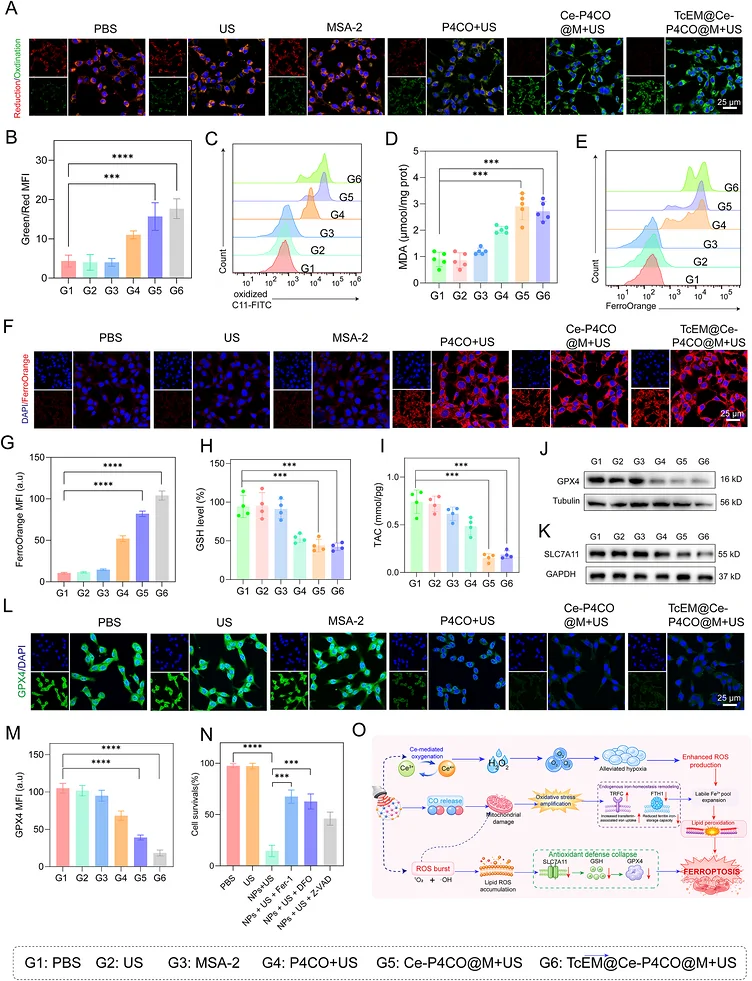
Ultrasound‐triggered ROS/CO stress promotes ferroptosis in 4T1 cells. (A) Representative C11‐BODIPY fluorescence images showing lipid oxidation after the indicated treatments. Oxidized and reduced C11‐BODIPY signals are shown in green and red, respectively. Nuclei were counterstained with DAPI. Scale bar: 25 µm. (B) Quantification of the oxidized‐to‐reduced C11‐BODIPY fluorescence ratio corresponding to (A). (C) Flow cytometric analysis of oxidized C11‐BODIPY fluorescence after the indicated treatments. (D) Intracellular malondialdehyde (MDA) levels as a biochemical readout of lipid peroxidation. (E) Flow cytometric analysis of intracellular labile Fe^2^^+^ using FerroOrange. (F) Representative FerroOrange fluorescence images after the indicated treatments. Nuclei were counterstained with DAPI. Scale bar: 25 µm. (G) Quantification of FerroOrange mean fluorescence intensity corresponding to (F). (H) Intracellular glutathione (GSH) levels after the indicated treatments. (I) Total antioxidant capacity (TAC) after the indicated treatments. (J, K) Representative Western blot analyses of GPX4 and SLC7A11, respectively, with Tubulin and GAPDH used as the corresponding loading controls. Densitometric analyses from independent experiments are provided in Figure S13. (L) Representative GPX4 immunofluorescence images after the indicated treatments. Nuclei were counterstained with DAPI. Scale bar: 25 µm. (M) Quantification of GPX4 fluorescence intensity corresponding to (L). (N) Cell‐viability rescue assay following TcEM@Ce‐P4CO@M + US treatment in the presence of ferrostatin‐1 (Fer‐1), deferoxamine (DFO), or Z‐VAD‐FMK. Groups for panel (N): PBS, US, TcEM@Ce‐P4CO@M + US, TcEM@Ce‐P4CO@M + US + Fer‐1, TcEM@Ce‐P4CO@M + US + DFO, and TcEM@Ce‐P4CO@M + US + Z‐VAD‐FMK. (O) Schematic illustration of the proposed ferroptosis‐associated mechanism integrating Ce‐mediated oxygenation, ultrasound‐triggered ROS/CO stress, endogenous iron‐homeostasis remodeling, impairment of the SLC7A11/GSH/GPX4 antioxidant‐defense axis, and lipid peroxidation. Groups for panels (A–M): G1, PBS; G2, US; G3, MSA‐2; G4, P4CO + US; G5, Ce‐P4CO@M + US; G6, TcEM@Ce‐P4CO@M + US. Data are presented as mean ± SD. For MDA measurement in (D) and TAC measurement in (I), *n* = 5 independent experiments; for GSH measurement in (H), n = 4 independent experiments; for the other quantitative assays, *n* = 3 independent experiments. Statistical significance was determined by one‐way ANOVA followed by Tukey's multiple‐comparisons test. Brackets indicate the specific group comparisons shown in the figure. ^***^ *p* < 0.001, ^****^ *p* < 0.0001.

Because P4CO simultaneously generates sonodynamic ROS and releases CO under ultrasound irradiation, we next sought to distinguish the contribution of CO from the overall sonodynamic oxidative response. Hemoglobin (Hb) was therefore introduced as a CO‐binding control. Hb markedly reduced the intracellular CO‐associated fluorescence induced by TcEM@Ce‐P4CO@M + US (Figure S12A,B). In contrast, the overall DCFH‐DA‐detectable oxidative signal remained largely preserved after Hb treatment (Figure S12C,D), indicating that bulk intracellular ROS generation was predominantly maintained by the sonodynamic process. Notably, CO attenuation significantly reduced mitochondrial ROS accumulation and C11‐BODIPY oxidation (Figure S12E–H). These findings indicate that CO is not the sole source of intracellular ROS but makes a distinct contribution to mitochondrial oxidative stress and subsequent lipid peroxidation. This interpretation is consistent with the previously reported ability of ultrasound‐triggered P4CO‐derived CO to perturb mitochondrial redox homeostasis in parallel with sonodynamic ROS generation.

We next examined the iron‐dependent component of the ferroptotic response. FerroOrange analysis revealed progressive expansion of the intracellular labile Fe^2+^ pool following P4CO + US, Ce‐P4CO@M + US, and TcEM@Ce‐P4CO@M + US treatment (Figure 5E–G). Importantly, TcEM@Ce‐P4CO@M does not contain an intentionally incorporated iron component, arguing against direct exogenous iron delivery by the nanoplatform as the source of the increased intracellular iron signal. Consistently, biochemical measurements showed concomitant increases in Fe^2+^, Fe^3+^, and total intracellular iron after ultrasound‐activated treatment (Figure S14D–F), supporting treatment‐associated remodeling of endogenous cellular iron handling rather than iron release from the nanomaterial.

To further investigate the basis of this iron accumulation, we examined transferrin receptor (TFRC) and ferritin heavy chain 1 (FTH1), which regulate complementary aspects of cellular iron acquisition and sequestration, respectively [21, 22, 23]. Western blotting and densitometric analysis showed progressively increased TFRC expression together with reduced FTH1 expression across the ultrasound‐activated treatment groups, with the most pronounced changes observed following TcEM@Ce‐P4CO@M + US treatment (Figure S14A–C). Increased TFRC, together with the elevation in total cellular iron, supports enhanced transferrin‐associated iron acquisition as a contributor to expansion of the intracellular iron pool. Meanwhile, reduced FTH1 is consistent with diminished ferritin‐associated iron sequestration, which may increase the fraction of intracellular iron accessible to the labile pool [24]. Thus, the observed Fe^2+^ accumulation is more plausibly explained by coordinated remodeling of endogenous iron uptake and storage than by direct iron release secondary to mitochondrial damage.

We subsequently examined whether the increased lipid‐peroxidative burden was accompanied by impairment of the major ferroptosis‐defense system. The SLC7A11/GSH/GPX4 axis plays a central role in suppressing phospholipid peroxidation and ferroptotic cell death [4]. Intracellular GSH content and total antioxidant capacity were progressively reduced after ultrasound‐activated treatment, with the strongest decreases observed in the TcEM@Ce‐P4CO@M + US group (Figure 5H,I). Western blotting further showed downregulation of GPX4 and SLC7A11 (Figure 5J,K), and densitometric analyses from independent experiments confirmed these changes (Figure S13). Consistently, GPX4 immunofluorescence was markedly reduced following P4CO + US treatment and decreased further in the Ce‐containing and TcEM‐coated groups (Figure 5L,M). GPX4 downregulation was also observed under CoCl_2_‐induced hypoxia‐mimetic conditions (Figure S15).

Finally, pharmacological rescue experiments were performed to assess the contribution of ferroptosis to treatment‐induced cell death. Ferrostatin‐1 (Fer‐1), an inhibitor of lipid peroxidation, and the iron chelator deferoxamine (DFO) both substantially restored cell viability following TcEM@Ce‐P4CO@M + US treatment, whereas the pan‐caspase inhibitor Z‐VAD‐FMK provided only partial protection (Figure 5N). Together with the lipid‐peroxidation, iron‐homeostasis, and GPX4/SLC7A11 data, these pharmacological rescue results support ferroptosis as a major contributor to the observed tumor‐cell killing.

Collectively, TcEM@Ce‐P4CO@M couples Ce‐mediated oxygenation with ultrasound‐triggered ROS/CO stress to amplify lipid peroxidation, remodel endogenous iron homeostasis, and impair the SLC7A11/GSH/GPX4 antioxidant defense, thereby promoting ferroptotic tumor‐cell injury (Figure 5O). We next investigated whether the mitochondrial and DNA damage associated with this oxidative injury was accompanied by activation of downstream innate immune signaling.

### 2.6 Ferroptosis‐Associated Mitochondrial and DNA Damage is Accompanied by Enhanced cGAS–STING Signaling and Immunogenic Cell Death

Having established the ferroptosis‐associated oxidative injury induced by TcEM@Ce‐P4CO@M + US, we next investigated whether this cellular stress was accompanied by mitochondrial permeability transition and DNA damage, two events that can generate damage‐associated signals relevant to downstream innate immune sensing. Mitochondrial dysfunction and DNA damage can generate endogenous nucleic‐acid danger signals that engage cytosolic DNA‐sensing pathways, including cGAS–STING signaling [25]. As summarized in Figure 6A, we therefore examined whether ultrasound‐triggered ROS/CO stress and ferroptosis‐associated injury were accompanied by mitochondrial and genomic damage followed by enhanced STING‐associated immune signaling.

**FIGURE 6:**
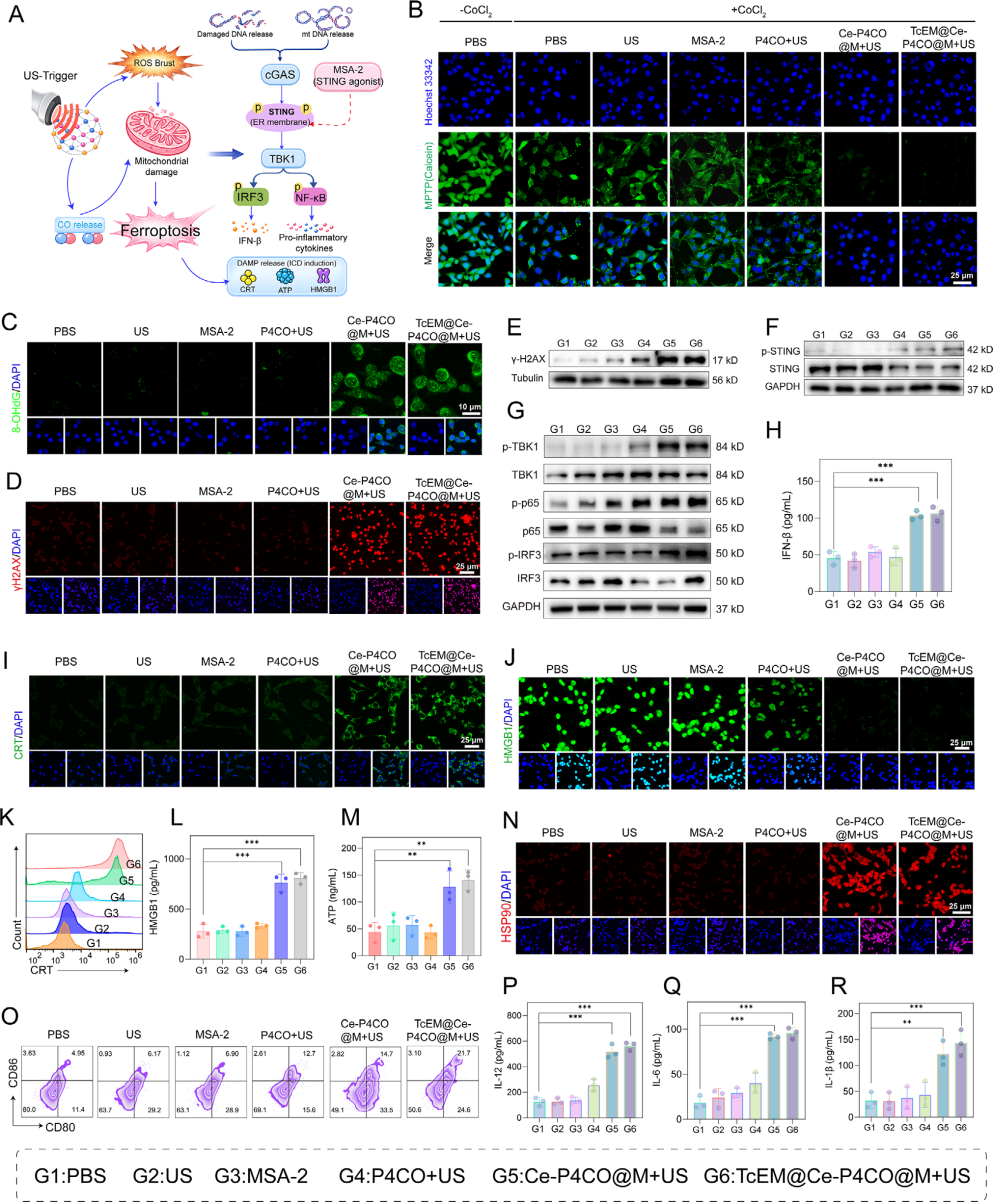
TcEM@Ce‐P4CO@M under ultrasound induces mitochondrial and DNA damage, enhances STING‐associated signaling, and promotes ICD‐associated responses and BMDC maturation. (A) Schematic illustration of the proposed sequence linking ultrasound‐triggered ROS/CO stress and ferroptosis‐associated oxidative injury with mitochondrial/DNA damage, STING‐associated signaling, DAMP exposure/release, and dendritic‐cell activation. (B) Calcein‐AM/CoCl_2_ fluorescence assay for assessment of mitochondrial permeability transition after the indicated treatments. Reduced mitochondrial calcein fluorescence is consistent with increased mPTP opening. Scale bar: 25 µm. (C, D) Representative immunofluorescence images of 8‐OHdG and γ‐H2AX, respectively, for evaluation of oxidative and genomic DNA damage. Scale bars: 10 µm in (C) and 25 µm in (D). (E) Representative Western blot analysis of γ‐H2AX. Corresponding densitometric quantification is provided in Figure S16A. (F) Representative Western blot analysis of p‐STING and total STING. Corresponding p‐STING/STING quantification is provided in Figure S16B. (G) Representative Western blot analyses of downstream STING‐associated signaling proteins, including p‐TBK1/TBK1, p‐p65/p65, and p‐IRF3/IRF3. For compact presentation, one representative GAPDH blot is shown in (G); the individual loading‐control blots are provided in the Data S1. Corresponding densitometric analyses are provided in Figure S16C–E. (H) IFN‐β concentrations in culture supernatants after the indicated treatments. (I) Representative CRT immunofluorescence images showing treatment‐associated CRT exposure. Scale bar: 25 µm. (J) Representative HMGB1 immunofluorescence images showing changes in intracellular/nuclear HMGB1 retention. Scale bar: 25 µm. (K) Flow cytometric analysis of CRT‐positive cells. (L, M) Extracellular HMGB1 and ATP concentrations, respectively. (N) Representative HSP90 immunofluorescence images after the indicated treatments. Scale bar: 25 µm. (O) Flow cytometric analysis of CD80^+^CD86^+^ BMDCs after incubation with conditioned media collected from differently treated 4T1 cells. BMDCs were not directly exposed to the therapeutic formulations. (P–R) IL‐12, IL‐6, and IL‐1β concentrations in BMDC culture supernatants, respectively. Quantitative data are presented as mean ± SD (*n* = 3 independent experiments). Groups: G1, PBS; G2, US; G3, MSA‐2; G4, P4CO + US; G5, Ce‐P4CO@M + US; G6, TcEM@Ce‐P4CO@M + US. Statistical significance was determined by one‐way ANOVA followed by Tukey's multiple‐comparisons test. Brackets indicate the specific group comparisons shown in the figure. ^*^ *p* < 0.05, ^**^ *p* < 0.01, ^***^ *p* < 0.001, ^****^ *p* < 0.0001.

Mitochondrial permeability transition was first evaluated using the calcein‐AM/CoCl_2_ assay. In this assay, Co^2+^ quenches cytosolic calcein fluorescence, whereas mitochondrial calcein remains protected when the inner mitochondrial membrane is intact. Opening of the mitochondrial permeability transition pore (mPTP) permits Co^2+^ access to mitochondrial calcein, resulting in loss of the protected mitochondrial fluorescence signal. Ultrasound activation of the P4CO‐containing formulations progressively reduced mitochondrial calcein fluorescence, with the most pronounced reduction observed in the TcEM@Ce‐P4CO@M + US group (Figure 6B), indicating enhanced mitochondrial permeability transition following treatment.

We next evaluated oxidative and genomic DNA damage. Immunofluorescence analysis showed progressively increased 8‐hydroxy‐2′‐deoxyguanosine (8‐OHdG) and γ‐H2AX signals after ultrasound‐activated treatment, with the strongest responses observed in the TcEM@Ce‐P4CO@M + US group (Figure 6C,D). Consistently, comet assays revealed increased comet‐tail formation in the ultrasound‐activated groups, indicating enhanced DNA damage (Figure S18). Western blotting further confirmed increased γ‐H2AX expression (Figure 6E), and densitometric analysis from three independent experiments verified this trend (Figure S16A). Together, these results demonstrate that ultrasound‐triggered treatment induces pronounced mitochondrial permeability transition together with oxidative and genomic DNA damage.

We then examined whether these damage‐associated responses were accompanied by enhanced STING‐associated signaling. In the canonical cytosolic DNA‐sensing pathway, cGAS recognizes aberrant cytosolic DNA and activates STING, which subsequently recruits TANK‐binding kinase 1 (TBK1) and promotes interferon regulatory factor 3 (IRF3) and nuclear factor kappa B (NF‐κB) signaling, leading to type I interferon and inflammatory responses [25]. Western blot analysis showed progressively increased STING phosphorylation after ultrasound‐activated treatment (Figure 6F). Consistently, phosphorylation of TBK1, p65, and IRF3 was markedly enhanced, with the strongest responses observed in the TcEM@Ce‐P4CO@M + US group (Figure 6G). Densitometric analyses of p‐STING/STING, p‐TBK1/TBK1, p‐p65/p65, and p‐IRF3/IRF3 from three independent experiments confirmed these changes (Figure S16B–E). In parallel, interferon beta (IFN‐β) secretion was significantly elevated following treatment, particularly in the TcEM@Ce‐P4CO@M + US group (Figure 6H), providing a functional downstream readout consistent with enhanced STING‐associated signaling.

We next examined whether this oxidative injury was accompanied by immunogenic cell death (ICD)‐associated responses. Immunogenic cell death is characterized by coordinated exposure and release of danger‐associated molecular patterns (DAMPs), including cell‐surface calreticulin (CRT), extracellular adenosine triphosphate (ATP), and high mobility group box 1 (HMGB1) release, which can promote antigen uptake and activation of antigen‐presenting cells [26]. CRT immunofluorescence showed progressively increased membrane‐associated signals following ultrasound‐activated treatment (Figure 6I), whereas HMGB1 staining showed reduced intracellular/nuclear retention in the strongly treated groups (Figure 6J). Flow‐cytometric analysis further demonstrated an increased proportion of CRT‐positive cells, with the highest level observed following TcEM@Ce‐P4CO@M + US treatment (Figure 6K). Complementary Western blot analyses showed increased cell‐associated CRT together with reduced intracellular HMGB1, and densitometric analyses confirmed these changes (Figure S17A–D).

Consistent with the reduced intracellular retention of HMGB1, extracellular HMGB1 concentrations were markedly elevated in the culture supernatants after ultrasound‐activated treatment (Figure 6L). Extracellular ATP release showed a similar increase (Figure 6M), while heat shock protein 90 (HSP90) exposure was also enhanced in the Ce‐P4CO@M + US and TcEM@Ce‐P4CO@M + US groups (Figure 6N). Together, these complementary intracellular and extracellular measurements demonstrate pronounced ICD‐associated DAMP exposure and release following ultrasound activation of the nanoplatform.

Finally, we investigated whether tumor‐cell‐derived immunogenic signals could promote dendritic‐cell maturation. Bone marrow‐derived dendritic cells (BMDCs) were incubated with conditioned media collected from differently treated 4T1 cells rather than being directly exposed to the therapeutic formulations. Dendritic‐cell maturation is accompanied by increased expression of costimulatory molecules such as CD80 and CD86 and supports efficient antigen presentation and subsequent T‐cell priming [27]. Flow‐cytometric analysis showed that conditioned media derived from ultrasound‐activated nanoparticle‐treated 4T1 cells promoted BMDC maturation, with the highest CD80^+^CD86^+^ population observed after stimulation with conditioned medium from the TcEM@Ce‐P4CO@M + US group (Figure 6O). Consistently, secretion of IL‐12, IL‐6, and IL‐1β by BMDCs was increased after stimulation with conditioned media from the same treatment group (Figures 6P–R).

Collectively, these results establish a sequential association between ferroptosis‐associated tumor‐cell injury and downstream innate immune activation. Ultrasound‐triggered ROS/CO stress induces mitochondrial permeability transition and DNA damage, accompanied by enhanced STING‐associated signaling and ICD‐associated DAMP exposure and release. The resulting tumor‐cell‐derived immunogenic signals subsequently promote BMDC maturation and inflammatory cytokine production, thereby linking localized oxidative tumor‐cell injury with downstream antitumor immune activation.

### 2.7 Dual‐Engineered TcEM Reinforces CD8^+^ T‐Cell Activation Through Complementary CD86 Costimulation and Anti‐TIM‐3 Checkpoint Modulation

Following the ICD‐associated DAMP release and BMDC maturation observed above, we next examined whether the engineered TcEM membrane could provide an additional adaptive immune‐regulatory interface for CD8^+^ T cells. Insufficient costimulatory signaling together with persistent inhibitory‐receptor signaling contributes substantially to impaired T‐cell function within the tumor microenvironment [28]. Accordingly, TcEM was engineered to integrate two complementary immune‐regulatory signals: membrane‐displayed CD86 to engage the CD28 costimulatory pathway and anti‐TIM‐3 to attenuate TIM‐3‐associated inhibitory signaling [29, 30]. Western blot analysis confirmed the expected expression of anti‐TIM‐3 and CD86 in the corresponding single‐engineered membranes and their simultaneous presence in the dual‐engineered TcEM, with Na^+^/K^+^‐ATPase used as the membrane loading control (Figure 7A). Densitometric analyses further confirmed enrichment of anti‐TIM‐3 and CD86 in the corresponding engineered membranes, with both proteins concurrently retained in TcEM (Figure S19A,B). Flow cytometry further verified increased surface CD86 expression in 4T1‐CD86 cells compared with parental 4T1 cells (Figure 7B). The proposed CD86–CD28 costimulatory and anti‐TIM‐3–TIM‐3 checkpoint‐modulatory interactions are illustrated in Figure 7C,D.

**FIGURE 7:**
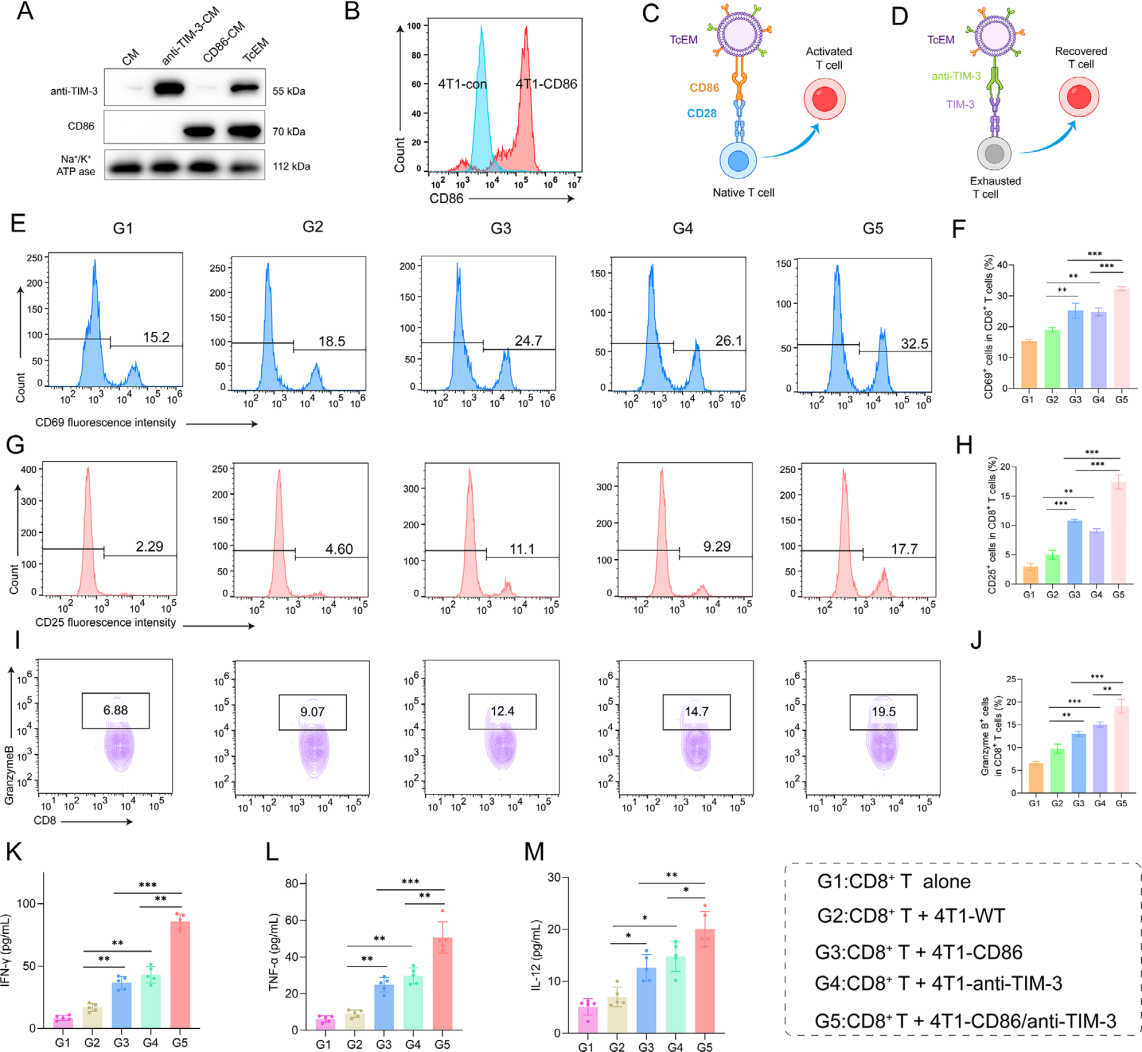
Dual‐engineered TcEM reinforces CD8^+^ T‐cell activation through CD86 costimulation and anti‐TIM‐3 checkpoint modulation. (A) Representative Western blot analysis of anti‐TIM‐3 and CD86 expression in native 4T1 cell membrane (CM), anti‐TIM‐3‐engineered membrane (anti‐TIM‐3‐CM), CD86‐engineered membrane (CD86‐CM), and dual‐engineered TcEM. Na^+^/K^+^‐ATPase was used as the membrane loading control. (B) Flow cytometric analysis of surface CD86 expression in parental 4T1 cells and CD86‐engineered 4T1 cells. (C) Schematic illustration of CD86–CD28‐mediated costimulatory signaling between the engineered membrane and T cells. (D) Schematic illustration of anti‐TIM‐3–TIM‐3‐mediated checkpoint modulation. (E) Representative flow‐cytometric histograms of CD69 expression in CD8^+^ T cells after the indicated co‐culture conditions. (F) Quantification of CD69^+^CD8^+^ T cells corresponding to (E). (G) Representative flow‐cytometric histograms of CD25 expression in CD8^+^ T cells. (H) Quantification of CD25^+^ CD8^+^ T cells corresponding to (G). (I) Representative intracellular flow‐cytometric analysis of Granzyme B in CD8^+^ T cells. (J) Quantification of Granzyme B^+^ CD8^+^ T cells corresponding to (I). (K–M) Concentrations of IFN‐γ, TNF‐α, and IL‐12, respectively, in co‐culture supernatants. Groups: G1, CD8^+^ T cells alone; G2, CD8^+^ T cells + 4T1‐WT; G3, CD8^+^ T cells + 4T1‐CD86; G4, CD8^+^ T cells + 4T1‐anti‐TIM‐3; G5, CD8^+^ T cells + dual‐engineered 4T1‐CD86/anti‐TIM‐3. Quantitative data are presented as mean ± SD (*n* = 3 independent experiments). Statistical significance was determined by one‐way ANOVA followed by Tukey's multiple‐comparisons test. Horizontal lines indicate the specific group comparisons shown in the figure. ^*^ *p* < 0.05, ^**^ *p* < 0.01, ^***^ *p* < 0.001.

To distinguish the individual contributions of CD86 costimulation and anti‐TIM‐3 checkpoint modulation, CD8^+^ T cells were evaluated using a five‐group co‐culture system: CD8^+^ T cells alone (G1), CD8^+^ T cells co‐cultured with wild‐type 4T1 cells (G2), 4T1‐CD86 cells (G3), 4T1‐anti‐TIM‐3 cells (G4), or dual‐engineered 4T1‐CD86/anti‐TIM‐3 cells (G5). CD69 expression increased from 18.5% in the wild‐type co‐culture group to 24.7% and 26.1% in the CD86‐only and anti‐TIM‐3‐only groups, respectively, and reached 32.5% in the dual‐engineered group (Figure 7E,F). A similar pattern was observed for CD25, which increased from 4.60% in G2 to 11.1% in G3 and 9.29% in G4, with the highest level of 17.7% observed in G5 (Figure 7G,H). These results indicate that both CD86‐mediated costimulation and anti‐TIM‐3‐mediated checkpoint modulation contribute to CD8^+^ T‐cell activation, while their combined presentation produces the strongest overall activation phenotype.

We next examined whether the enhanced activation phenotype was accompanied by increased cytotoxic effector potential. Granzyme B is a major cytotoxic effector protease in activated CD8^+^ T cells and plays a central role in target‐cell killing [31]. Intracellular flow cytometry showed that the proportion of Granzyme B^+^ CD8^+^ T cells increased from 9.07% in the wild‐type 4T1 co‐culture group to 12.4% and 14.7% in the CD86‐only and anti‐TIM‐3‐only groups, respectively, and further increased to 19.5% in the dual‐engineered group (Figure 7I,J). Thus, the effects of the two engineered immune‐regulatory modules were not restricted to early activation markers but were also reflected in enhanced cytotoxic effector potential.

Consistent with these cellular readouts, cytokine analysis of the co‐culture supernatants showed increased interferon‐γ (IFN‐γ)and tumor necrosis factor‐α (TNF‐α) concentrations in both single‐engineered groups relative to the wild‐type co‐culture control, with the highest levels observed in the dual‐engineered group (Figure 7K,L). IFN‐γ and TNF‐α are representative effector cytokines associated with activated cytotoxic T‐cell responses [32]. IL‐12 concentrations also showed an overall increase across the engineered co‐culture conditions, with the highest level observed in the dual‐engineered group (Figure 7M), consistent with a more type 1‐associated cytokine milieu [33]. Together, these results indicate that CD86‐mediated costimulation and anti‐TIM‐3‐mediated checkpoint modulation make distinct but complementary contributions to T‐cell activation, with dual engineering producing the strongest overall activation and effector‐associated phenotype.

Collectively, these findings establish the engineered TcEM shell as an immune‐regulatory interface that combines CD86‐mediated costimulation with anti‐TIM‐3‐mediated checkpoint modulation to reinforce CD8^+^ T‐cell activation. Together with the ICD‐associated DAMP release and dendritic‐cell maturation established in Figure 6, this adaptive immune reinforcement provides a mechanistic link between ultrasound‐triggered tumor‐cell injury and strengthened antitumor T‐cell responses.

### 2.8 In Vivo Tumor Accumulation, Primary Tumor Suppression, and Distant Tumor Challenge Inhibition by TcEM@Ce‐P4CO@M Under Ultrasound

To evaluate the in vivo tumor‐accumulation behavior of the engineered formulations, longitudinal fluorescence imaging was performed in 4T1 tumor‐bearing mice following intravenous administration of Cy5.5‐labeled formulations. Cancer‐cell‐membrane‐coated nanoparticles can retain membrane‐associated biological properties from their source cells and thereby enhance homologous tumor‐associated accumulation and retention [34]. As shown in Figure 8A, nanoparticle‐associated fluorescence progressively accumulated in the tumor region after administration. Compared with the free formulation, both Ce‐P4CO@M and TcEM@Ce‐P4CO@M exhibited stronger and more persistent tumor‐associated fluorescence. Notably, TcEM@Ce‐P4CO@M showed the highest and most sustained tumor signal throughout the observation period. Quantitative analysis further demonstrated that the tumor‐associated fluorescence of TcEM@Ce‐P4CO@M reached a maximum at approximately 12 h and remained readily detectable at later time points, whereas the free formulation showed a more rapid decline (Figure 8B). These findings support enhanced tumor‐associated accumulation and retention following nanoparticle assembly and TcEM coating.

**FIGURE 8:**
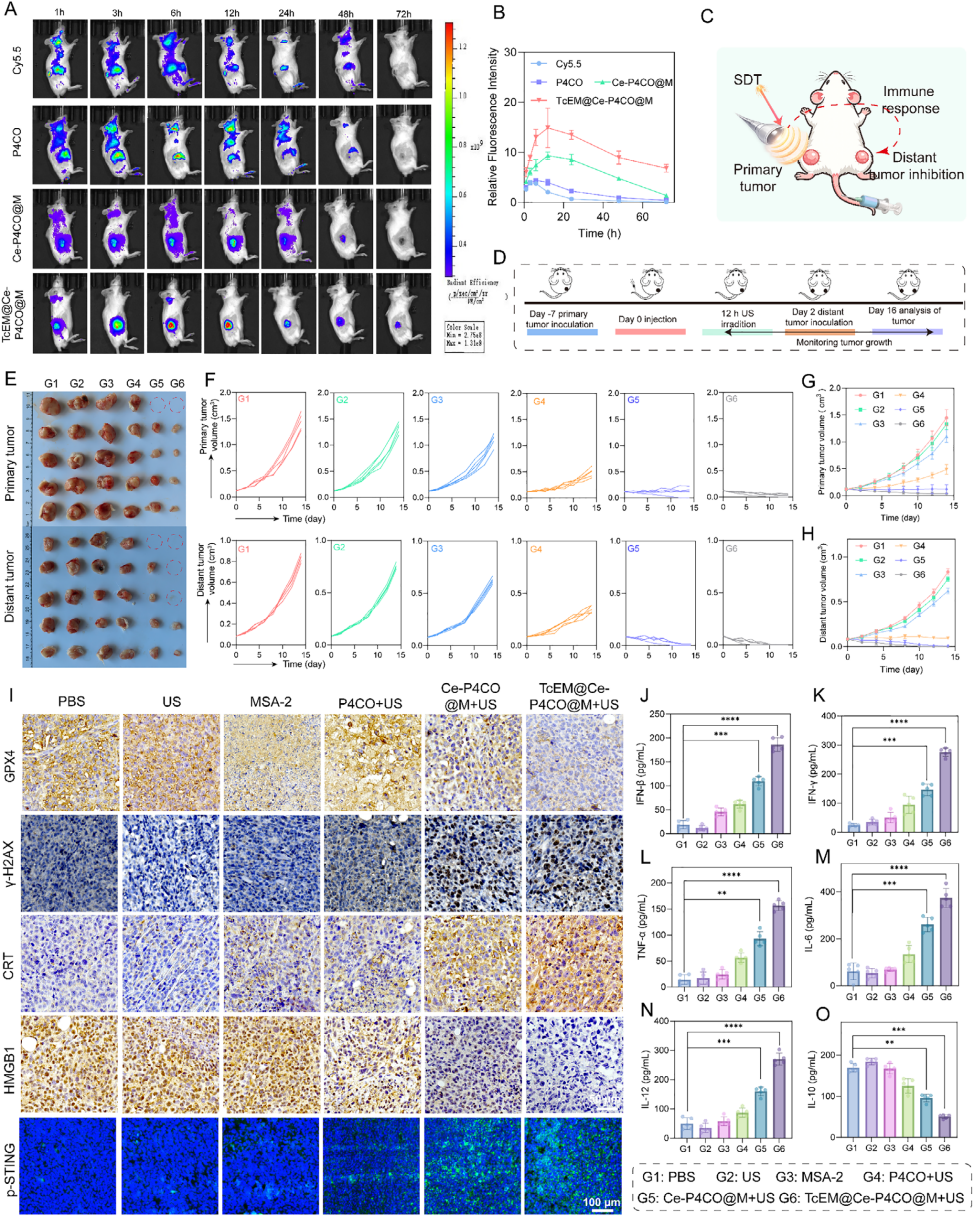
In vivo tumor accumulation, antitumor efficacy, and immune activation of TcEM@Ce‐P4CO@M under ultrasound. (A) Representative longitudinal fluorescence images of 4T1 tumor‐bearing mice at 1, 3, 6, 12, 24, 48, and 72 h after intravenous administration of Cy5.5, Cy5.5‐labeled P4CO, Ce‐P4CO@M, or TcEM@Ce‐P4CO@M. (B) Quantification of tumor‐associated fluorescence intensity over time corresponding to (A). (C) Schematic illustration of local ultrasound treatment of the primary tumor and subsequent evaluation of distant tumor challenge inhibition. (D) Experimental timeline showing primary tumor inoculation, intravenous treatment, local ultrasound irradiation, distant tumor inoculation, and endpoint analysis. (E) Photographs of excised primary and distant tumors from the indicated treatment groups at the experimental endpoint. (F) Individual growth trajectories of primary tumors (upper panels) and distant tumors (lower panels). (G, H) Mean tumor‐volume curves of primary and distant tumors, respectively. (I) Representative tumor‐tissue staining of GPX4, γ‐H2AX, CRT, HMGB1, and p‐STING after the indicated treatments. Scale bar: 100 µm. (J–O) Serum concentrations of IFN‐β, IFN‐γ, TNF‐α, IL‐6, IL‐12, and IL‐10, respectively, measured by ELISA. Groups: G1, PBS; G2, US; G3, MSA‐2; G4, P4CO + US; G5, Ce‐P4CO@M + US; G6, TcEM@Ce‐P4CO@M + US. Data are presented as mean ± SD (*n* = 5 mice per group). Longitudinal tumor‐associated fluorescence intensity in (B) and tumor‐growth data in (G, H) were analyzed using two‐way repeated‐measures ANOVA followed by Šídák's multiple‐comparisons test. Endpoint cytokine measurements in (J–O) were analyzed using one‐way ANOVA followed by Tukey's multiple‐comparisons test. ^**^ *p* < 0.01, ^***^ *p* < 0.001, ^****^ *p* < 0.0001.

Based on the tumor‐accumulation profile, we next evaluated whether local ultrasound treatment of the primary tumor could suppress both primary tumor growth and a subsequently established distant tumor challenge (Figure 8C,D). Compared with PBS, US alone, and MSA‐2, the ultrasound‐activated therapeutic formulations produced progressively greater inhibition of primary tumor growth. Among all groups, TcEM@Ce‐P4CO@M + US produced the strongest suppression of the primary tumor and also markedly inhibited growth of the non‐irradiated distant tumor (Figure 8E–H). Individual tumor‐growth trajectories and group‐averaged growth curves consistently showed the lowest tumor burdens in the G6 group at both sites. Because ultrasound irradiation was confined to the primary tumor, the concurrent inhibition of the non‐irradiated distant tumor further supports treatment‐associated systemic antitumor activity.

We next examined whether the major mechanistic responses identified in vitro were also evident in tumor tissues. Histological analysis showed reduced GPX4 staining together with increased γ‐H2AX in the TcEM@Ce‐P4CO@M + US group, consistent with enhanced ferroptosis‐associated oxidative stress and DNA damage in vivo (Figure 8I). CRT staining was increased, whereas intracellular/tumor‐associated HMGB1 staining was reduced, consistent with an ICD‐associated pattern of CRT exposure and HMGB1 release. In parallel, p‐STING staining was markedly enhanced following TcEM@Ce‐P4CO@M + US treatment, supporting increased STING‐associated signaling within the tumor microenvironment.

Consistent with the Ce‐mediated oxygenation observed in vitro, HIF‐1α protein expression in tumor tissues was reduced in the Ce‐containing treatment groups and reached the lowest level following TcEM@Ce‐P4CO@M + US treatment (Figure S20). Densitometric analysis of independent tumor samples further confirmed this trend, supporting attenuation of the hypoxia‐associated response in vivo.

We further evaluated systemic cytokine responses after treatment. Serum concentrations of IFN‐β, IFN‐γ, TNF‐α, IL‐6, and IL‐12 were increased in the ultrasound‐activated nanoparticle groups, with the most pronounced changes observed following TcEM@Ce‐P4CO@M + US treatment, whereas IL‐10 was reduced (Figure 8J–O). The increase in IFN‐β was consistent with the enhanced STING‐associated signaling observed in tumor tissues, while the overall cytokine profile indicated a shift toward a more inflammatory and immune‐active systemic environment.

Collectively, these in vivo findings demonstrate that TcEM@Ce‐P4CO@M combines enhanced tumor‐associated accumulation with potent ultrasound‐responsive antitumor activity. Local ultrasound activation suppresses primary tumor growth while concurrently inhibiting a non‐irradiated distant tumor challenge and is accompanied by ferroptosis‐associated tissue injury, DNA damage, ICD‐associated responses, enhanced STING‐associated signaling, reduced HIF‐1α expression, and systemic cytokine remodeling. Together, these findings support the conversion of localized gas‐sonodynamic tumor injury into broader systemic antitumor responses.

### 2.9 TcEM@Ce‐P4CO@M Under Ultrasound Remodels the Tumor Immune Microenvironment

To further characterize the antitumor immune response associated with TcEM@Ce‐P4CO@M‐mediated gas‐sonodynamic ferroptosis therapy, we analyzed tumor‐infiltrating lymphoid and myeloid populations in both primary and distant tumors by flow cytometry. CD8^+^ T cells represent major cytotoxic effectors of antitumor immunity, whereas CD4^+^ T cells provide critical helper functions that support the priming, expansion, and persistence of antitumor CD8^+^ T‐cell responses [35, 36]. In primary tumors, TcEM@Ce‐P4CO@M + US markedly increased the frequencies of both CD4^+^ and CD8^+^ T cells relative to the control groups (Figure 9A,B). A similar increase was observed in non‐irradiated distant tumors (Figure 9C,D). The coordinated enrichment of tumor‐infiltrating T cells at both sites further supports the systemic immune remodeling associated with the distant‐tumor inhibition observed in Figure 8.

**FIGURE 9:**
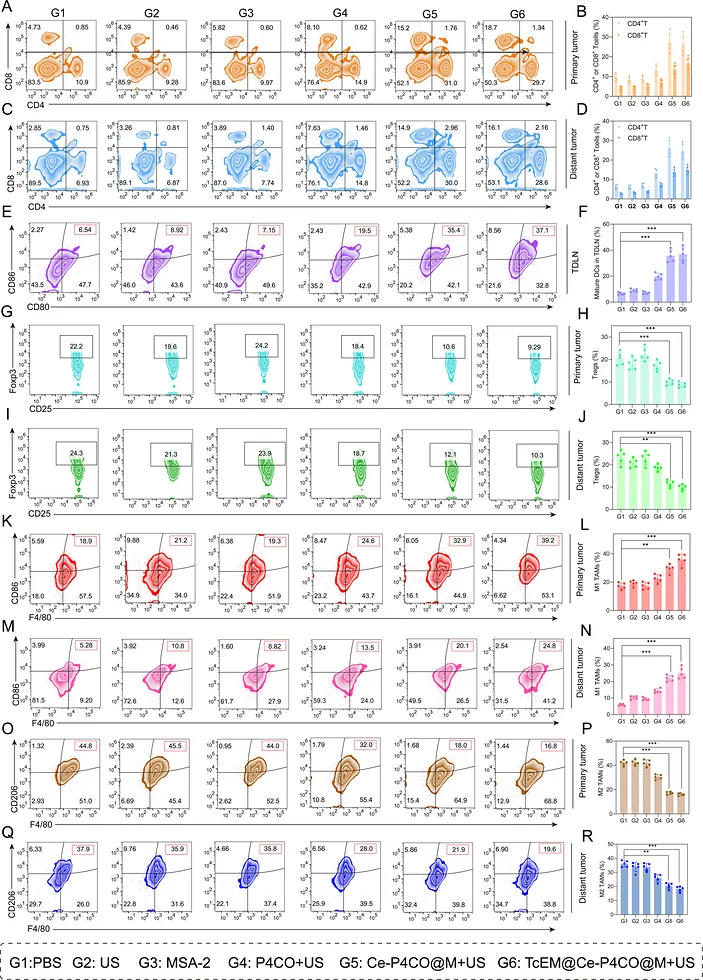
TcEM@Ce‐P4CO@M under ultrasound remodels the immune microenvironment of primary and distant tumors. (A) Representative flow‐cytometric analysis of CD4^+^ and CD8^+^ T‐cell populations in primary tumors. (B) Quantification of CD4^+^ and CD8^+^ T‐cell frequencies in primary tumors corresponding to (A). (C) Representative flow cytometric analysis of CD4^+^ and CD8^+^ T‐cell populations in distant tumors. (D) Quantification of CD4^+^ and CD8^+^ T‐cell frequencies in distant tumors corresponding to (C). (E) Representative flow‐cytometric analysis of mature dendritic cells in tumor‐draining lymph nodes (TDLNs), defined as CD80^+^CD86^+^ cells within the CD11c^+^ DC population. (F) Quantification of mature CD80^+^CD86^+^ DCs in TDLNs corresponding to (E). (G, I) Representative flow‐cytometric analysis of regulatory T cells (Tregs) in primary and distant tumors, respectively, defined as CD25^+^Foxp3^+^ cells within the CD4^+^ T‐cell population. (H, J) Quantification of Treg frequencies in primary and distant tumors corresponding to (G) and (I), respectively. (K, M) Representative flow‐cytometric analysis of CD86^+^ M1‐like TAMs within the CD11b^+^F4/80^+^ macrophage population in primary and distant tumors, respectively. (L, N) Quantification of CD86^+^ M1‐like TAM frequencies in primary and distant tumors corresponding to (K) and (M), respectively. (O, Q) Representative flow cytometric analysis of CD206^+^ M2‐like TAMs within the CD11b^+^F4/80^+^ macrophage population in primary and distant tumors, respectively. (P, R) Quantification of CD206^+^ M2‐like TAM frequencies in primary and distant tumors corresponding to (O) and (Q), respectively. Groups: G1, PBS; G2, US; G3, MSA‐2; G4, P4CO + US; G5, Ce‐P4CO@M + US; G6, TcEM@Ce‐P4CO@M + US. Data are presented as mean ± SD (n = 5 mice per group). Statistical significance was determined by one‐way ANOVA followed by Tukey's multiple‐comparisons test. Brackets indicate the specific group comparisons shown in the figure. ^**^ *p* < 0.01, ^***^ *p* < 0.001, ^****^ *p* < 0.0001.

Because the TcEM shell incorporates anti‐TIM‐3 to modulate exhaustion‐associated inhibitory signaling, we further examined programmed cell death protein 1 (PD‐1) and TIM‐3 co‐expression within tumor‐infiltrating CD8^+^ T cells. Persistent co‐expression of inhibitory receptors such as PD‐1 and TIM‐3 is frequently associated with dysfunctional or exhaustion‐related CD8^+^ T‐cell states in tumors. TcEM@Ce‐P4CO@M + US significantly reduced the frequency of PD‐1^+^TIM‐3^+^ cells within the CD8^+^ T‐cell population in both primary and distant tumors (Figure S21). These findings indicate attenuation of an exhaustion‐associated CD8^+^ T‐cell phenotype in vivo and are consistent with the checkpoint‐modulatory function of the engineered TcEM membrane.

We next examined dendritic‐cell maturation in tumor‐draining lymph nodes (TDLNs). Upregulation of the costimulatory molecules CD80 and CD86 is a characteristic feature of activated/mature dendritic cells and supports efficient antigen presentation and T‐cell priming. Flow‐cytometric analysis showed a pronounced increase in the CD80^+^CD86^+^ population within CD11c^+^ DCs following TcEM@Ce‐P4CO@M + US treatment, with the highest frequency observed in the G6 group (Figure 9E,F). This in vivo response is consistent with the ICD‐associated DAMP exposure/release and conditioned‐medium‐induced BMDC maturation observed in Figure 6, indicating enhanced antigen‐presenting‐cell activation following ultrasound‐triggered tumor injury.

We subsequently evaluated regulatory T cells (Tregs), defined as CD25^+^Foxp3^+^ cells within the CD4^+^ T‐cell population. TcEM@Ce‐P4CO@M + US markedly reduced the frequency of Tregs in both primary and distant tumors compared with the control groups (Figure 9G–J). Together with the increased CD4^+^ and CD8^+^ T‐cell infiltration and reduced frequency of PD‐1^+^TIM‐3^+^ CD8^+^ T cells, these changes indicate a shift in the lymphoid compartment toward a less immunosuppressive and more immune‐active tumor microenvironment.

Tumor‐associated macrophage (TAM) composition was further examined because macrophage states can profoundly influence antitumor immunity [37]. Within the CD11b^+^F4/80^+^ TAM population, CD86^+^ cells were operationally defined as M1‐like macrophages, whereas CD206^+^ cells were defined as M2‐like macrophages. TcEM@Ce‐P4CO@M + US increased the frequency of CD86^+^ M1‐like TAMs in both primary and distant tumors (Figure 9K–N). In contrast, the frequency of CD206^+^ M2‐like TAMs progressively decreased following ultrasound‐activated nanoparticle treatment, with the lowest levels observed in the TcEM@Ce‐P4CO@M + US group (Figure 9O–R). These changes indicate a treatment‐associated shift in TAM composition toward a more pro‐inflammatory phenotype.

Collectively, these immune‐profiling results demonstrate that TcEM@Ce‐P4CO@M + US coordinately remodels both lymphoid and myeloid compartments within the tumor microenvironment. Enhanced CD4^+^ and CD8^+^ T‐cell infiltration, reduced exhaustion‐associated CD8^+^ T cells and Tregs, increased DC maturation, and a shift toward a more pro‐inflammatory TAM phenotype were observed following treatment. Similar immune changes in non‐irradiated distant tumors further support treatment‐associated systemic immune remodeling and provide an immunological basis for the inhibition of both primary and distant tumors. The corresponding gating strategies for the analyzed immune‐cell populations are provided in Figure S22.

### 2.10 TcEM@Ce‐P4CO@M Under Ultrasound Suppresses Primary Tumor Growth and Lung Metastasis

To further evaluate whether TcEM@Ce‐P4CO@M‐mediated therapy could suppress metastatic tumor burden beyond the locally treated primary tumor, an experimental lung metastasis model was established using 4T1‐Luc cells. Intravenous injection of luciferase‐expressing 4T1 cells provides a widely used model for evaluating breast cancer cell lung colonization and experimental metastatic burden, although it bypasses several early steps of the spontaneous metastatic cascade [38]. Primary 4T1 tumors were first established subcutaneously, followed by intravenous administration of 4T1‐Luc cells on day 0. The indicated formulations were administered on days 3, 7, 11, and 18, with ultrasound irradiation restricted to the primary tumor 12 h after each administration (Figure 10A). This design enabled simultaneous assessment of local primary‐tumor control and treatment‐associated effects on experimentally established pulmonary metastatic burden.

**FIGURE 10:**
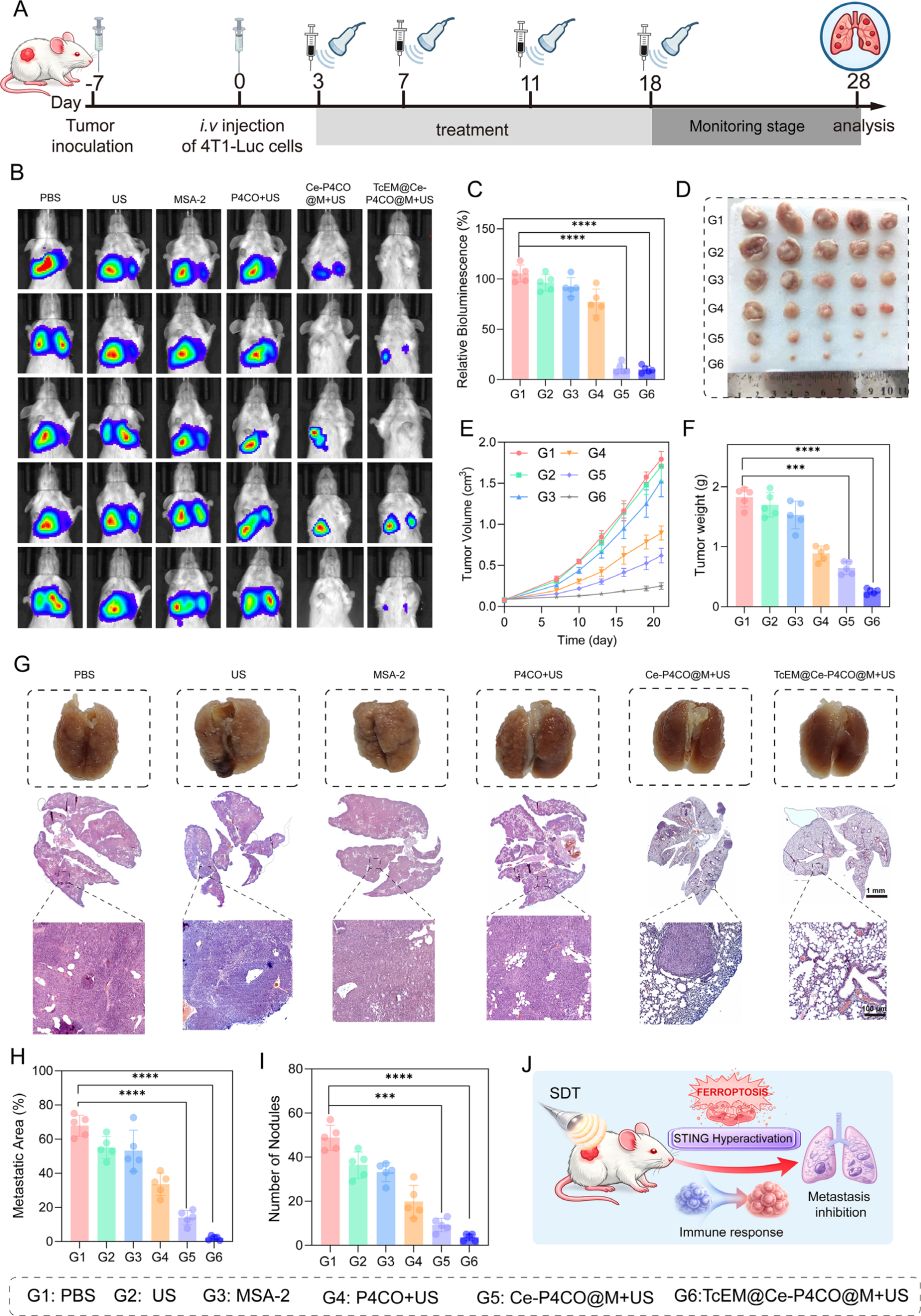
TcEM@Ce‐P4CO@M under ultrasound suppresses primary tumor growth and lung metastasis. (A) Experimental timeline of primary 4T1 tumor establishment, intravenous injection of 4T1‐Luc cells, therapeutic administration, local ultrasound treatment of the primary tumor, and endpoint lung analysis. Formulations were administered on days 3, 7, 11, and 18, followed by ultrasound irradiation of the primary tumor 12 h after each administration; lungs were analyzed on day 28. (B) Representative in vivo bioluminescence images showing pulmonary 4T1‐Luc burden after the indicated treatments. (C) Quantification of relative lung‐associated bioluminescence intensity corresponding to (B). (D) Photographs of excised primary tumors at the experimental endpoint. (E) Mean primary‐tumor growth curves during treatment. (F) Final primary‐tumor weights at the endpoint. (G) Representative gross lung images and H&E‐stained lung sections showing pulmonary metastatic lesions after the indicated treatments. (H) Quantification of metastatic area in lung sections corresponding to (G). Scale bars: 1 mm for whole‐lung H&E sections and 100 µm for magnified H&E images. (I) Quantification of pulmonary metastatic nodule number. (J) Schematic illustration of the proposed relationship among ultrasound‐triggered tumor injury, ferroptosis‐associated stress, enhanced STING‐associated immune signaling, systemic antitumor responses, and reduced pulmonary metastatic burden. Groups: G1, PBS; G2, US; G3, MSA‐2; G4, P4CO + US; G5, Ce‐P4CO@M + US; G6, TcEM@Ce‐P4CO@M + US. Data are presented as mean ± SD (n = 5 mice per group). Primary‐tumor growth data in (E) were analyzed using two‐way repeated‐measures ANOVA followed by Šídák's multiple‐comparisons test. Endpoint measurements in (C), (F), (H), and (I) were analyzed using one‐way ANOVA followed by Tukey's multiple‐comparisons test. ^***^ *p* < 0.001, ^****^ *p* < 0.0001.

In vivo bioluminescence imaging was used to assess pulmonary 4T1‐Luc burden. Strong thoracic bioluminescence signals were observed in the PBS, US, and MSA‐2 groups, whereas progressively lower signals were detected in the ultrasound‐activated therapeutic groups (Figure 10B,C). TcEM@Ce‐P4CO@M + US produced the greatest reduction in lung‐associated bioluminescence, indicating a markedly lower pulmonary metastatic burden. These findings support a treatment‐associated suppression of experimental lung colonization/metastatic outgrowth.

We simultaneously evaluated therapeutic efficacy against the primary subcutaneous tumors. PBS, US alone, and MSA‐2 showed limited suppression of primary tumor progression, whereas P4CO + US and Ce‐P4CO@M + US produced progressively stronger growth inhibition (Figure 10D–F). Among all treatment groups, TcEM@Ce‐P4CO@M + US showed the strongest primary‐tumor suppression, as reflected by smaller excised tumors, slower tumor‐volume progression, and lower final tumor weight. These results are consistent with the potent local antitumor activity of the integrated biomimetic nanoplatform under ultrasound activation.

The reduction in pulmonary metastatic burden was further confirmed by gross and histological analyses. Lungs from the control groups exhibited extensive metastatic lesions and substantial disruption of normal pulmonary architecture, whereas lungs collected from TcEM@Ce‐P4CO@M + US‐treated mice showed markedly fewer visible metastatic nodules and better‐preserved alveolar structures (Figure 10G). Quantitative analyses further demonstrated significant reductions in both metastatic area and metastatic nodule number in the G6 group (Figure 10H,I), providing independent validation of the bioluminescence findings.

The simultaneous control of the locally irradiated primary tumor and experimentally introduced pulmonary tumor burden is consistent with the broader systemic antitumor activity observed in the bilateral‐tumor and immune‐profiling experiments. Local tumor injury capable of inducing ICD and DAMP release can function in an in situ vaccination‐like manner by facilitating tumor‐antigen uptake, dendritic‐cell activation, and subsequent T‐cell priming [39]. In the present system, ultrasound‐triggered ROS/CO stress, Ce‐mediated preservation of oxidative activity, ferroptosis‐associated tumor‐cell injury, STING‐associated signaling, and TcEM‐mediated immune regulation collectively provide a plausible basis for enhanced systemic antitumor responses (Figure 10J).

Collectively, these findings demonstrate that TcEM@Ce‐P4CO@M + US not only suppresses primary tumor growth but also substantially reduces experimental pulmonary metastatic burden. Together with the distant‐tumor inhibition and immune remodeling observed above, these results support the capacity of the integrated nanoplatform to extend localized ultrasound‐triggered tumor injury into broader systemic antitumor activity.

### 2.11 Transcriptomic Reprogramming and Immune Landscape Remodeling Induced by TcEM@Ce‐P4CO@M Under Ultrasound

To further characterize the molecular changes associated with the antitumor activity of TcEM@Ce‐P4CO@M + US, RNA sequencing was performed on whole‐tumor tissues collected from the PBS and TcEM@Ce‐P4CO@M + US groups. Principal component analysis (PCA) showed clear separation between the two groups, indicating distinct treatment‐associated transcriptomic profiles (Figure 11A). Analysis of detected transcripts identified 23,978 genes shared between the two groups, together with 2,354 and 1,947 genes uniquely detected in the TcEM@Ce‐P4CO@M + US and PBS groups, respectively (Figure 11B). Consistently, differential‐expression analysis identified distinct populations of significantly upregulated and downregulated genes following treatment (Figure 11C).

**FIGURE 11:**
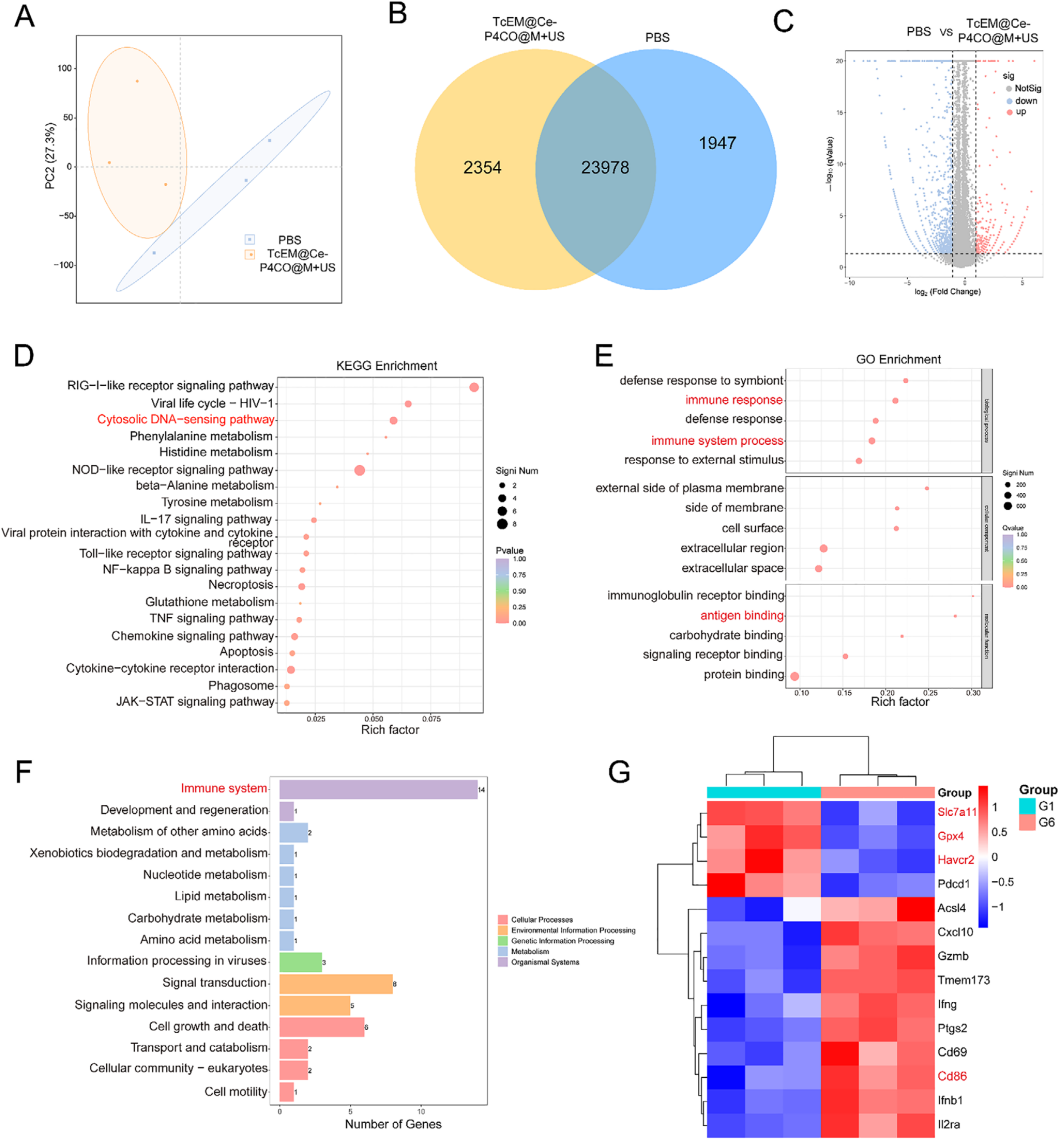
Transcriptomic profiling reveals ferroptosis‐ and immune‐associated reprogramming after TcEM@Ce‐P4CO@M + US treatment. (A) Principal component analysis (PCA) of whole‐tumor RNA‐seq profiles from the PBS and TcEM@Ce‐P4CO@M + US groups. (B) Venn diagram showing shared and group‐specific detected genes between the two groups. (C) Volcano plot showing differentially expressed genes between PBS‐ and TcEM@Ce‐P4CO@M + US‐treated tumors. Red and blue dots represent significantly upregulated and downregulated genes, respectively, whereas gray dots indicate genes without significant differential expression. (D) KEGG pathway enrichment analysis of differentially expressed genes, highlighting immune‐, inflammation‐, and DNA‐sensing‐associated pathways. (E) Gene Ontology (GO) enrichment analysis of differentially expressed genes, including representative biological process, cellular component, and molecular function categories. (F) Functional classification of differentially expressed genes according to KEGG pathway categories. (G) Heatmap of representative genes associated with ferroptosis‐related antioxidant defense and lipid metabolism, STING/interferon‐associated signaling, immune activation, T‐cell effector responses, and inhibitory‐receptor signaling. RNA‐seq analyses were performed using independent tumor samples from the PBS (G1) and TcEM@Ce‐P4CO@M + US (G6) groups (n = 3 biological samples per group).

To determine the biological processes associated with these transcriptional changes, Kyoto Encyclopedia of Genes and Genomes (KEGG) and Gene Ontology (GO) enrichment analyses were performed. KEGG analysis revealed enrichment of multiple immune‐ and inflammation‐associated pathways, including the cytosolic DNA‐sensing pathway, cytokine–cytokine receptor interaction, chemokine signaling, NF‐κB signaling, and Janus kinase‐signal transducer and activator of transcription (JAK‐STAT) signaling (Figure 11D). In particular, enrichment of the cytosolic DNA‐sensing pathway was consistent with the enhanced STING‐associated signaling identified at the protein and functional levels in the preceding experiments. GO enrichment analysis further highlighted immune response, immune system process, defense response, antigen binding, and other immune‐associated biological functions (Figure 11E). Functional classification of the differentially expressed genes similarly revealed prominent changes in immune‐system‐ and signal‐transduction‐related categories (Figure 11F). Together, these analyses demonstrate broad immune‐associated transcriptional reprogramming within TcEM@Ce‐P4CO@M + US‐treated tumors.

We next examined representative genes associated with the major therapeutic processes identified experimentally. The targeted heatmap showed reduced expression of the ferroptosis‐protective antioxidant genes Slc7a11 and Gpx4, together with increased expression of Acsl4 and Ptgs2, consistent with enhanced ferroptosis susceptibility and lipid‐peroxidative stress (Figure 11G) [40, 41]. These transcriptional changes aligned with the experimentally observed impairment of the SLC7A11/GSH/GPX4 antioxidant axis and enhanced lipid peroxidation. In parallel, increased expression of Tmem173, Ifnb1, and Cxcl10 revealed a STING/interferon‐associated transcriptional signature consistent with the enhanced STING‐associated signaling observed in Figures 6 and 8. Immune activation was further reflected by increased Cd86 expression together with elevated T‐cell activation and effector‐associated genes, including Cd69, Il2ra, Ifng, and Gzmb. Conversely, the inhibitory‐receptor genes Havcr2 and Pdcd1 were reduced following treatment. These coordinated transcriptional changes further support a shift toward a more immune‐active tumor microenvironment.

Gene set enrichment analysis provided additional support for these immune‐associated transcriptional changes. Enrichment of antigen‐binding and phagocytosis/recognition‐related gene sets was observed in TcEM@Ce‐P4CO@M + US‐treated tumors (Figure S23), further indicating enhanced transcriptional programs related to antigen recognition and innate immune processing.

Collectively, transcriptomic profiling revealed coordinated changes in ferroptosis‐associated, DNA‐sensing/interferon, antigen‐processing, and adaptive immune‐response programs following TcEM@Ce‐P4CO@M + US treatment. These molecular signatures closely aligned with the experimentally observed ferroptosis‐associated injury, STING‐associated signaling, dendritic‐cell maturation, T‐cell activation, and tumor immune remodeling, providing transcriptomic support for the integrated antitumor mechanism of the nanoplatform.

## 3 Conclusion

In summary, we developed an ultrasound‐responsive Ce(IV)‐integrated biomimetic nanosystem, TcEM@Ce‐P4CO@M, that coordinates hypoxia relief, gas‐sonodynamic ferroptosis, STING‐associated innate immune activation, and engineered T‐cell regulation within a single therapeutic platform. The nanosystem was constructed through Ce(IV)‐mediated coordination assembly of the sonosensitizer/CO donor P4CO and the STING agonist MSA‐2, followed by coating with genetically engineered tumor‐derived membranes displaying CD86 and anti‐TIM‐3. This integrated design endowed TcEM@Ce‐P4CO@M with favorable colloidal stability, homologous tumor‐associated delivery, ultrasound‐triggered CO release and ROS generation, and Ce^4+^/Ce^3+^ redox cycling‐mediated oxygenation.

Mechanistically, Ce‐mediated catalytic oxygenation attenuated hypoxia‐associated responses and sustained oxygen‐dependent sonodynamic ROS generation under oxygen‐deficient conditions. Concurrent ultrasound‐triggered ROS and CO stress promoted mitochondrial oxidative dysfunction and lipid peroxidation, while treatment‐associated remodeling of endogenous iron homeostasis, characterized by increased TFRC and reduced FTH1 expression, provided a plausible basis for expansion of the intracellular labile iron pool. Together with impairment of the SLC7A11/GSH/GPX4 antioxidant defense, these changes promoted ferroptosis‐associated tumor‐cell injury. The resulting mitochondrial and DNA damage was accompanied by enhanced STING‐associated signaling and ICD‐associated DAMP release, while co‐delivered MSA‐2 further reinforced STING‐mediated innate immune activation, contributing to an immunogenic environment that supports dendritic‐cell maturation. In parallel, the engineered TcEM shell provided CD86‐mediated costimulation and anti‐TIM‐3‐mediated checkpoint modulation, thereby strengthening CD8^+^ T‐cell activation and cytotoxic effector responses.

In vivo, TcEM@Ce‐P4CO@M exhibited enhanced tumor accumulation and retention and, in combination with local ultrasound irradiation, effectively suppressed primary tumors, inhibited non‐irradiated distant tumors, and reduced experimental lung metastasis. Immune profiling further demonstrated increased CD4^+^ and CD8^+^ T‐cell infiltration, enhanced DC maturation, reduced Treg‐associated immunosuppression, and a shift in TAM composition toward a more pro‐inflammatory phenotype. Transcriptomic analysis further supported ferroptosis‐related stress, DNA‐sensing and inflammatory pathway activation, and broader remodeling of the tumor immune landscape. Collectively, these findings demonstrate that TcEM@Ce‐P4CO@M converts hypoxia‐limited local sonodynamic injury into a coordinated ferroptosis‐linked innate‐to‐adaptive antitumor immune response, providing a strategy for integrating local oxidative therapy with systemic immune control of tumor progression and metastasis.

## 4 Experimental Section

### 4.1 Materials

P4CO was prepared in‐house using a modified porphyrin derivatization procedure. Fluorescent probes and cell‐analysis reagents, including DCFH‐DA, MitoSOX Red, C11‐BODIPY 581/591, and the Annexin V‐FITC/PI apoptosis detection kit, were obtained from Beyotime. CCK‐8 and Calcein‐AM/PI staining kits were supplied by Yeasen. ELISA kits for HMGB1, IFN‐β, IFN‐γ, TNF‐α, IL‐6, IL‐12, IL‐1β, and IL‐10 were obtained from Elabscience. JC‐1, FerroOrange, and MSA‐2 were purchased from MCE. Antibodies against GPX4, SLC7A11, TIM‐3, CD86, p‐TBK1, and TBK1 were purchased from ABclonal, China.

TFRC and FTH1 were purchased from Abcam. Anti‐HIF‐1α antibody, anti‐γH2AX antibody, Na^+^/K^+^‐ATPase antibody, CD45‐PerCP‐Cy5.5, CD3‐FITC, CD4‐PE, CD8‐APC, CD11c‐FITC, CD80‐APC, and CD86‐PE were purchased from Proteintech, China. PD‐1‐BV421 and TIM‐3‐PE‐Cy7 antibodies were used for exhausted CD8^+^ T‐cell analysis. CD11b‐PE‐Cy7, F4/80‐APC, CD86‐PE, and CD206‐BV421 antibodies were used for macrophage polarization analysis. CD25‐Alexa Fluor 647 and Foxp3‐APC antibodies were used for Treg analysis. All fluorophore‐conjugated antibodies were used according to the manufacturers’ instructions.

### 4.2 Cell Culture

The 4T1 murine breast cancer cell line (RRID: CVCL_0125) was obtained from ATCC. Luciferase‐expressing 4T1 cells (4T1‐Luc) were generated from parental 4T1 cells by stable transduction with a luciferase‐expressing vector and antibiotic selection. All cell lines were authenticated by STR profiling and tested negative for mycoplasma contamination using the MycoAlert Kit (Lonza). Cells were maintained in RPMI 1640 medium with 10% heat‐inactivated fetal bovine serum and 1% penicillin‐streptomycin under standard conditions (37°C, 5% CO_2_, 95% humidity).

### 4.3 Generation of Engineered 4T1 Cell Membranes

4T1 cells were engineered to express membrane‐associated CD86, a membrane‐associated anti‐TIM‐3 single‐chain variable fragment (scFv), or both molecules. Stable 4T1‐CD86, 4T1‐anti‐TIM‐3, and dual‐engineered 4T1‐CD86/anti‐TIM‐3 cells were generated using the corresponding expression vectors followed by antibiotic selection. Surface CD86 expression and membrane‐associated anti‐TIM‐3 were verified by flow cytometry and Western blotting.

For membrane preparation, engineered cells were harvested, washed with cold PBS, and disrupted in membrane‐extraction buffer containing protease inhibitors. Membrane fractions were collected after mechanical homogenization and differential centrifugation, washed, and extruded through polycarbonate membranes to obtain nanoscale engineered tumor‐cell membrane vesicles (TcEM). Membrane‐protein retention was evaluated by SDS‐PAGE and Western blotting.

### 4.4 Preparation of Ce‐P4CO@M and TcEM@Ce‐P4CO@M

P4CO and MSA‐2 were separately dissolved in DMSO at 1 mg mL^−1^ and mixed at a feeding mass ratio of 1:1. The mixture was then added dropwise into an aqueous ammonium cerium(IV) nitrate (CAN) solution (1.5 mg mL^−1^) under stirring. After reaction for 12 h in the dark, Ce‐P4CO@M was collected by centrifugation (10,000 × g, 10 min) and washed to remove unassembled components.For membrane coating, TcEM vesicles were mixed with Ce‐P4CO@M at a membrane protein‐to‐core mass ratio of 1:2 and extruded through 200 nm polycarbonate membranes to obtain TcEM@Ce‐P4CO@M. The resulting nanoparticles were purified and resuspended in PBS for further use.

### 4.5 Physicochemical Characterization and MSA‐2 Loading

Nanoparticle morphology was characterized by TEM, whereas hydrodynamic size, PDI, and zeta potential were measured by DLS. Elemental distribution and Ce valence states were analyzed by HAADF‐STEM/EDS and XPS, respectively. UV–vis and FT–IR spectroscopy were used for complementary structural characterization.

MSA‐2 loading was quantified by HPLC using a C18 column with an acetonitrile/water mobile phase containing 0.1% formic acid and UV detection at 258 nm. Purified TcEM@Ce‐P4CO@M was disrupted to release the incorporated MSA‐2, which was quantified against an MSA‐2 calibration curve. The MSA‐2 loading amount was approximately 80 µg mg^−1^ nanoparticles, corresponding to a loading capacity of approximately 8.0 wt.% and an encapsulation efficiency of approximately 80%. Colloidal stability was evaluated in PBS, DMEM, and DMEM containing 10% FBS by monitoring particle size, PDI, and zeta potential.

### 4.6 Ultrasound‐Triggered CO Release and ROS Generation

Ultrasound‐triggered CO release was evaluated using a myoglobin‐based spectroscopic assay. Unless otherwise indicated, samples were irradiated at 1.0 MHz and 50% duty cycle. Ultrasound power densities of 0.5–1.5 W cm^−2^ and different irradiation times were used for power‐ and time‐dependent measurements. CO release was quantified from the corresponding spectroscopic response.

ROS generation was evaluated using DPBF, SOSG, methylene blue (MB), and ESR spin‐trapping assays. DPBF and MB absorbance changes were monitored at 410 and 665‐ nm, respectively. For SOSG measurements, NaN_3_ was included as a singlet‐oxygen quenching control. DTNB‐based measurements were additionally used to evaluate ultrasound‐induced thiol oxidation.

### 4.7 Ce‐Mediated H_2_O_2_ Decomposition and O_2_ Generation

P4CO@M or Ce‐P4CO@M (100 µg mL^−1^) was incubated with H_2_O_2_ (10 mM) at 37°C. Residual H_2_O_2_ was quantified at predetermined time points using a commercial H_2_O_2_ assay kit (YEASEN, Cat. No. 60743ES50) according to the manufacturer's instructions, whereas dissolved O_2_ was measured using a dissolved‐oxygen meter. PBS, H_2_O_2_ alone, P4CO@M + H_2_O_2_, and Ce‐P4CO@M + H_2_O_2_ were included as control groups.

### 4.8 Cellular Uptake, Cellular Preference, and Subcellular Localization

For time‐dependent cellular uptake, fluorescently labeled TcEM@Ce‐P4CO@M was incubated with 4T1 cells for the indicated periods and visualized by CLSM. Subcellular localization was evaluated using lysosomal, endoplasmic reticulum, and mitochondrial trackers.

To compare tumor‐cell and T‐cell interactions, Ce‐P4CO@M and TcEM@Ce‐P4CO@M were labeled with DiD and incubated with either 4T1 cells or CD8^+^ T cells. For the cellular‐association assay, cells were incubated with the nanoparticles at 4°C for 30 min to minimize active internalization. For the uptake assay, cells were incubated at 37°C for 2 h. After washing, DiD‐positive cells were quantified by flow cytometry. Gates were defined using the corresponding unstained controls and applied identically to matched treatment groups. Experiments were independently repeated three times.

### 4.9 Cellular Treatment, Hypoxia, and Mitochondrial Stress

Unless otherwise specified, 4T1 cells were incubated with the indicated formulations at an equivalent P4CO concentration of 20 µg mL^−1^ for 6 h and subsequently irradiated with ultrasound (1.0 MHz, 1.0 W cm^−2^, 50% duty cycle, 3 min). For hypoxia‐related experiments, cells were pretreated with CoCl_2_ (100 µM, 12 h).

Intracellular ROS and CO were detected using DCFH‐DA (10 µM, 30 min) and a CO‐sensitive fluorescent probe (5 µM, 30 min), respectively. Cellular hypoxia was evaluated using an oxygen‐sensitive probe, and HIF‐1α expression was assessed by immunofluorescence and Western blotting. Mitochondrial ROS and membrane potential were analyzed using MitoSOX Red and JC‐1, respectively. Annexin V/PI staining and phalloidin staining were used to evaluate cell death and cytoskeletal injury.

### 4.10 Three‐Dimensional Tumor Spheroid Assay

4T1 tumor spheroids were generated in low‐adhesion plates using 8 × 10^3^ cells per well. After formation of stable spheroids, the indicated treatments and ultrasound irradiation were applied. Cell viability was assessed by Calcein‐AM/PI staining followed by CLSM imaging.

### 4.11 Ferroptosis and Endogenous Iron‐Homeostasis Assays

Ferroptosis‐related responses were examined following the treatment conditions described above. Lipid peroxidation was assessed using C11‐BODIPY 581/591 (5 µM, 30 min) by fluorescence microscopy and flow cytometry, whereas intracellular labile Fe^2+^was detected using FerroOrange (1 µM, 30 min). At 12 h after treatment, MDA, GSH, Fe^2+^, Fe^3+^, total iron, and total antioxidant capacity were measured using commercial assay kits according to the manufacturers’ protocols.

To investigate treatment‐associated remodeling of cellular iron homeostasis, TFRC and FTH1 protein expression was determined by Western blotting. SLC7A11 and GPX4 were analyzed in parallel as ferroptosis‐associated antioxidant‐defense proteins. GPX4 was additionally evaluated by immunofluorescence staining.

For pharmacological rescue experiments, cells were pretreated with Fer‐1 (1 µM), DFO (100 µM), or Z‐VAD‐FMK (20 µM) for 2 h before TcEM@Ce‐P4CO@M + US treatment. Cell viability was measured by CCK‐8 at 24 h.

### 4.12 Functional Evaluation of the Contribution of CO

To distinguish the contribution of P4CO‐derived CO from the overall sonodynamic ROS response, hemoglobin (Hb) was used as a CO‐binding control. Cells were treated with PBS, Hb + US, TcEM@Ce‐P4CO@M + US, or Hb + TcEM@Ce‐P4CO@M + US. Hb was present during ultrasound‐triggered activation at a final concentration of 50 µM. Intracellular CO, total ROS, mitochondrial ROS, and lipid peroxidation were subsequently evaluated using the CO‐sensitive probe, DCFH‐DA, MitoSOX Red, and C11‐BODIPY, respectively.

### 4.13 Western Blotting and Densitometric Analysis

Cells or tumor tissues were lysed in protein‐extraction buffer supplemented with protease and phosphatase inhibitors. Equal amounts of protein were separated by SDS‐PAGE, transferred onto PVDF membranes, blocked, and incubated with the indicated primary antibodies, followed by HRP‐conjugated secondary antibodies. Protein bands were visualized by enhanced chemiluminescence.

Densitometric analysis was performed using ImageJ. Non‐phosphorylated proteins were normalized to the indicated loading controls (Tubulin, GAPDH, or Na^+^/K^+^‐ATPase as appropriate), whereas phosphorylated proteins were normalized to their corresponding total proteins. Quantitative Western blot data were obtained from three independent biological experiments unless otherwise stated. Original archived Western blot images and data from three independent biological replicates are provided in the Data S1.

### 4.14 DNA Damage, cGAS–STING Signaling, ICD, and BMDC Maturation

Mitochondrial permeability transition was evaluated using the calcein‐AM/CoCl_2_ assay. DNA damage was assessed 12 h after treatment by 8‐OHdG and γ‐H2AX immunofluorescence, γ‐H2AX Western blotting, and comet assay.

STING‐associated signaling was evaluated by Western blotting for p‐STING/STING, p‐TBK1/TBK1, p‐p65/p65, and p‐IRF3/IRF3. IFN‐β secretion was quantified by ELISA.

ICD‐associated responses were evaluated by CRT exposure, intracellular and extracellular HMGB1, extracellular ATP, and HSP90 exposure. CRT and HMGB1 were additionally analyzed by Western blotting, and CRT‐positive cells were quantified by flow cytometry.

For BMDC maturation assays, conditioned medium was collected from differently treated 4T1 cells 24 h after treatment, centrifuged to remove cell debris, and subsequently incubated with BMDCs for 24 h. BMDC maturation was evaluated by CD80/CD86 flow cytometry, and IL‐12, IL‐6, and IL‐1β levels were measured by ELISA.

### 4.15 CD8^+^ T‐Cell Activation Assay

CD8^+^ T cells were isolated from mouse spleens by magnetic separation and co‐cultured with the indicated 4T1 cells at a T‐cell‐to‐tumor‐cell ratio of 5:1. Five groups were evaluated: (G1) CD8^+^ T cells alone; (G2) CD8^+^ T cells + 4T1‐WT; (G3) CD8^+^ T cells + 4T1‐CD86; (G4) CD8^+^ T cells + 4T1‐anti‐TIM‐3; and (G5) CD8^+^ T cells + dual‐engineered 4T1‐CD86/anti‐TIM‐3.

After 24 h, CD8^+^ T‐cell activation was evaluated by flow cytometric analysis of CD69 and CD25. Intracellular Granzyme B was measured after fixation/permeabilization following treatment with a protein‐transport inhibitor. IFN‐γ, TNF‐α, and IL‐12 concentrations in the co‐culture supernatants were determined by ELISA.

### 4.16 Animals and Ethical Approval

Female BALB/c mice (6–8 weeks old) were obtained from the Laboratory Animal Center of Xiamen University (Xiamen, China) and housed under specific pathogen‐free conditions with free access to food and water. All animal experiments were performed in accordance with institutional guidelines and were approved by the Xiamen University Ethics Committee (XMULAC20230200). Isoflurane anesthesia was used for procedures requiring anesthesia. At the experimental endpoint, mice were euthanized by CO_2_ inhalation before tissue collection.

### 4.17 In Vivo Biodistribution

Cy5.5‐labeled P4CO, Ce‐P4CO@M, and TcEM@Ce‐P4CO@M were intravenously administered to 4T1 tumor‐bearing mice in a total injection volume of 100 µL at an equivalent P4CO dose of 2 mg kg^−1^. Initial fluorescence signals were normalized before injection. Whole‐body fluorescence imaging was performed at 1, 3, 6, 12, 24, 48, and 72 h post‐injection. Tumors and major organs were collected for ex vivo fluorescence imaging where indicated.

### 4.18 Bilateral Tumor and Experimental Lung Metastasis Models

For the distant‐tumor challenge model, 4T1 cells (5 × 10^5^ cells in 100 µL PBS) were inoculated subcutaneously into the right flank. When primary tumors reached approximately 80–100 mm^3^, mice were randomized into six groups (*n* = 5): PBS, US, MSA‐2, P4CO + US, Ce‐P4CO@M + US, and TcEM@Ce‐P4CO@M + US. Formulations were intravenously administered in a total injection volume of 100 µL at equivalent doses of P4CO (2 mg kg^−1^) and/or MSA‐2 (1 mg kg^−1^). Ultrasound irradiation (1.0 MHz, 1.5 W cm^−2^, 50% duty cycle, 5 min) was applied only to the primary tumor 12 h after administration. A distant tumor was subsequently established on the contralateral flank using 1 × 10^5^ 4T1 cells. Tumor growth and body weight were monitored every 2 days.

For the experimental lung‐metastasis model, mice bearing primary 4T1 tumors were randomized into six treatment groups (*n* = 5 mice per group) and received 1 × 10^5^ 4T1‐Luc cells intravenously. Treatments were administered on days 3, 7, 11, and 18, followed by local ultrasound irradiation of the primary tumor. Lung metastatic burden was monitored by bioluminescence imaging after D‐luciferin administration (150 mg kg^−1^), and lungs were collected at endpoint for gross and histological analyses.

### 4.19 In Vivo Immune Profiling and Tissue Analysis

Tumors and tumor‐draining lymph nodes were processed into single‐cell suspensions by mechanical and enzymatic dissociation followed by filtration through a 70‐µm cell strainer. After red blood cell lysis and Fc blocking, cells were stained with the indicated antibodies. Following exclusion of debris and doublets, CD45^+^ immune cells were selected. CD4^+^ and CD8^+^ T cells were identified within CD3^+^ cells; PD‐1^+^TIM‐3^+^ cells within the CD8^+^ T‐cell population were quantified as an exhaustion‐associated phenotype; Tregs were defined as CD25^+^Foxp3^+^ cells within the CD4^+^ population. Tumor‐associated macrophages were identified as CD11b^+^F4/80^+^ cells and further classified as CD86^+^ M1‐like or CD206^+^ M2‐like populations. Mature DCs in tumor‐draining lymph nodes were identified as CD80^+^CD86^+^ cells within CD11c^+^ DCs. Data were analyzed using FlowJo.

Tumor and lung tissues were fixed in 4% paraformaldehyde and processed for H&E, immunohistochemistry, or immunofluorescence staining. GPX4, γ‐H2AX, CRT, HMGB1, and p‐STING were evaluated in tumor tissues. HIF‐1α expression in tumor lysates was additionally determined by Western blotting. Serum IFN‐β, IFN‐γ, TNF‐α, IL‐6, IL‐12, and IL‐10 were quantified by ELISA.

### 4.20 RNA Sequencing and Bioinformatic Analysis

Total RNA was extracted from tumor tissues in the PBS and TcEM@Ce‐P4CO@M + US groups and subjected to high‐throughput RNA sequencing (RNA‐seq). Data were analyzed using principal component analysis (PCA) and volcano plots to identify differentially expressed genes (DEGs). Functional enrichment analyses were performed utilizing the Kyoto Encyclopedia of Genes and Genomes (KEGG) and Gene Ontology (GO) databases. A targeted heatmap was generated to highlight genes related to ferroptosis, STING signaling, and T‐cell activation. Pathway‐level enrichment was further evaluated using Gene Set Enrichment Analysis (GSEA).

### 4.21 Statistical Analysis

Data are presented as mean ± SD, and the number of independent biological replicates, independent measurements, or animals is specified in the corresponding figure legends, as appropriate. Statistical analyses were performed using GraphPad Prism version 10 (GraphPad Software, Boston, MA, USA). Comparisons between two independent groups were performed using a two‐tailed unpaired Student's t‐test. Comparisons among three or more independent groups were performed using one‐way analysis of variance (ANOVA) followed by Tukey's multiple‐comparisons test. For experiments involving two experimental factors, such as treatment and oxygen condition, two‐way ANOVA followed by Tukey's multiple‐comparisons test was used. Longitudinal measurements repeatedly obtained from the same animals, including tumor‐associated fluorescence and tumor‐growth data, were analyzed using two‐way repeated‐measures ANOVA followed by Šídák's multiple‐comparisons test. RNA‐seq data were analyzed as described in Section 4.20, including differential‐expression and enrichment analyses with appropriate multiple‐testing correction. Exact group comparisons represented by significance brackets are indicated directly in the corresponding figures. A value of p < 0.05 was considered statistically significant (^*^ *p* < 0.05, ^**^ *p* < 0.01, ^***^ *p* < 0.001, and ^****^ *p* < 0.0001).

## Author Contributions

**Mengli Ma**: conceptualization, investigation, writing – review and editing, writing – original draft, methodology. **Yingying Wu**: conceptualization, methodology. **Yunjie Wang**: conceptualization, investigation. **Yang Li**: conceptualization, methodology. **Yijia Zhang**: formal analysis, validation. **Zhibo Zhang**: investigation, resources, supervision. **Jinzhi Peng**: visualization, project administration. **Xiangyu Wu**: conceptualization, investigation. **Yi Luo**: investigation, validation. **Weijing Zhang**: investigation, methodology, validation. **Xin Ye**: investigation, conceptualization. **Weisong Lai**: methodology, conceptualization, software. **Shengyu Wang**: funding acquisition. **Dan Du**: investigation, validation, methodology. **Wengang Li**: funding acquisition, visualization. **Nan Zheng**: methodology, formal analysis, resources. **Fanghong Luo**: funding acquisition, investigation, supervision. **Ting Wu**: funding acquisition, writing – review and editing, investigation, data curation.

## Conflicts of Interest

The authors declare no conflicts of interest.

## Supporting information




**Supporting File 1**: advs78187‐sup‐0001‐SuppMat.docx.

**Supporting File 2**: advs78187‐sup‐0002‐Data.pdf.

## Data Availability

The data that support the findings of this study are available from the corresponding author upon reasonable request.
