# Supplementary material for: Ultrasound‐Activated Ce(IV)‐Integrated Biomimetic Nanoplatform for Oxygenation‐Reinforced Gas‐Sonodynamic Ferroptosis and cGAS–STING‐Driven Antitumor Immunity
Source: Adv Sci (Weinh). 2026 Oct 31:e78187. Online ahead of print. doi: 10.1002/advs.78187 (PMC13723706; doi:10.1002/advs.78187)

GPX4 – Original Archived Western Blot Images from Three Independent Biological Replicates

	Replicate 1<br>Representative blot shown in Fig. 5J	Replicate 2<br>Independent biological replicate	Replicate 3<br>Independent biological replicate
GPX4 (~16 kDa)	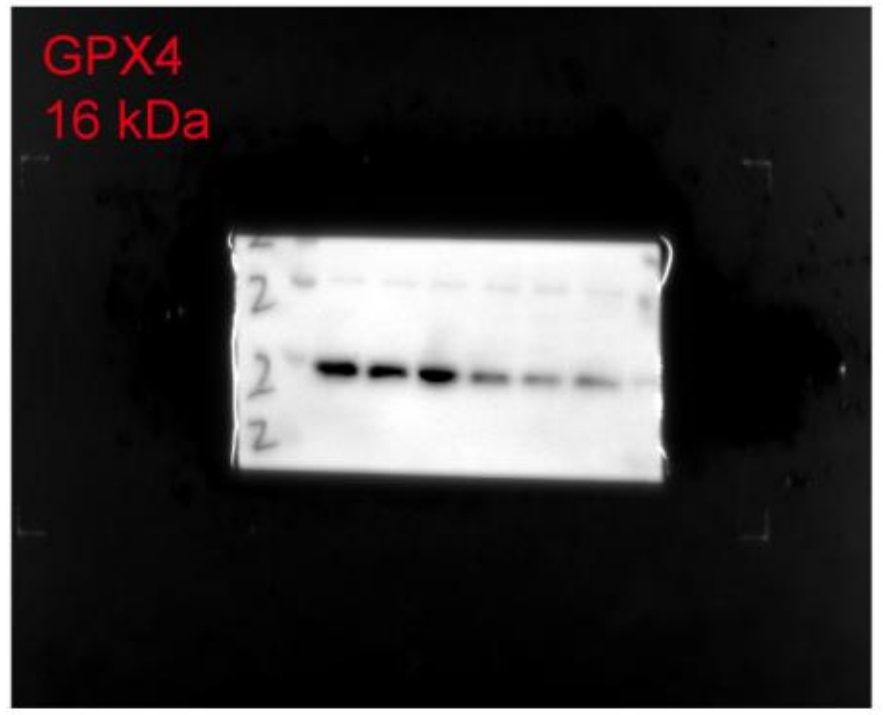	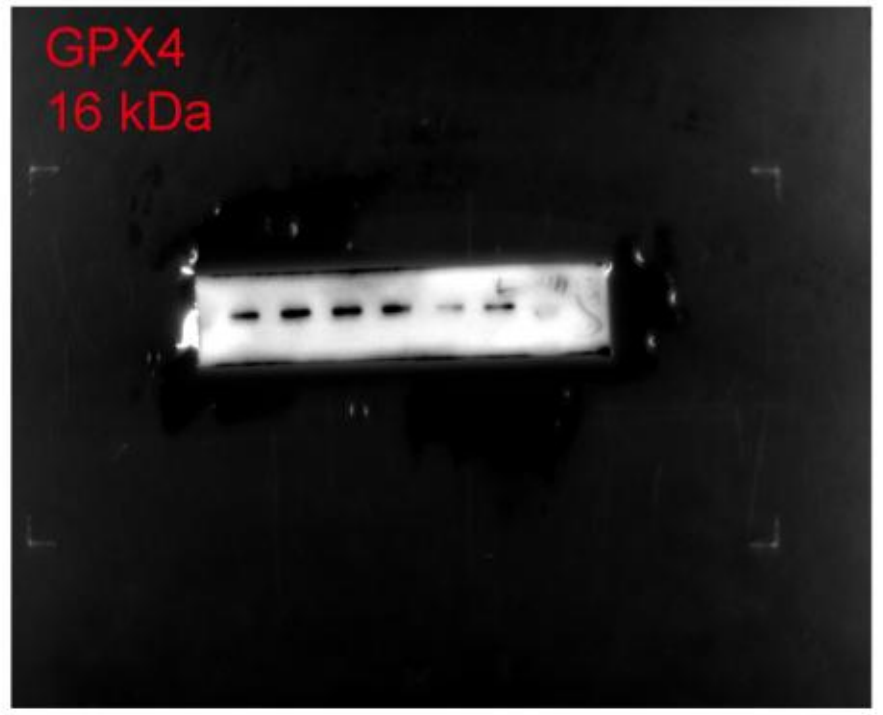	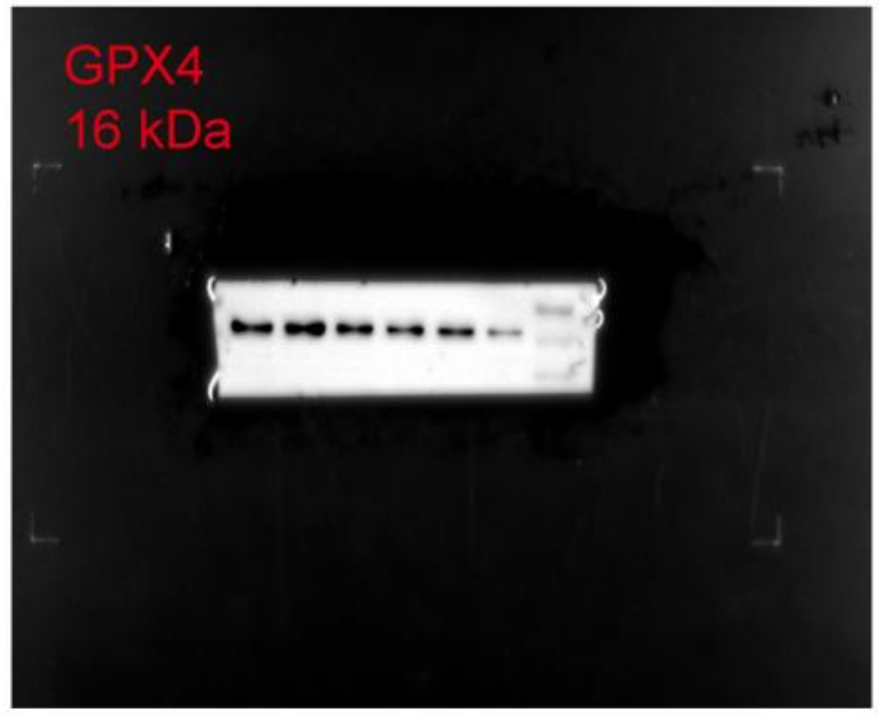
Tubulin (~56 kDa)	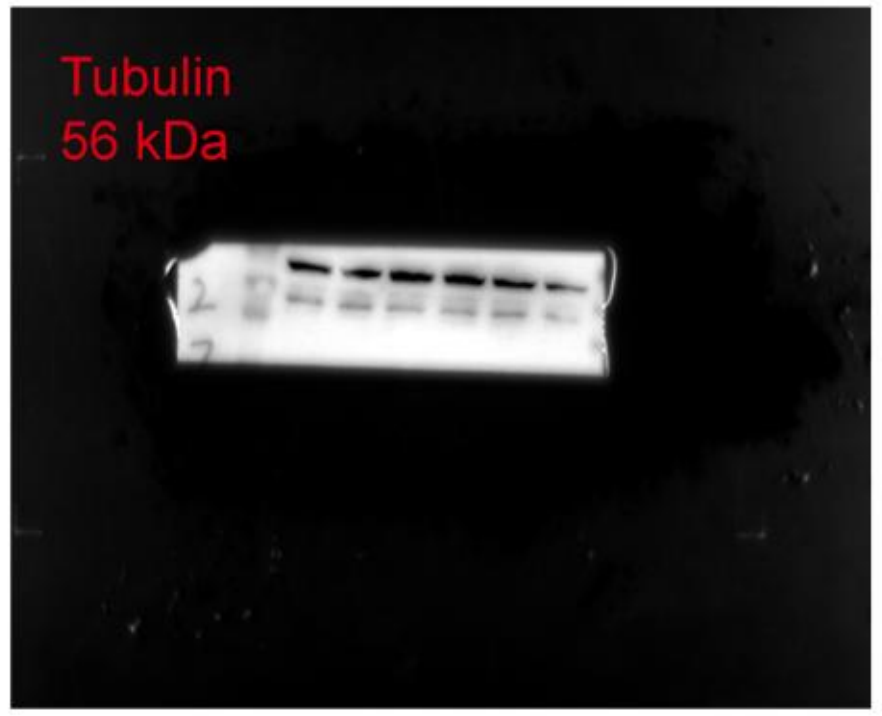	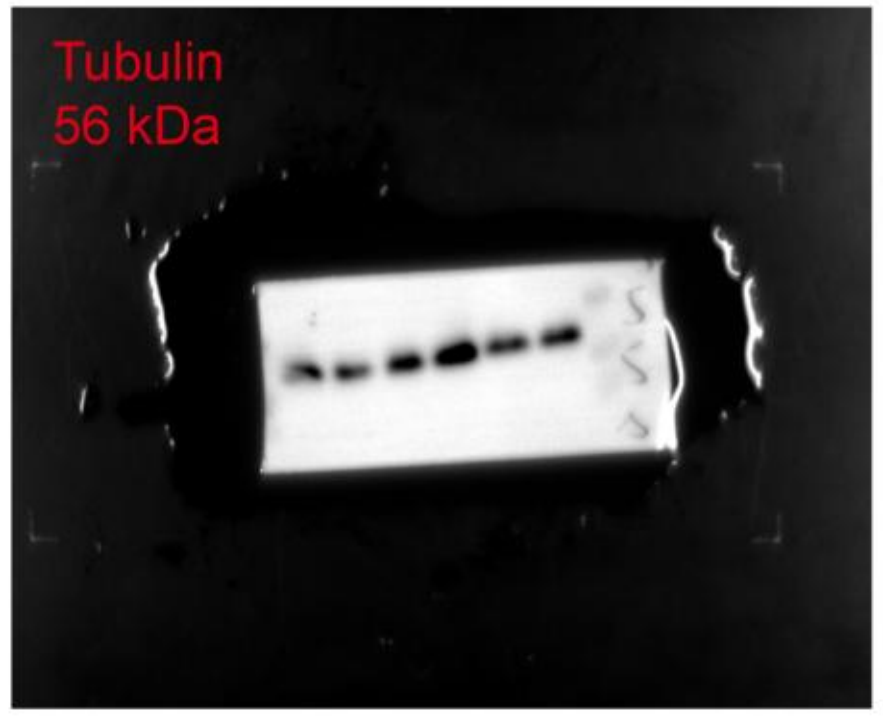	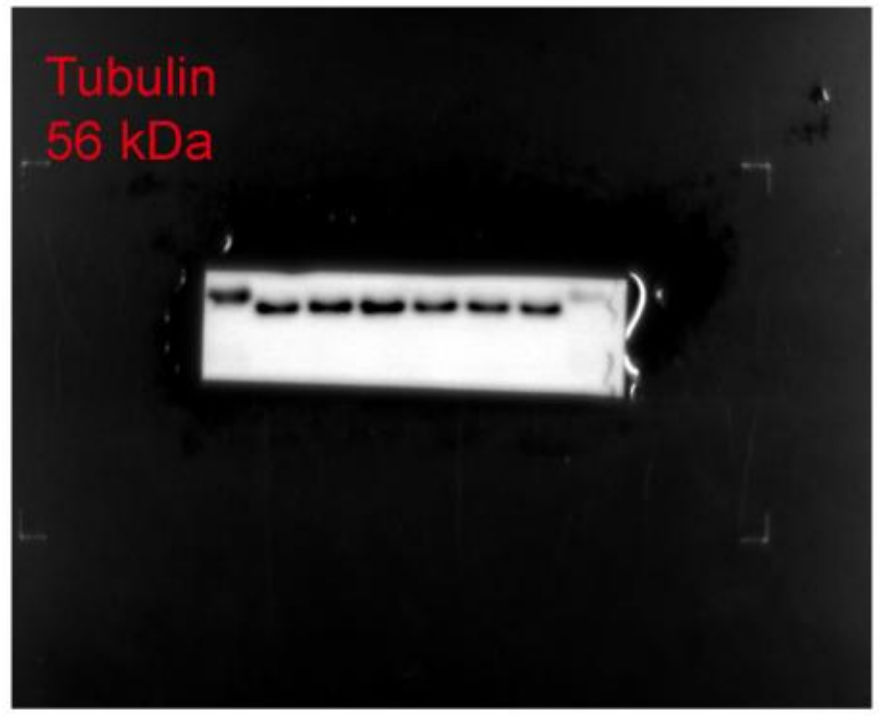

SLC7A11 – Original Archived Western Blot Images from Three Independent Biological Replicates

Replicate 1
Representative blot
shown in Fig. 5K

Replicate 2
Independent
biological replicate

Replicate 3
Independent
biological replicate

SLC7A11 (~55 kDa)

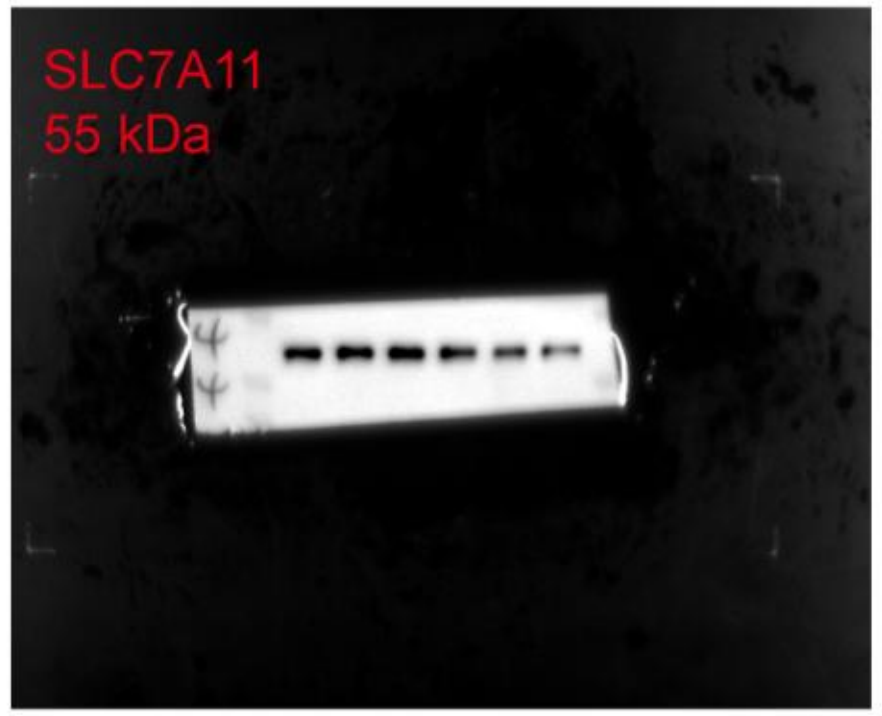

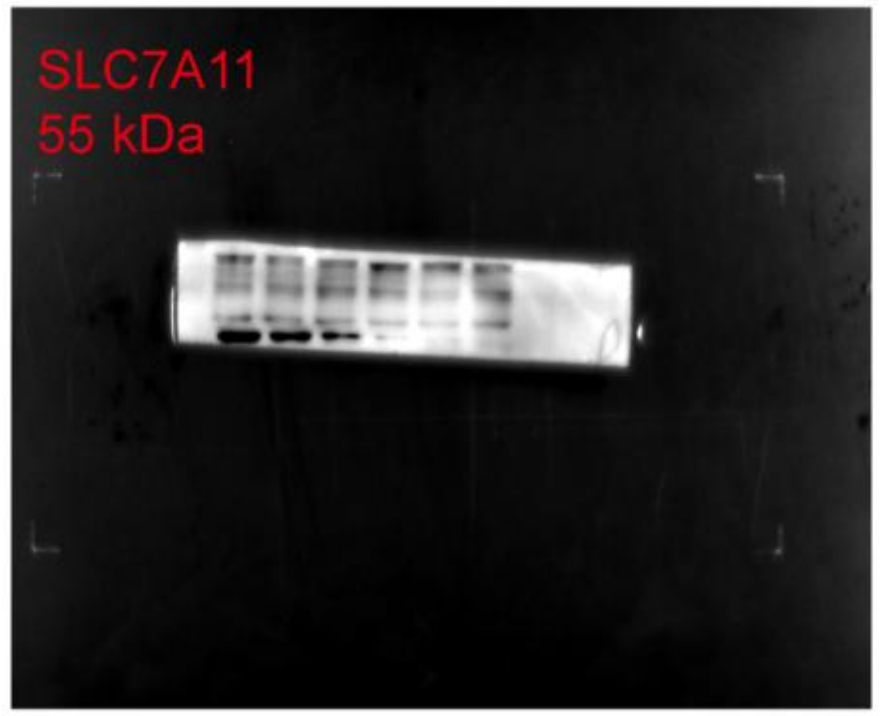

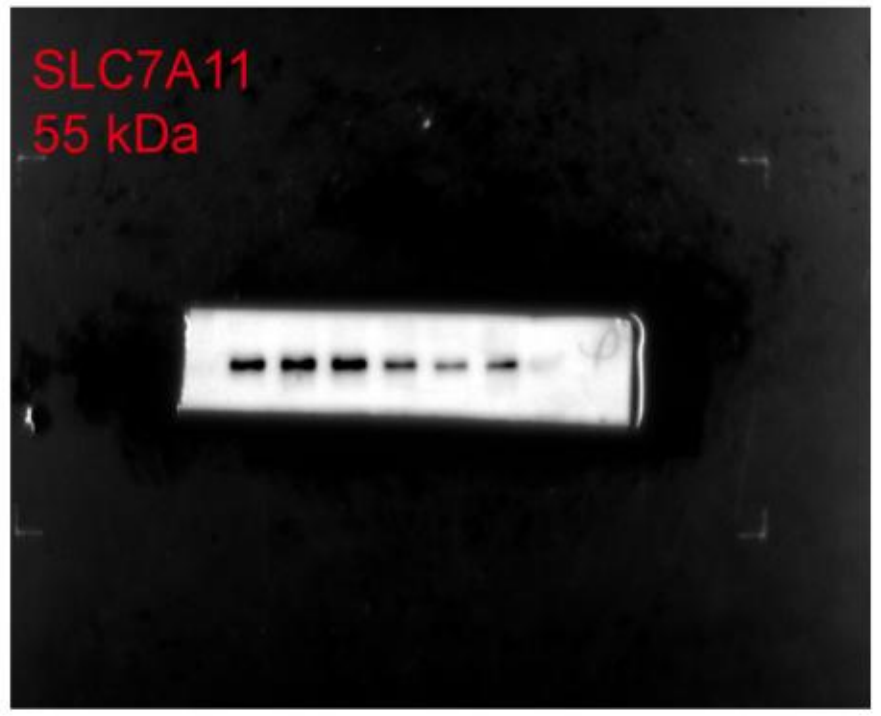

GAPDH (~37 kDa)

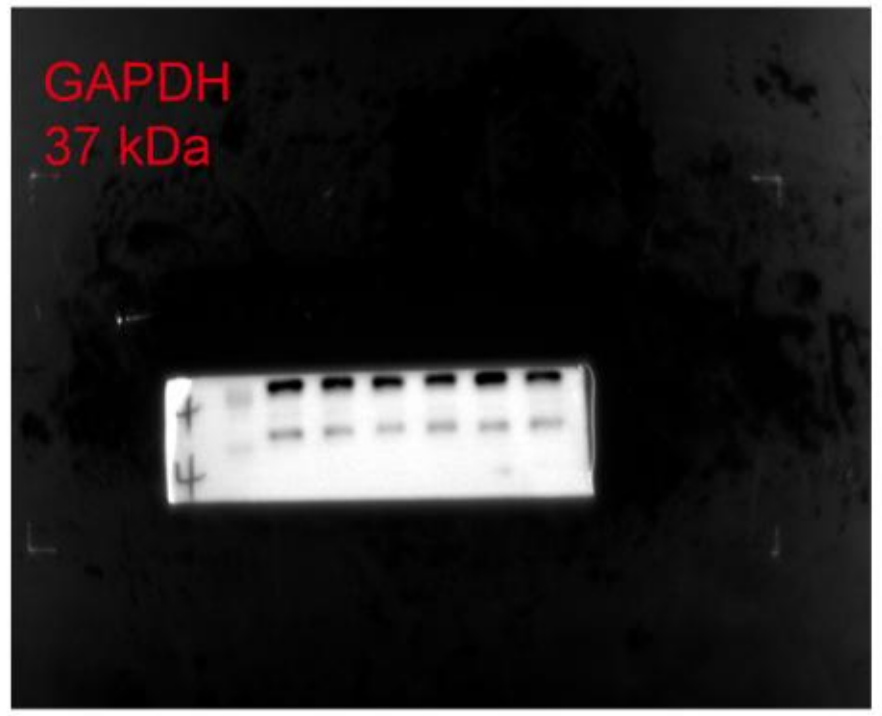

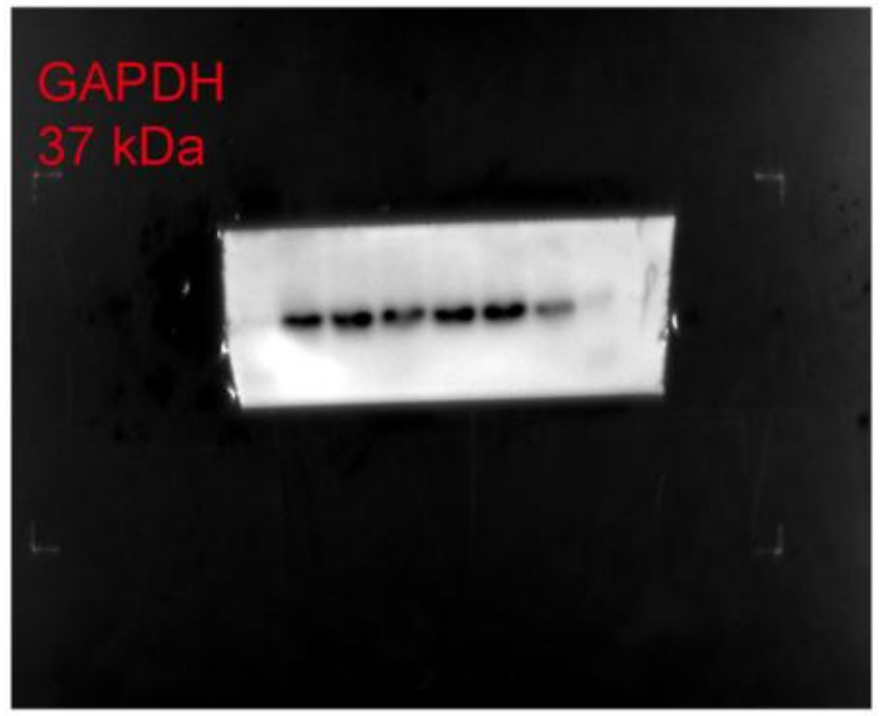

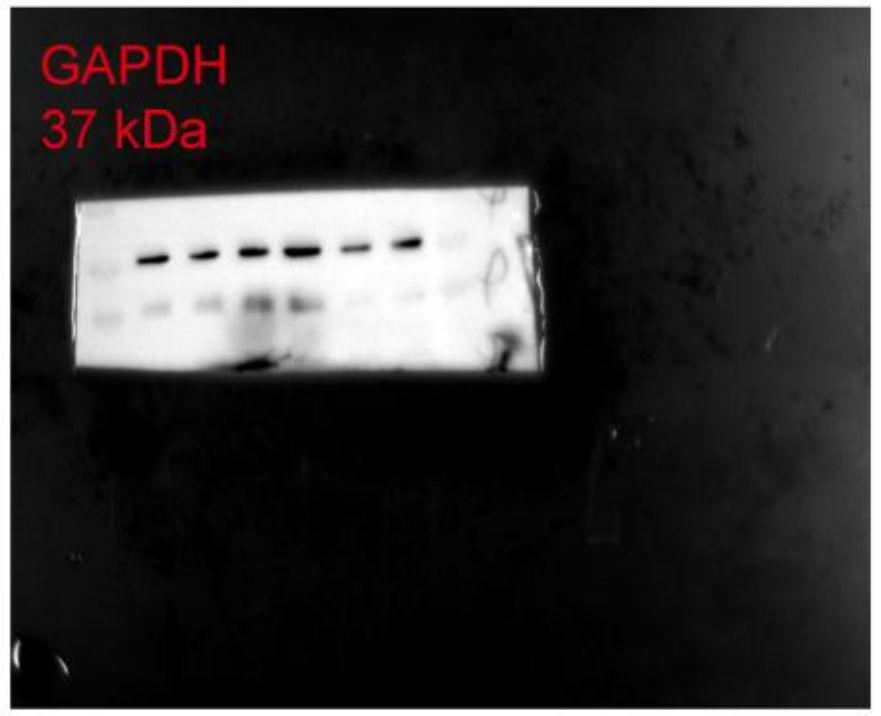

γ-H2AX– Original Archived Western Blot Images from Three Independent Biological Replicates

Replicate 1	Replicate 2	Replicate 3
Representative blot shown in Fig. 6E	Independent biological replicate	Independent biological replicate

γ-H2AX (~17 kDa)

Tubulin (~56 kDa)

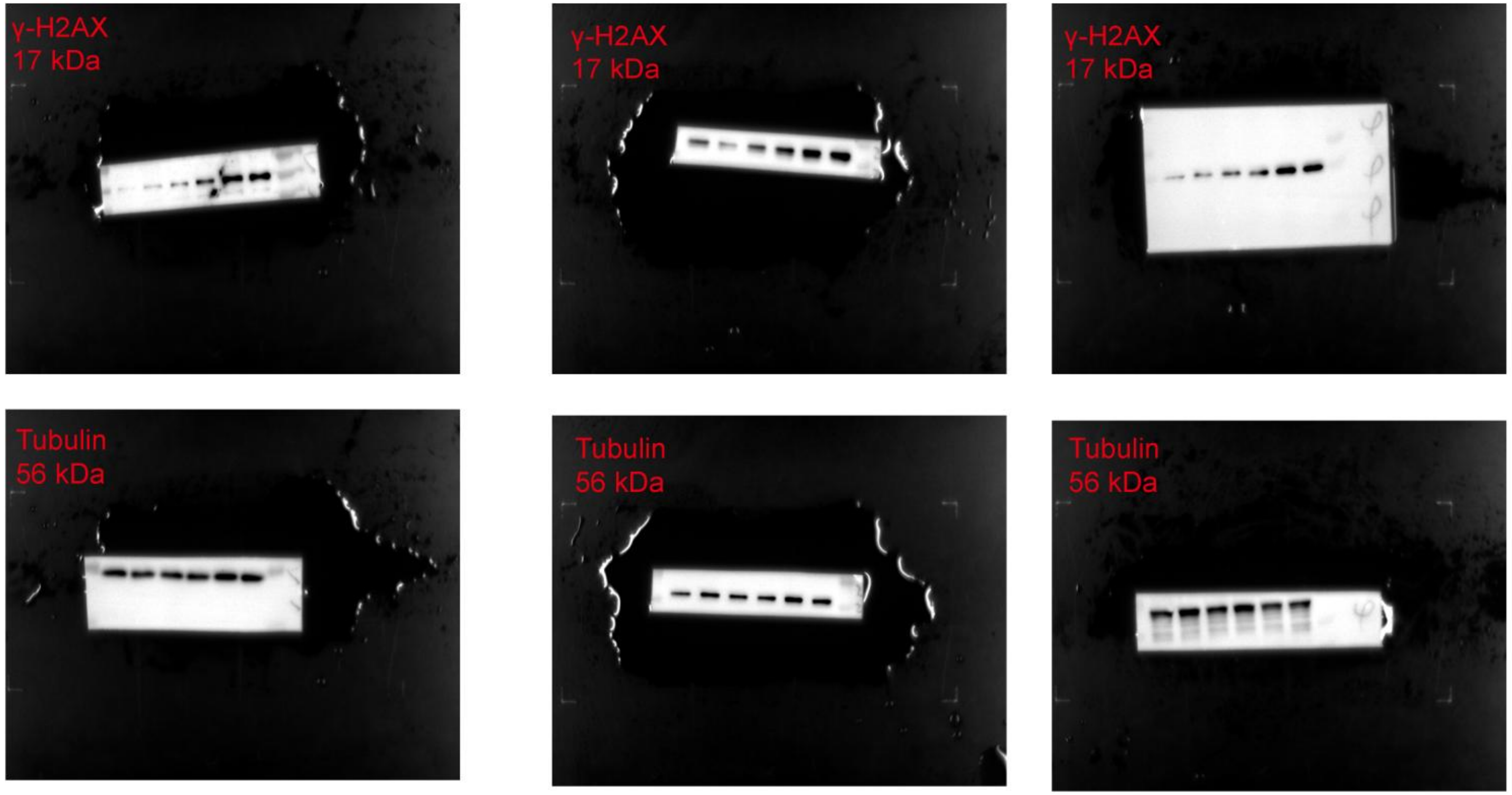

STING – Original Archived Western Blot Images from Three Independent Biological Replicates

Replicate 1 Representative blot shown in Fig. 6F	Replicate 2 Independent biological replicate	Replicate 3 Independent biological replicate

STING (~42 kDa)

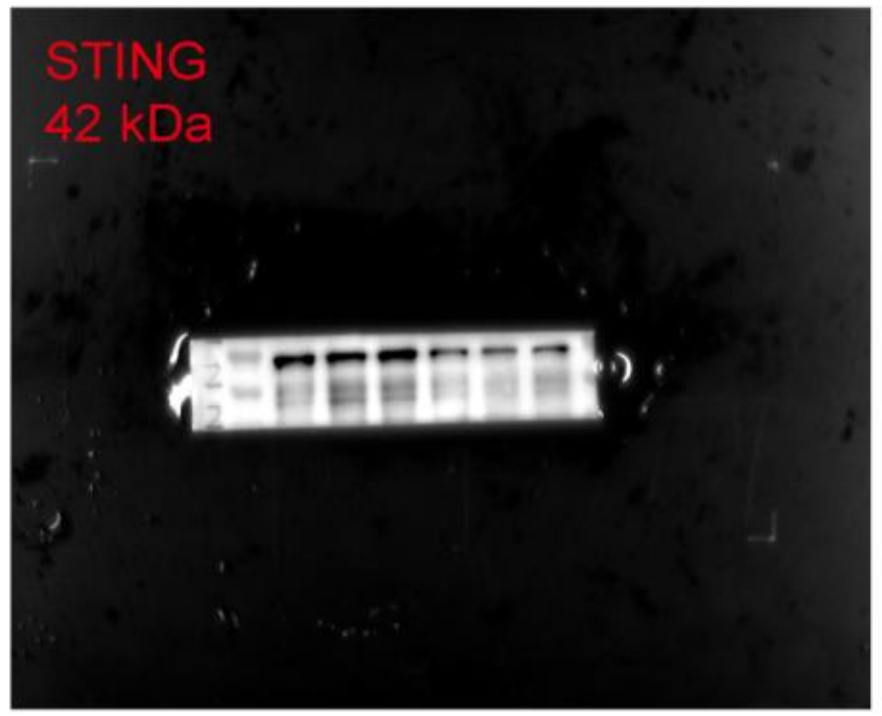

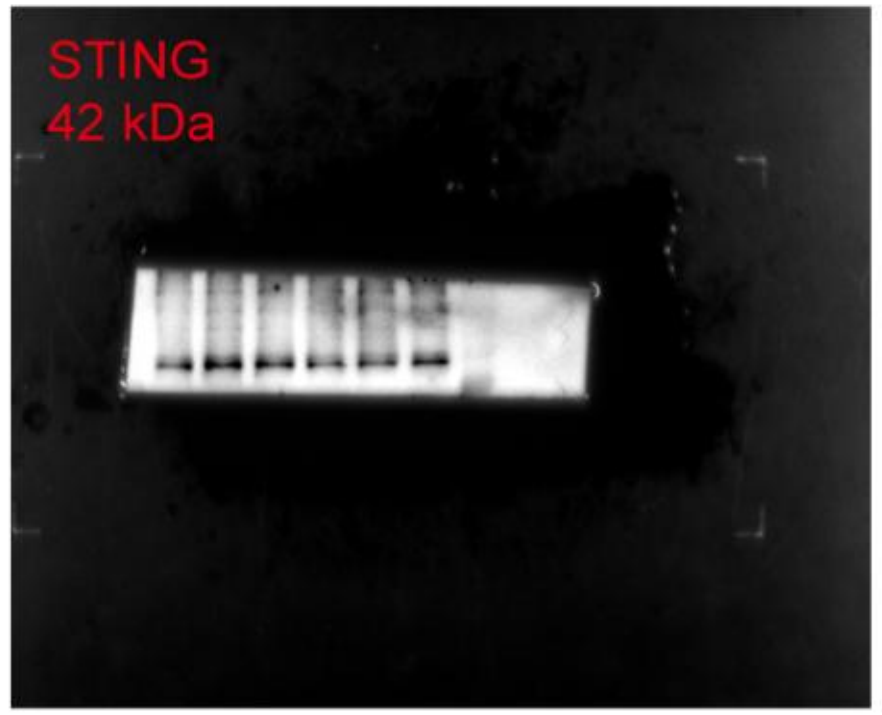

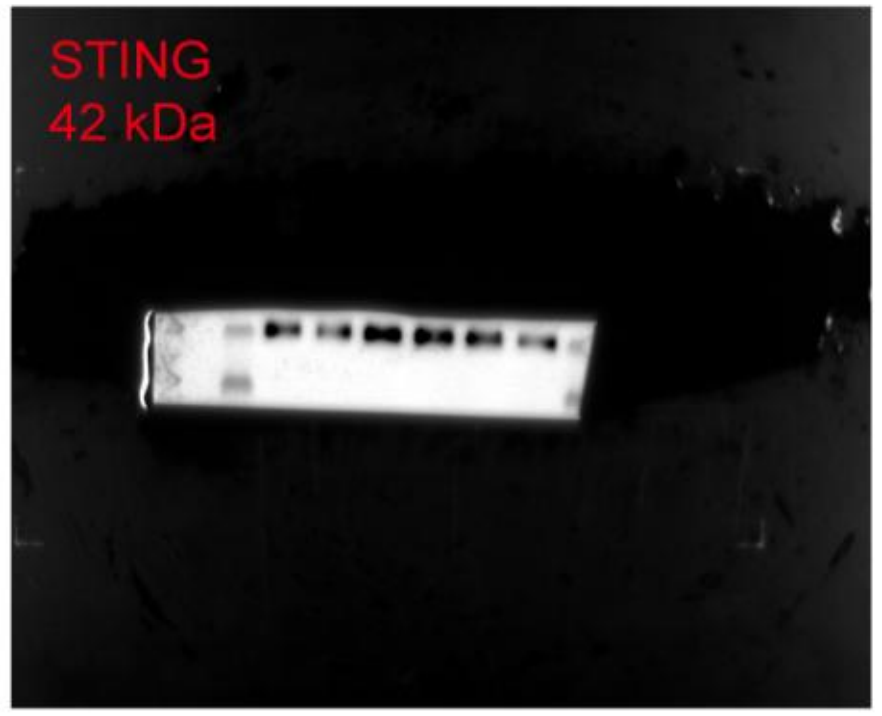

GAPDH (~37 kDa)

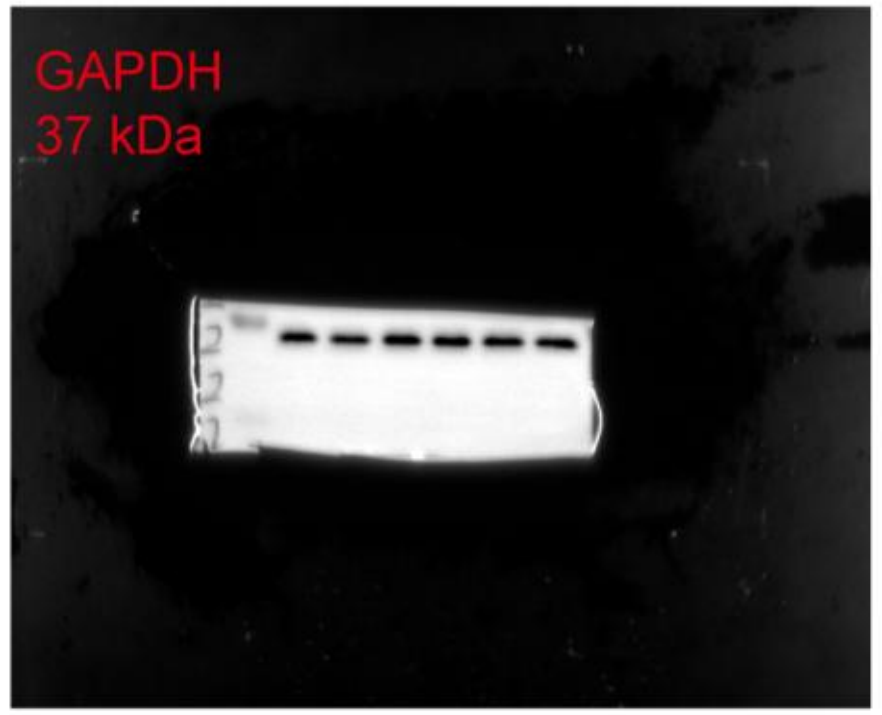

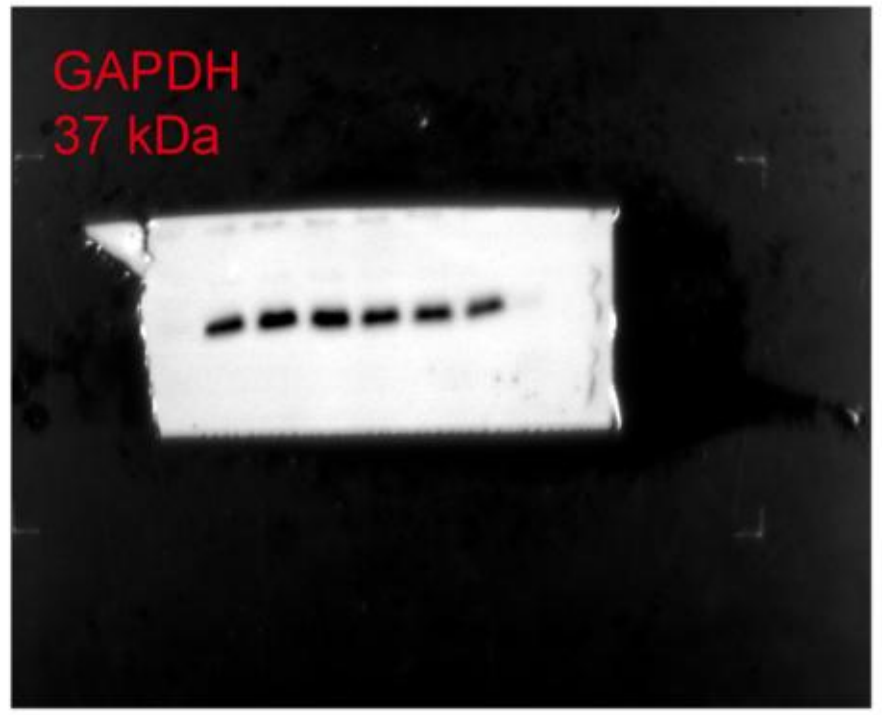

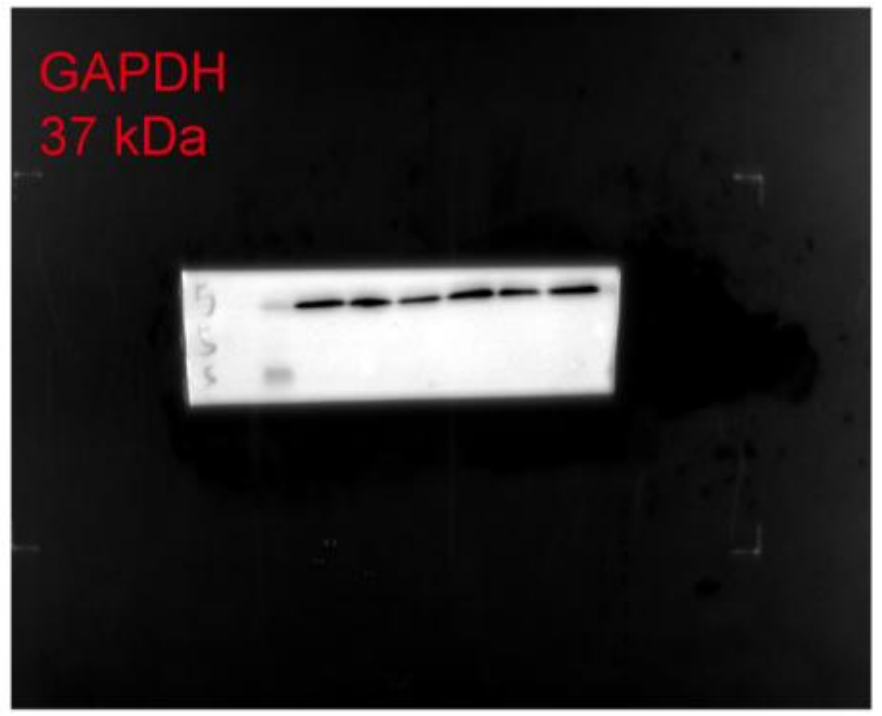

p-STING – Original Archived Western Blot Images from Three Independent Biological Replicates

	Replicate 1<br>Representative blot shown in Fig. 6F	Replicate 2<br>Independent biological replicate	Replicate 3<br>Independent biological replicate

p-STING (~42 kDa)

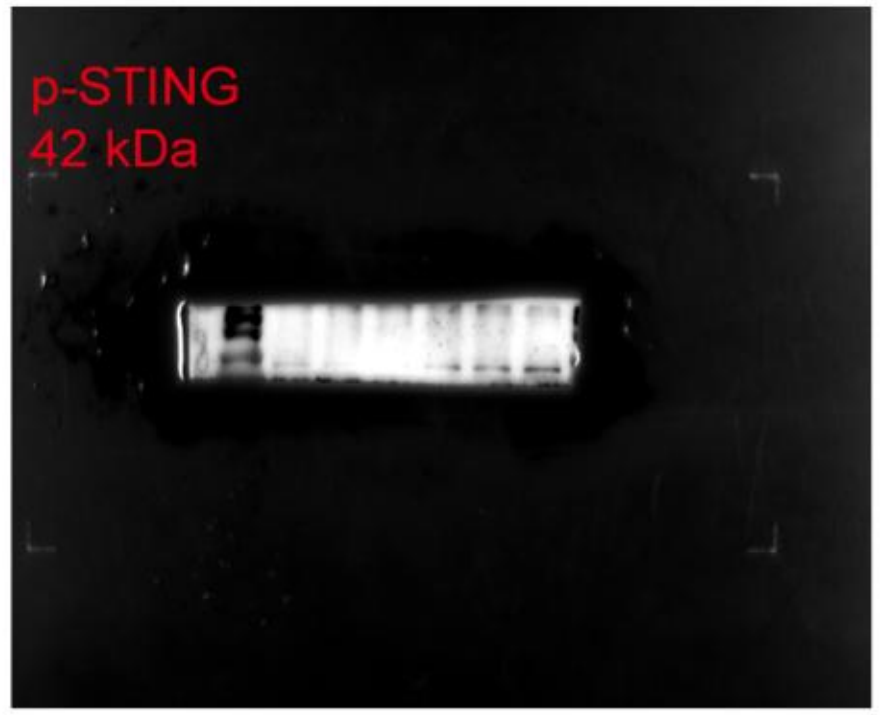

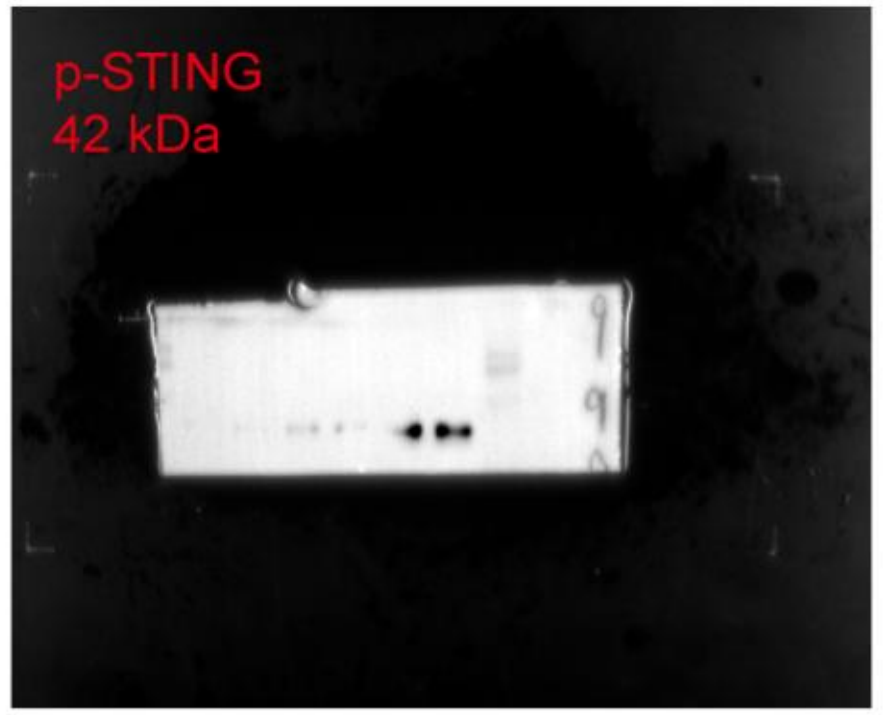

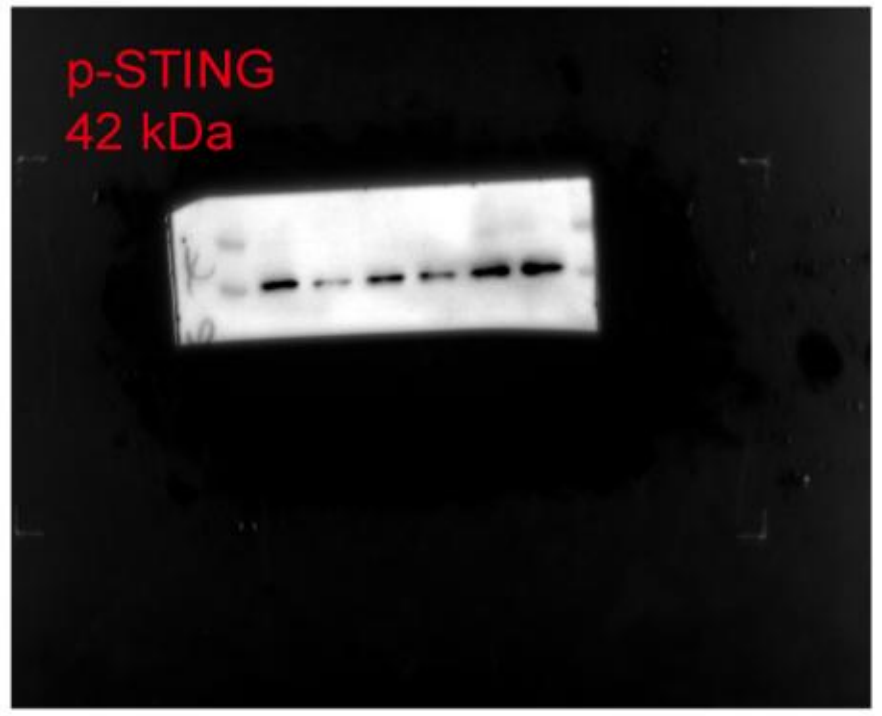

GAPDH (~37 kDa)

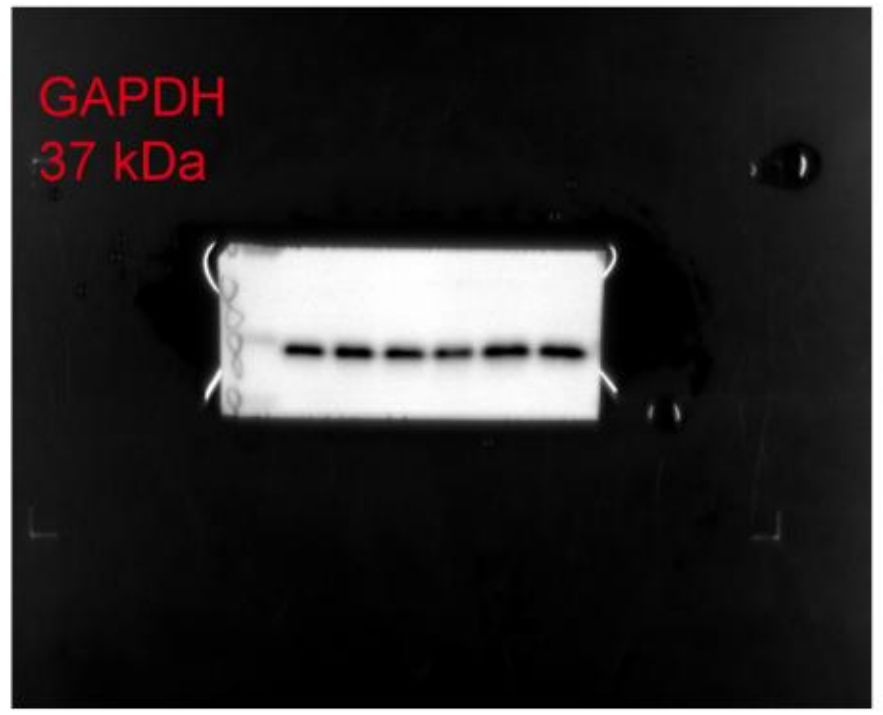

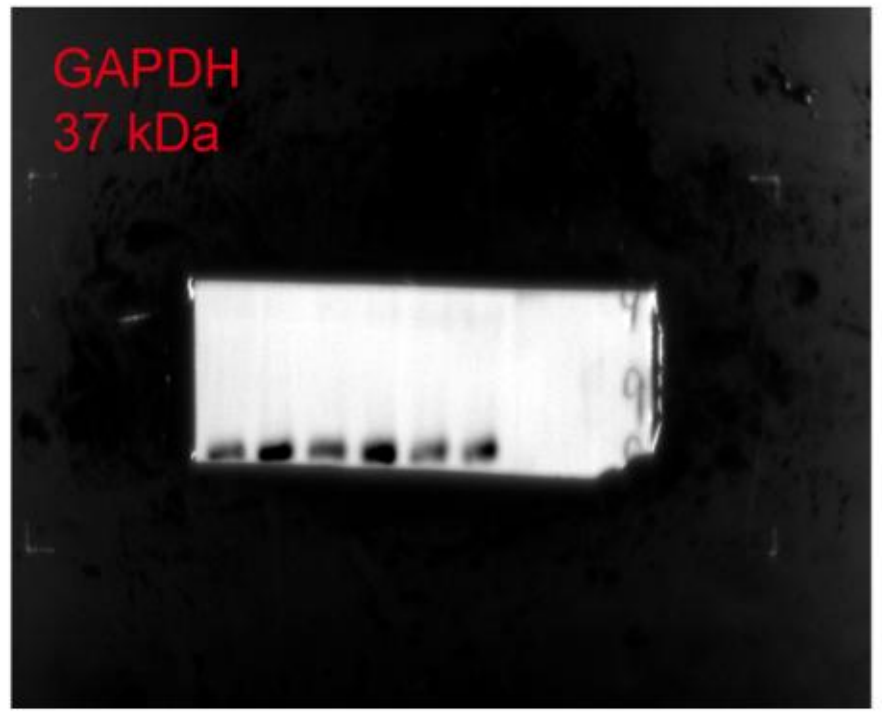

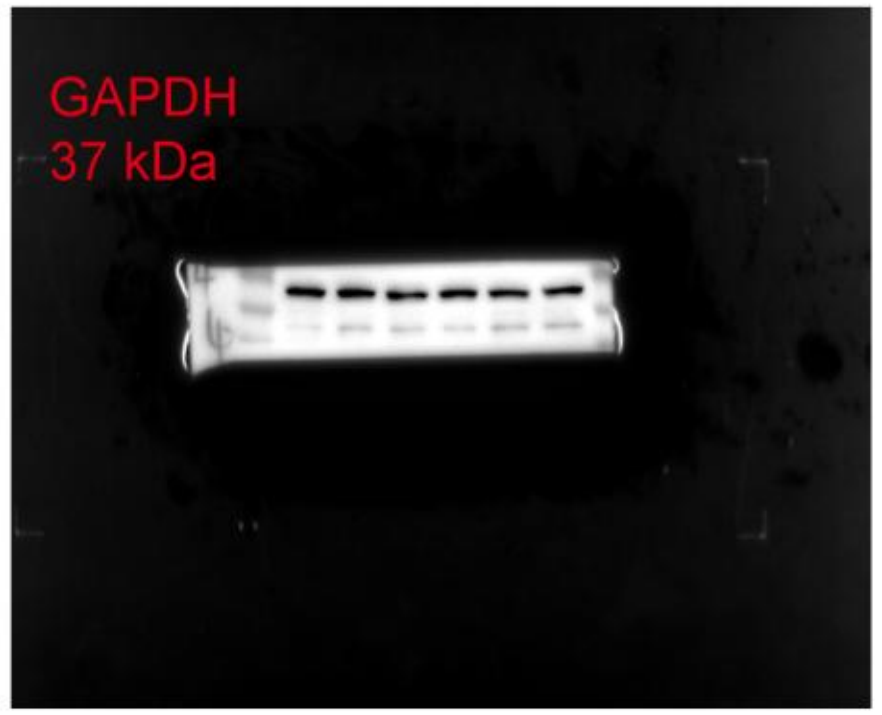

IRF3 – Original Archived Western Blot Images from Three Independent Biological Replicates

Replicate 1
Representative blot shown in Fig. 6G

Replicate 2
Independent biological replicate

Replicate 3
Independent biological replicate

IRF3 (~50 kDa)

GAPDH (~37 kDa)

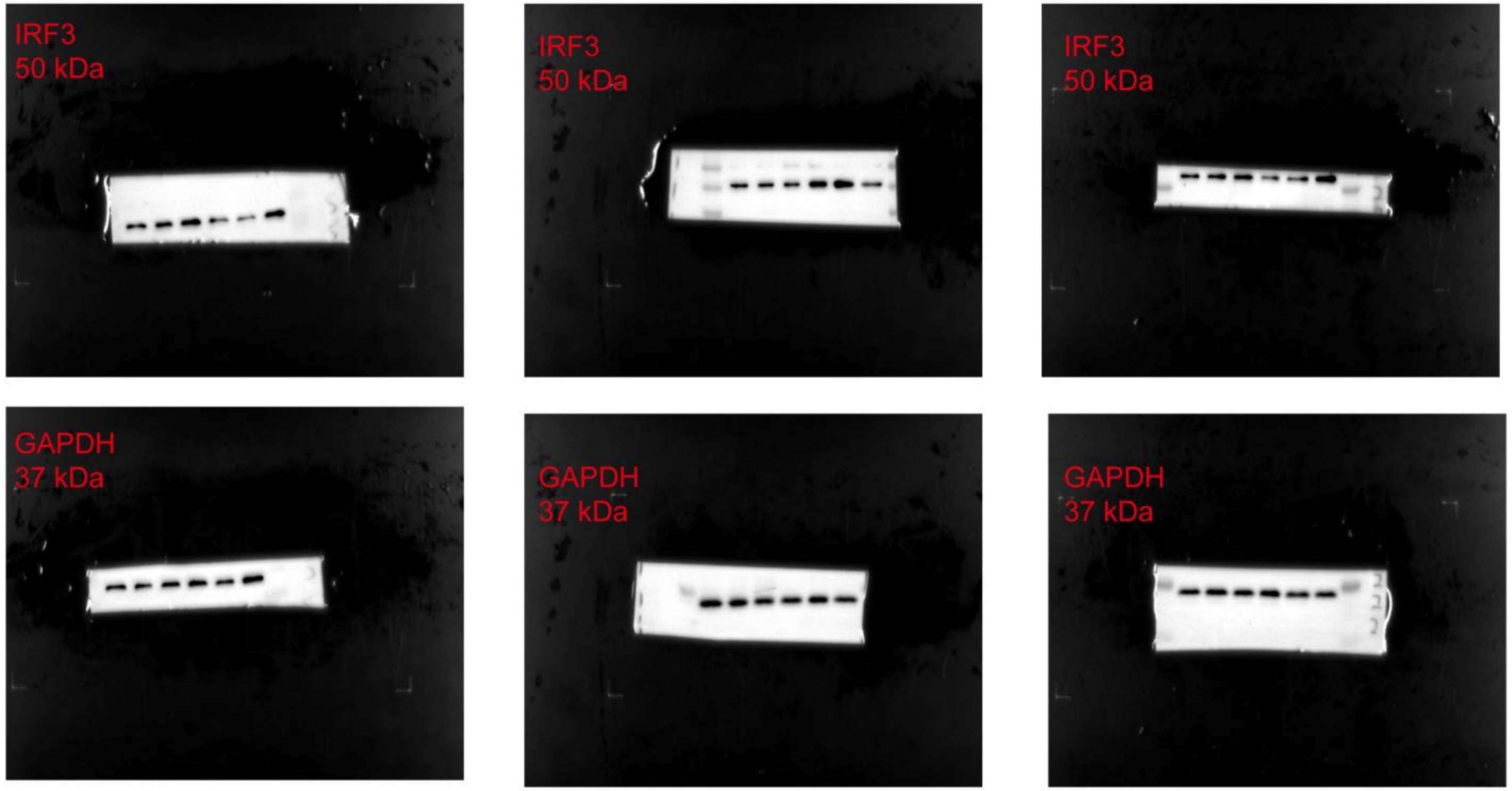

p-IRF3 – Original Archived Western Blot Images from Three Independent Biological Replicates

Replicate 1
Representative blot
shown in Fig. 6G

Replicate 2
Independent
biological replicate

Replicate 3
Independent
biological replicate

p-IRF3 (~50 kDa)

GAPDH (~37 kDa)

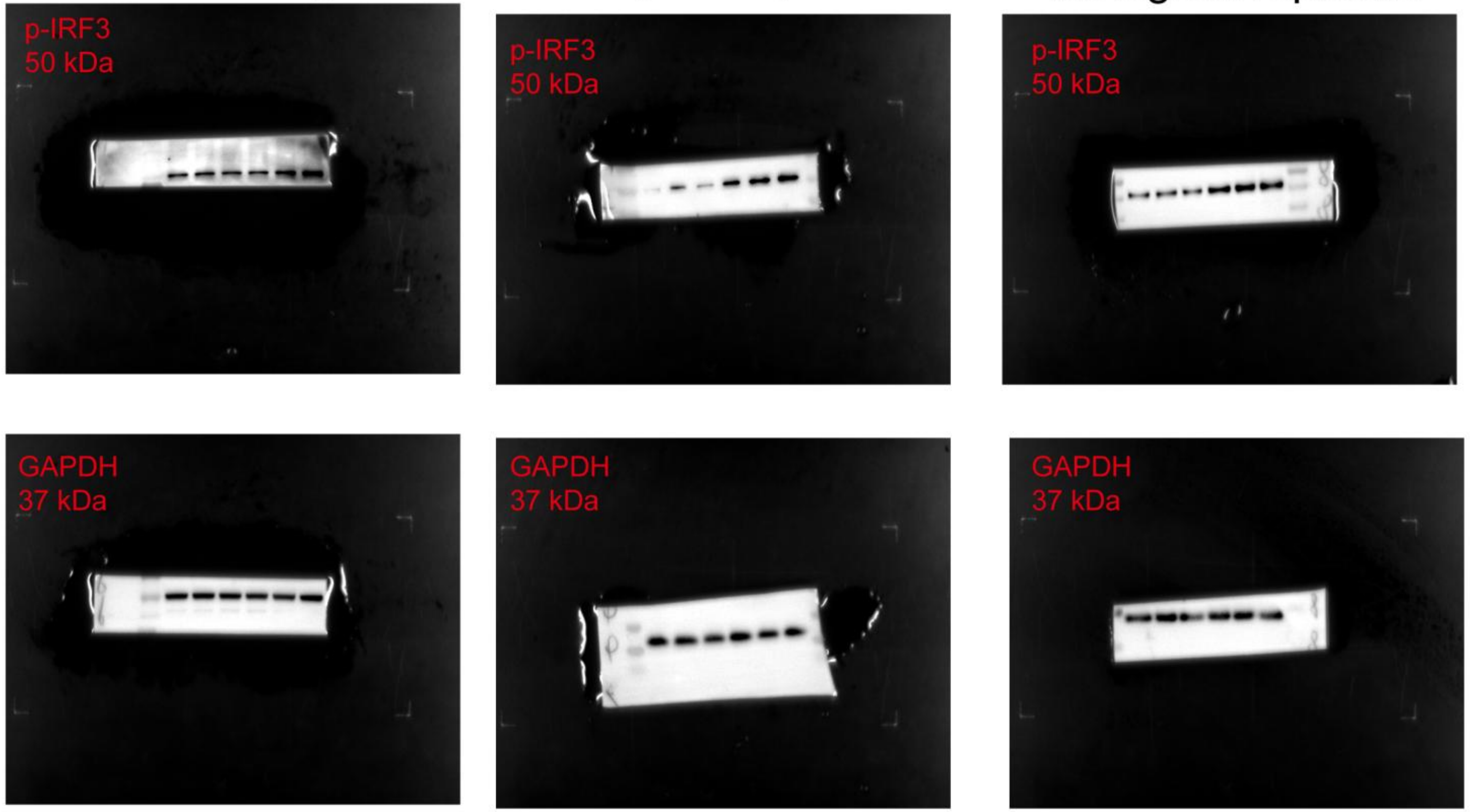

p65 – Original Archived Western Blot Images from Three Independent Biological Replicates

Replicate 1
Representative blot shown in Fig. 6G

Replicate 2
Independent biological replicate

Replicate 3
Independent biological replicate

p65 (~65 kDa)

GAPDH (~37 kDa)

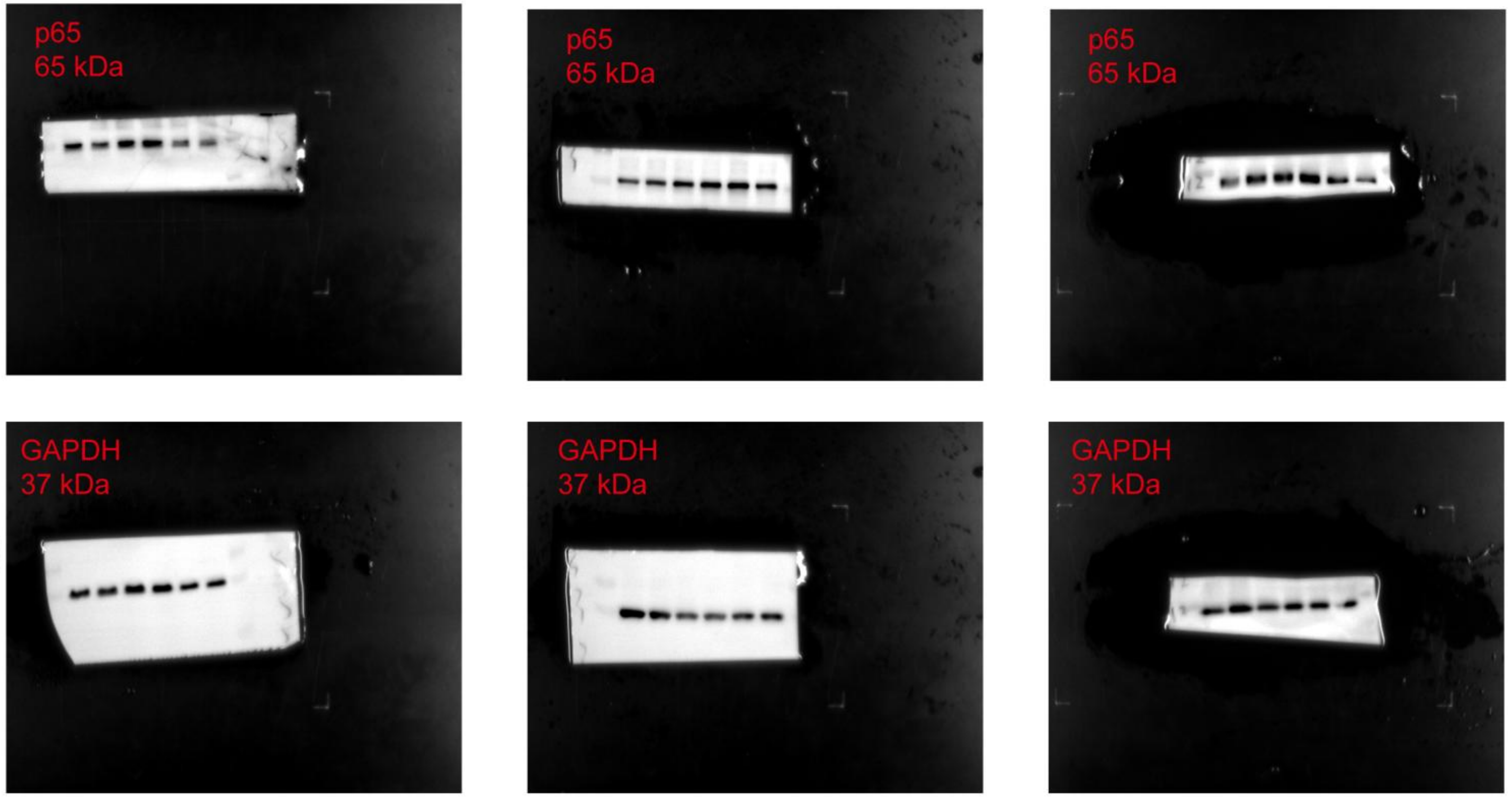

p-p65 – Original Archived Western Blot Images from Three Independent Biological Replicates

Replicate 1<br>Representative blot shown in Fig. 6G	Replicate 2<br>Independent biological replicate	Replicate 3<br>Independent biological replicate

p-p65 (~65 kDa)

GAPDH (~37 kDa)

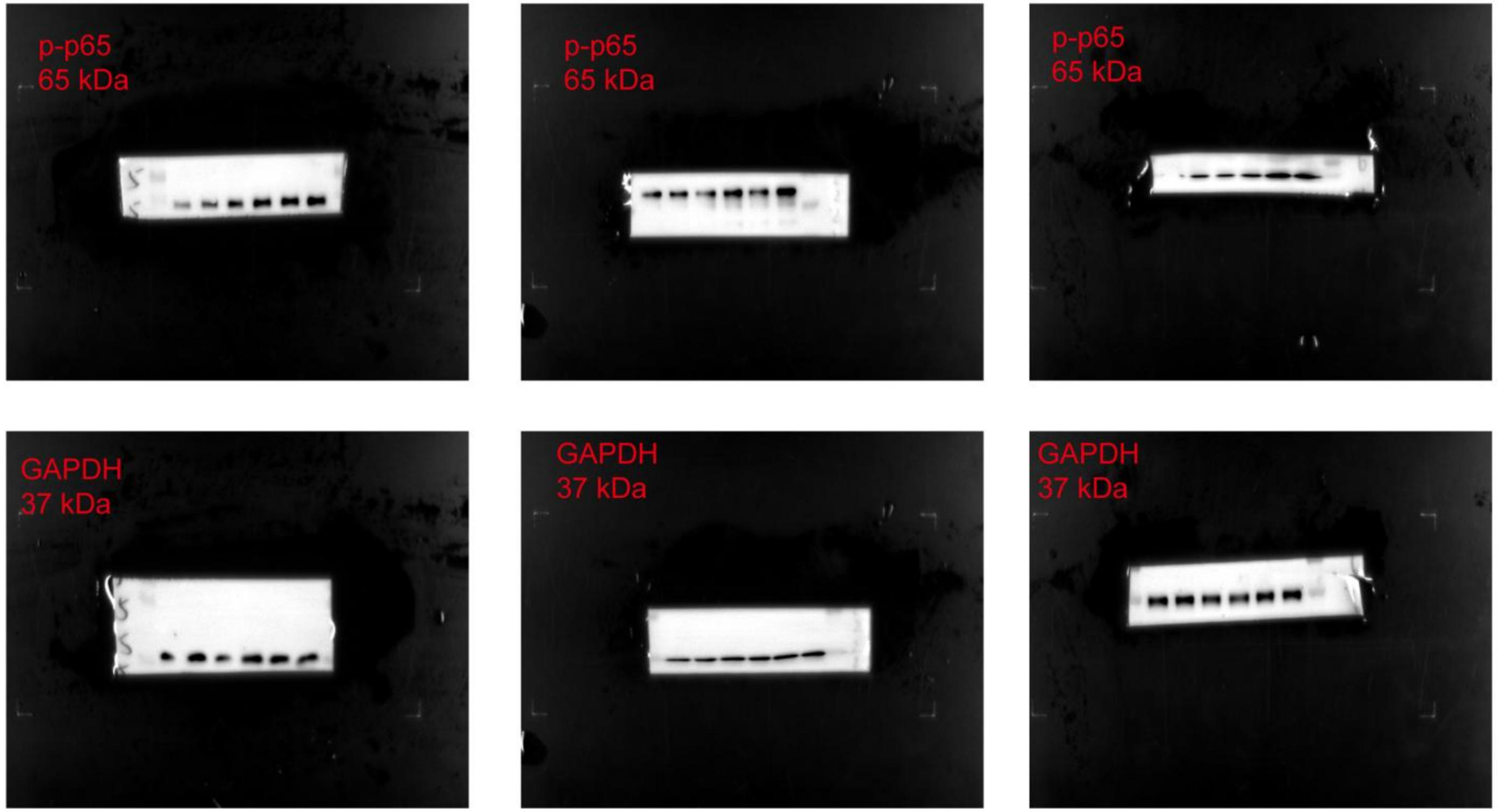

TBK1 – Original Archived Western Blot Images from Three Independent Biological Replicates

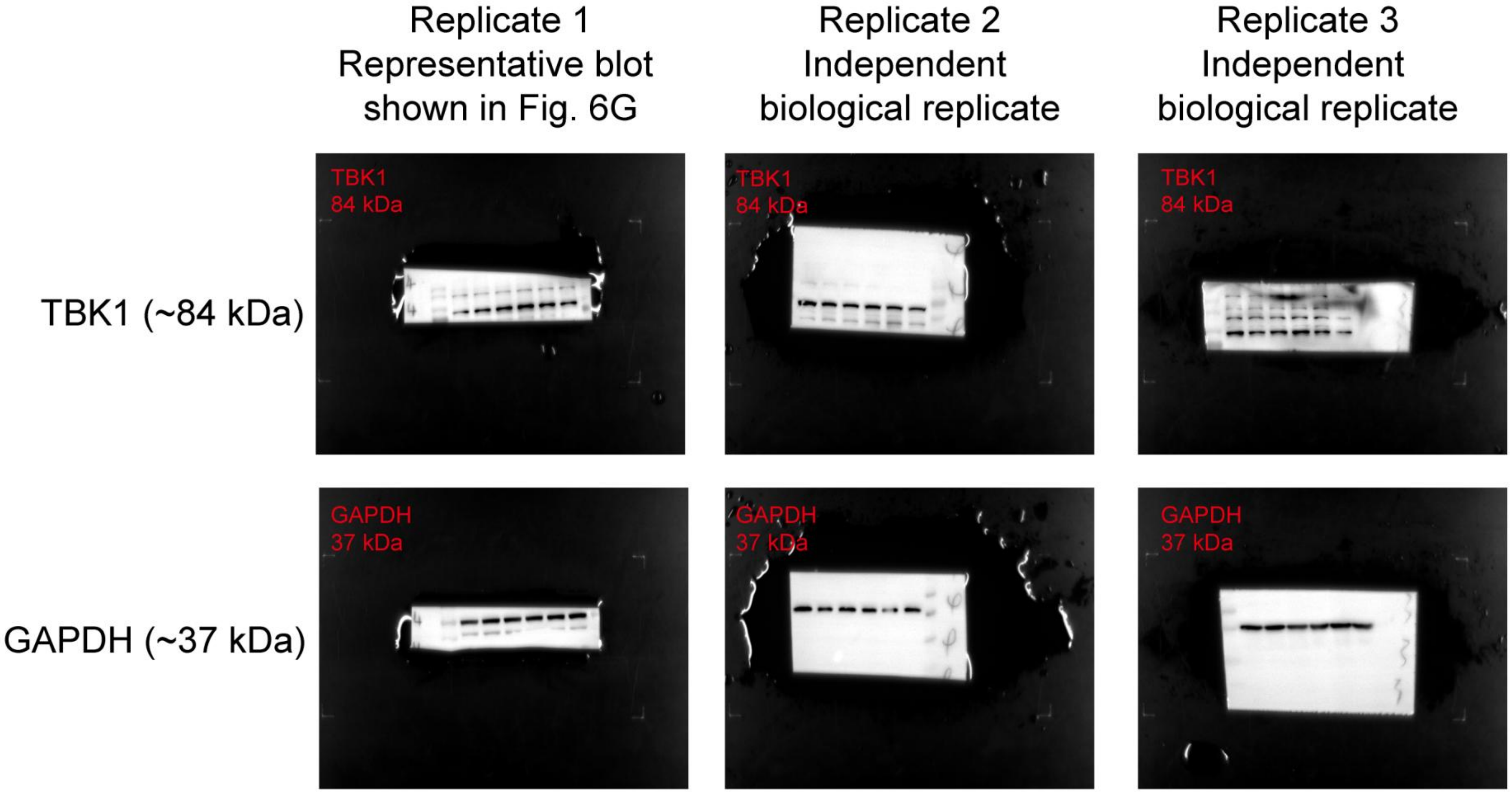

p-TBK1 – Original Archived Western Blot Images from Three Independent Biological Replicates

Replicate 1	Replicate 2	Replicate 3
Representative blot shown in Fig. 6G	Independent biological replicate	Independent biological replicate

p-TBK1 (~84 kDa)

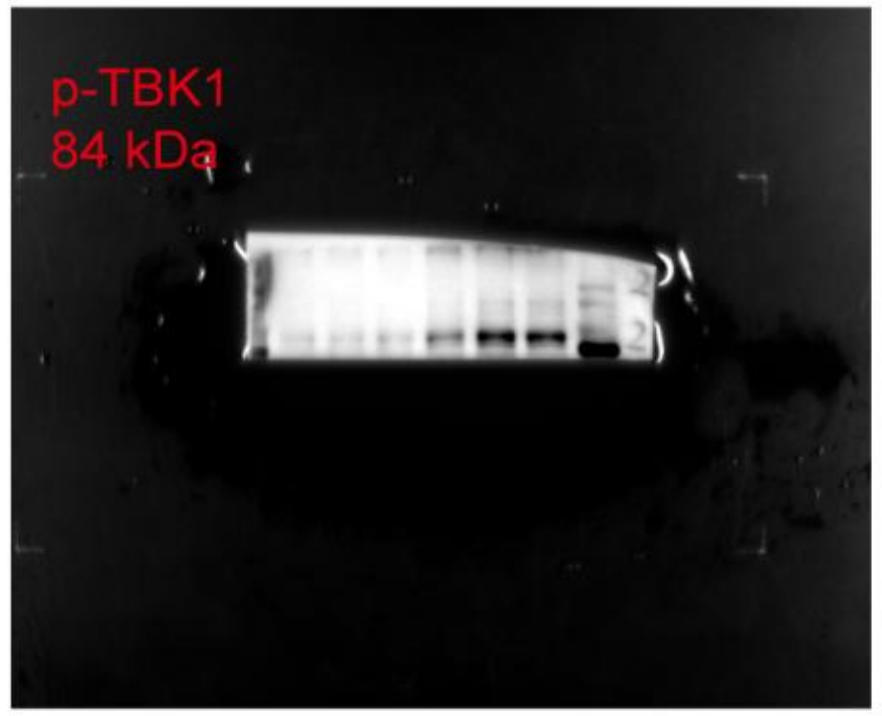

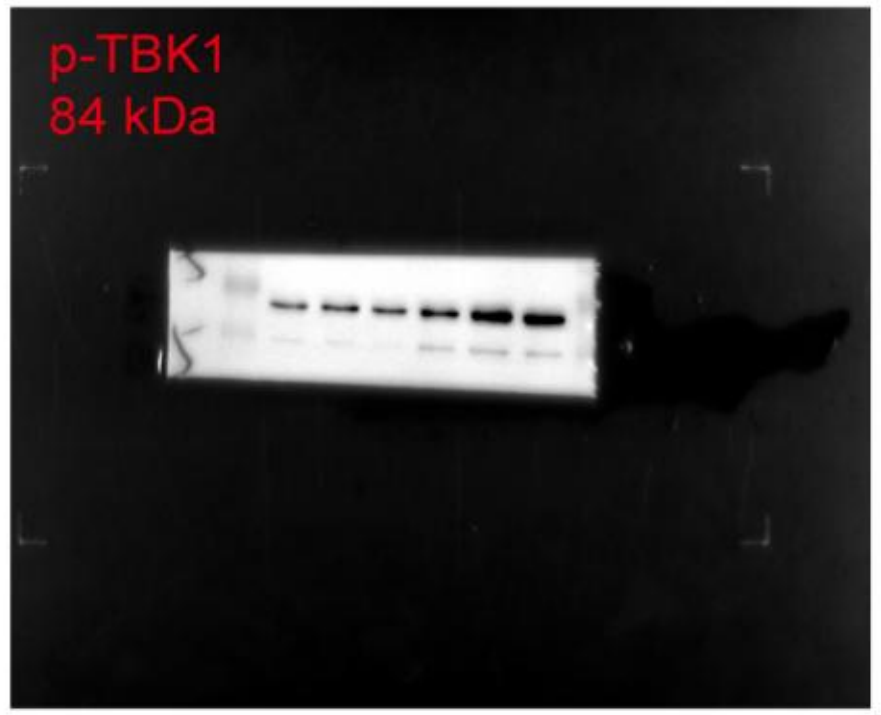

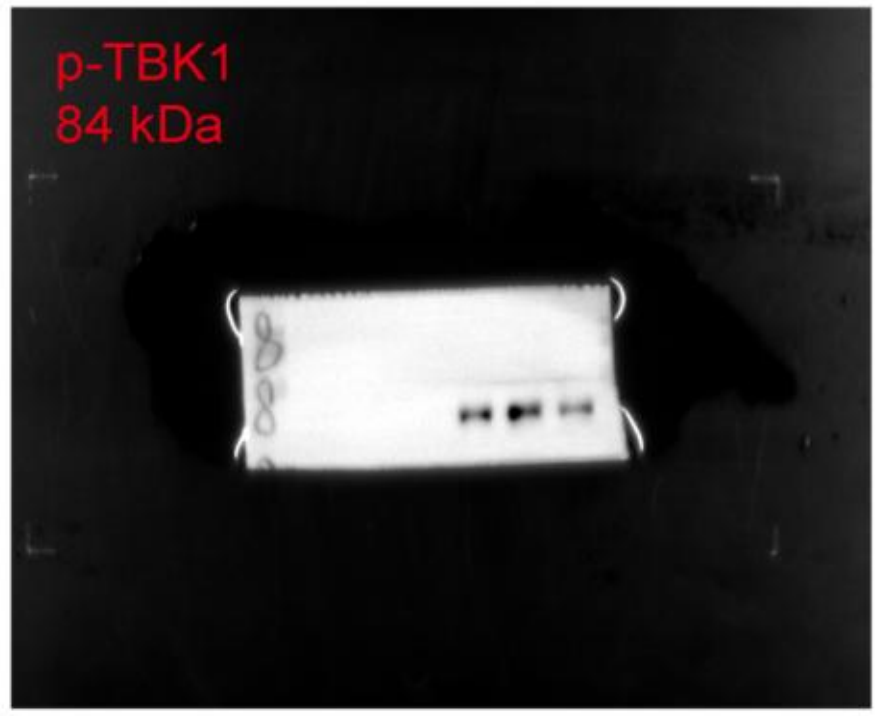

GAPDH (~37 kDa)

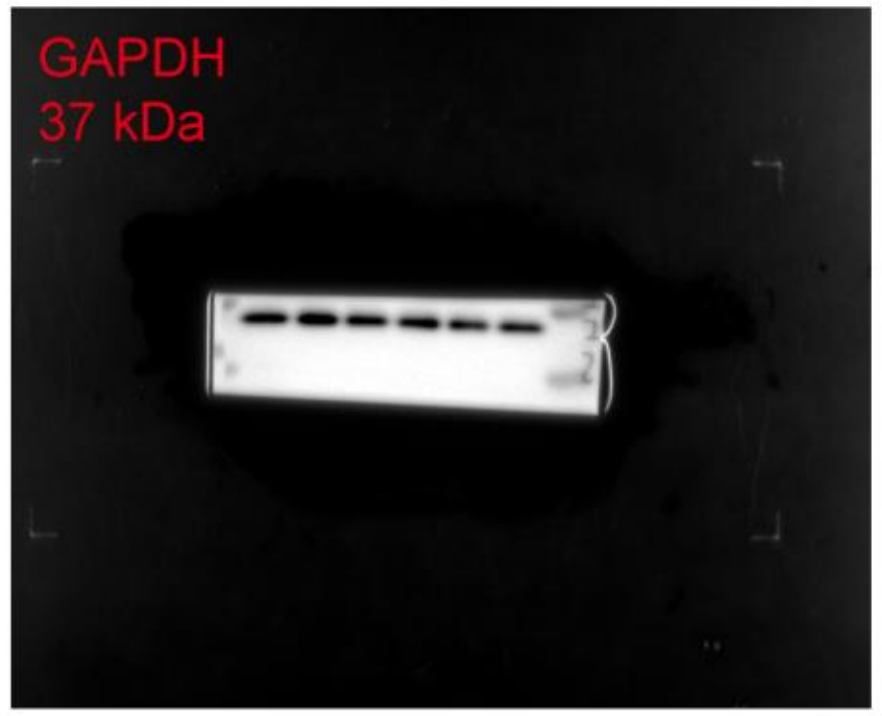

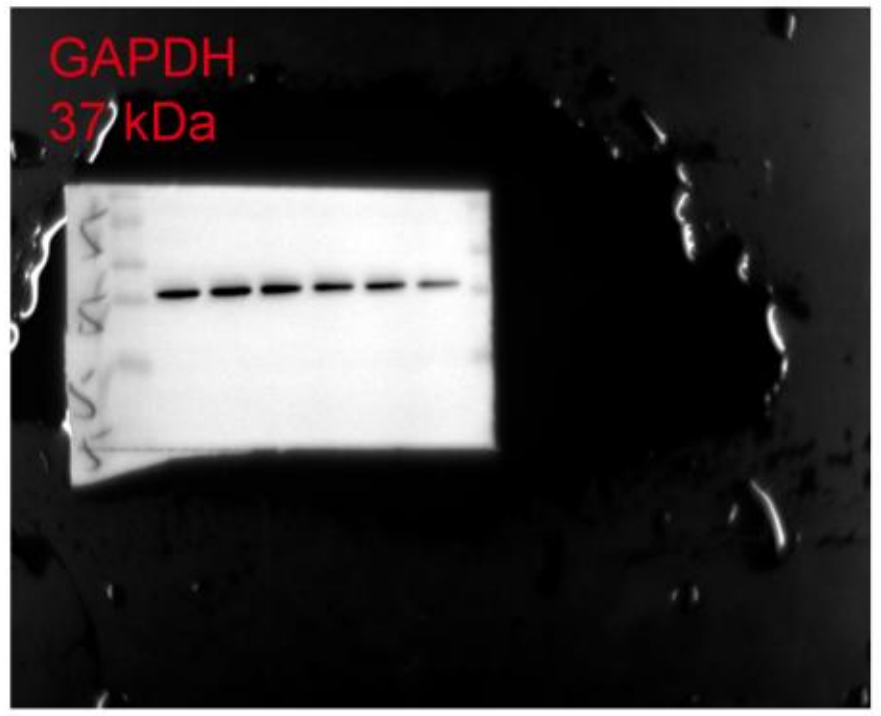

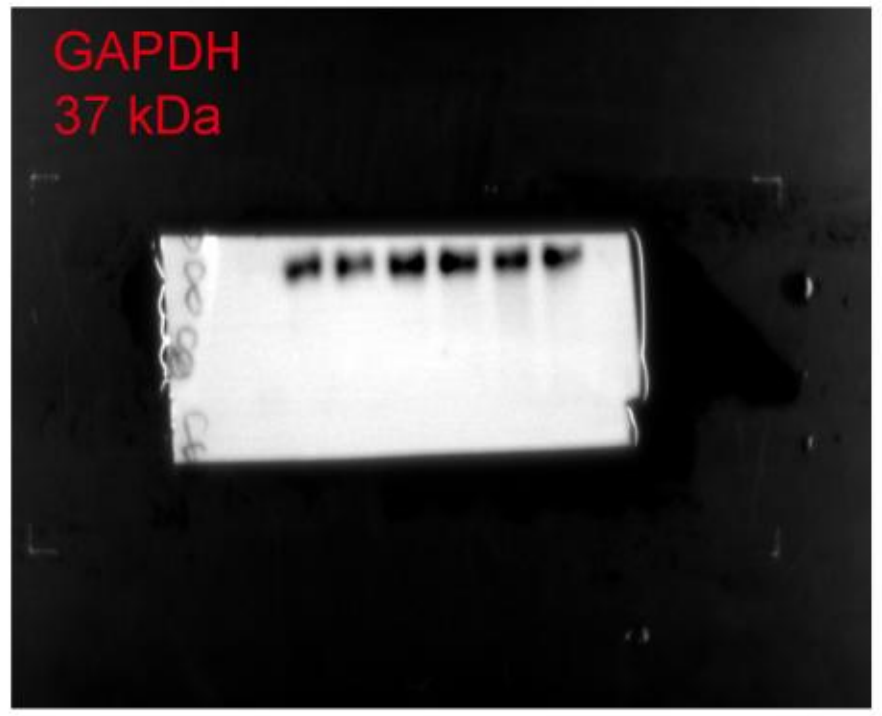

Anti-TIM-3-Original Archived Western Blot Images from Three Independent Biological Replicates

	Replicate 1<br>Representative blot shown in Fig. 7A	Replicate 2<br>Independent biological replicate	Replicate 3<br>Independent biological replicate

Anti-TIM-3 (~55 kDa)

Na^+/K^+ATP ase (~112 kDa)

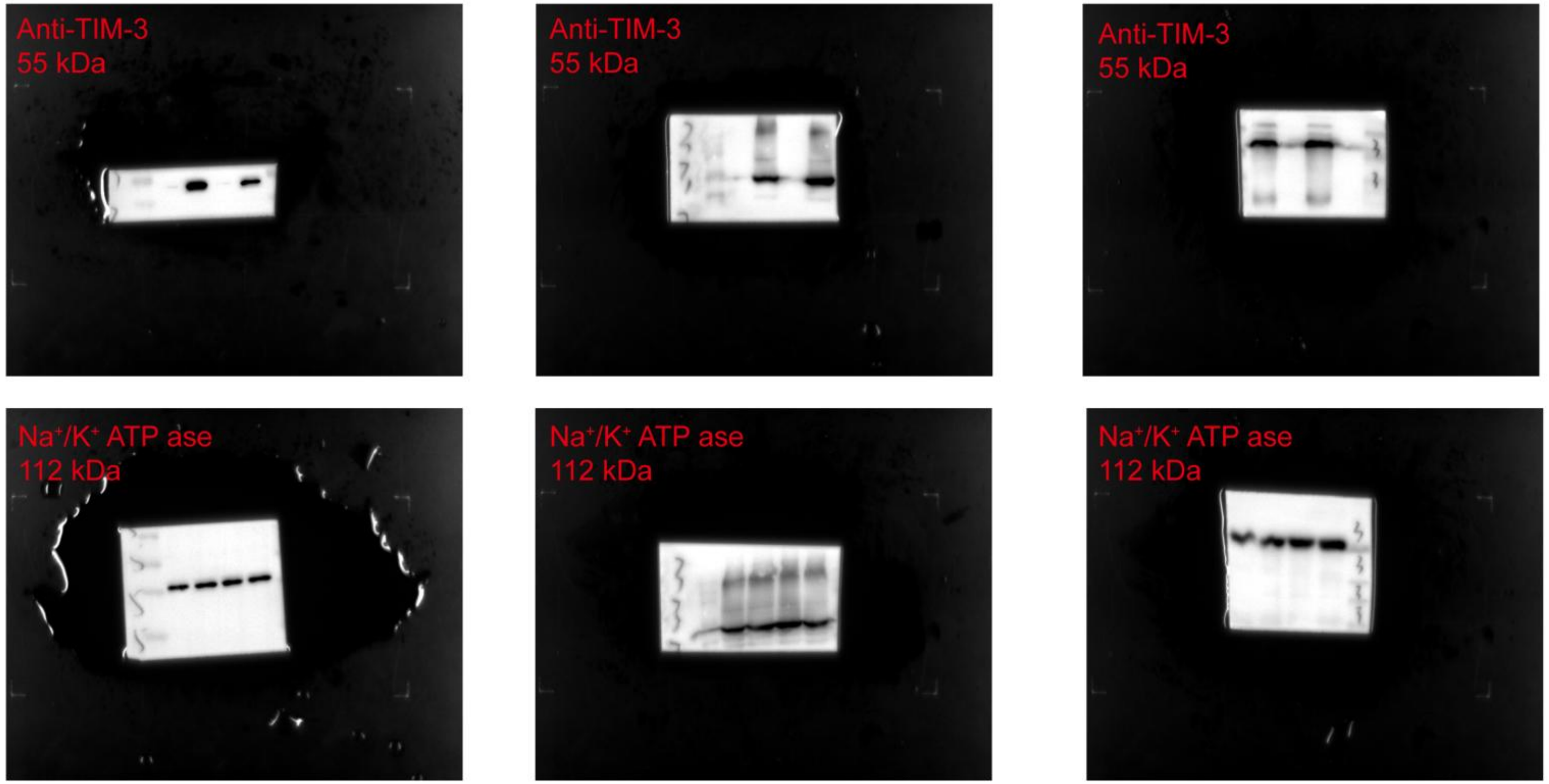

CD86-Original Archived Western Blot Images from Three Independent Biological Replicates

	Replicate 1 Representative blot shown in Fig. 7A	Replicate 2 Independent biological replicate	Replicate 3 Independent biological replicate

CD86 (~70 kDa)

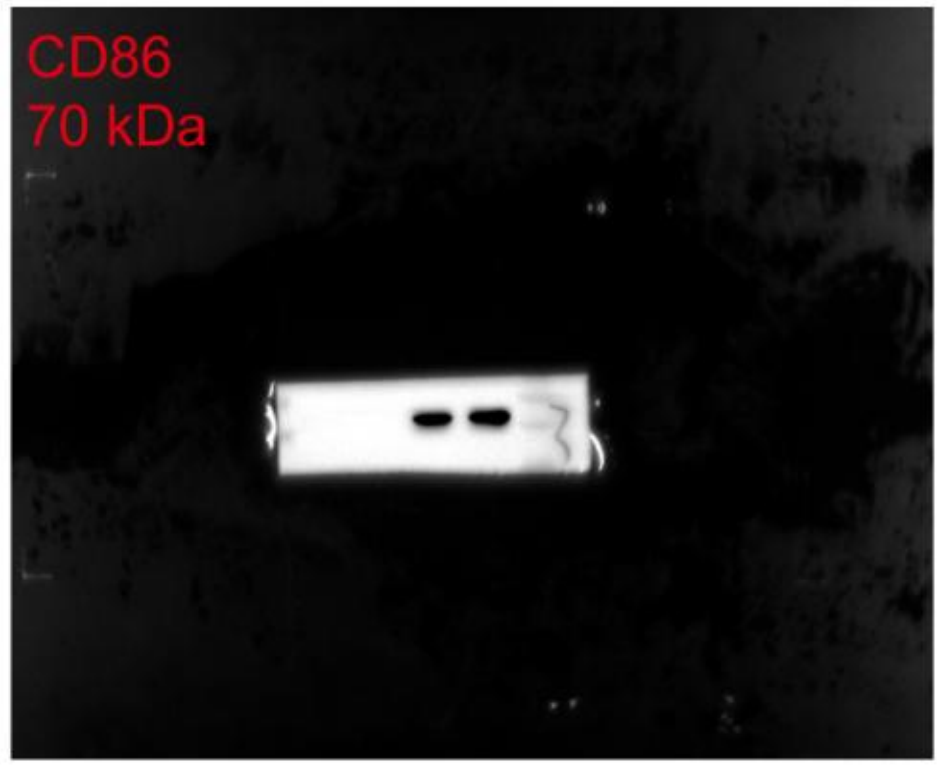

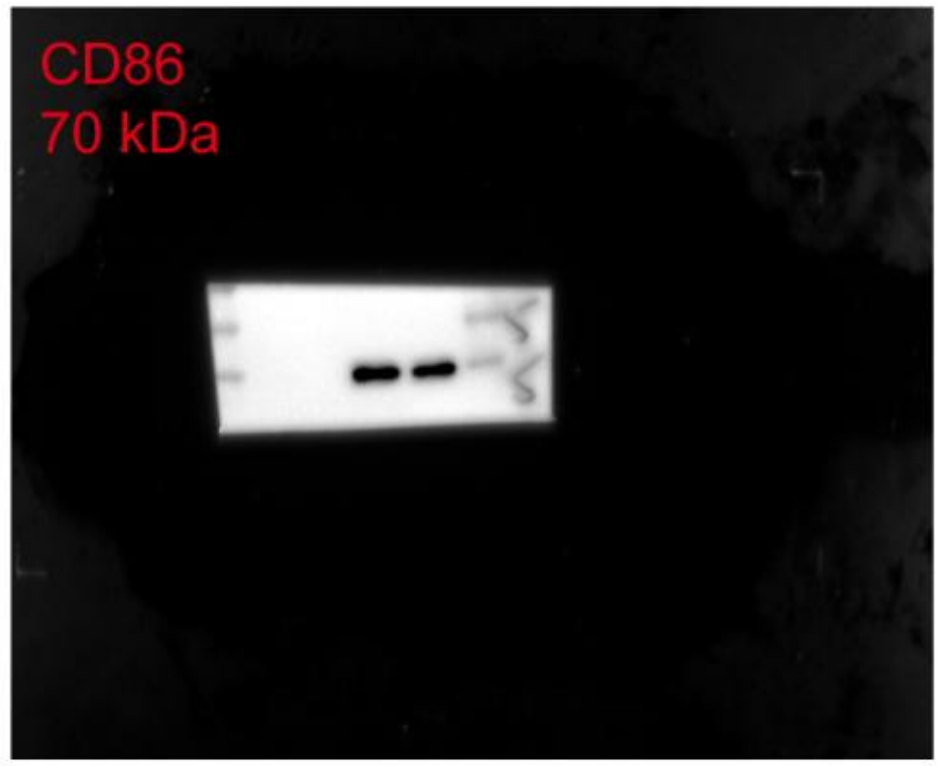

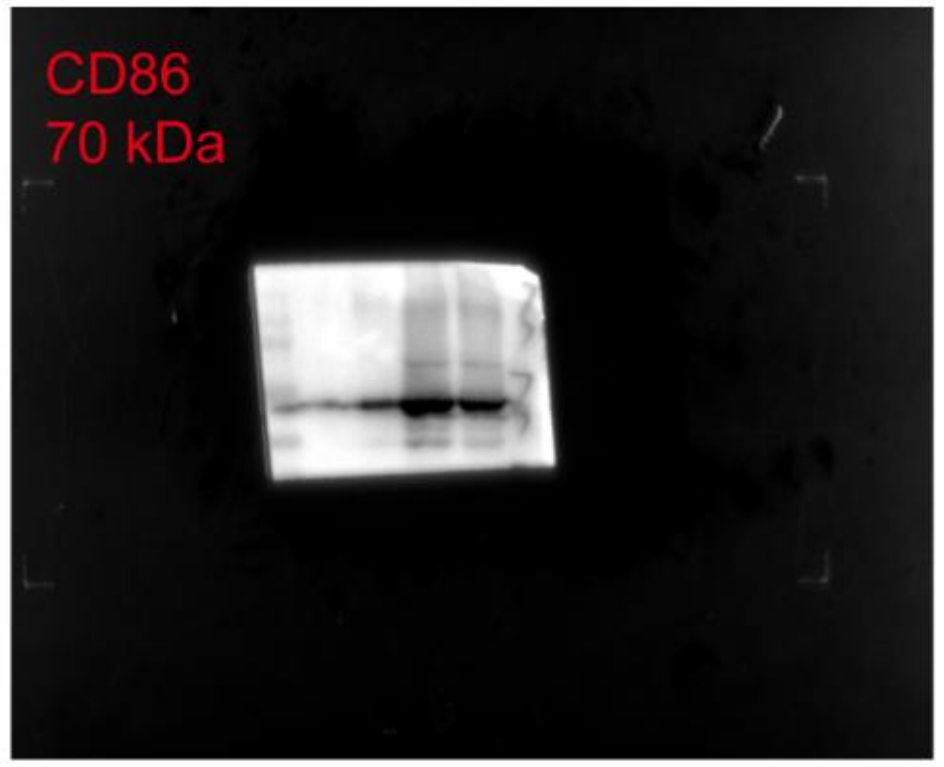

Na^+/K^+ATP ase (~112 kDa)

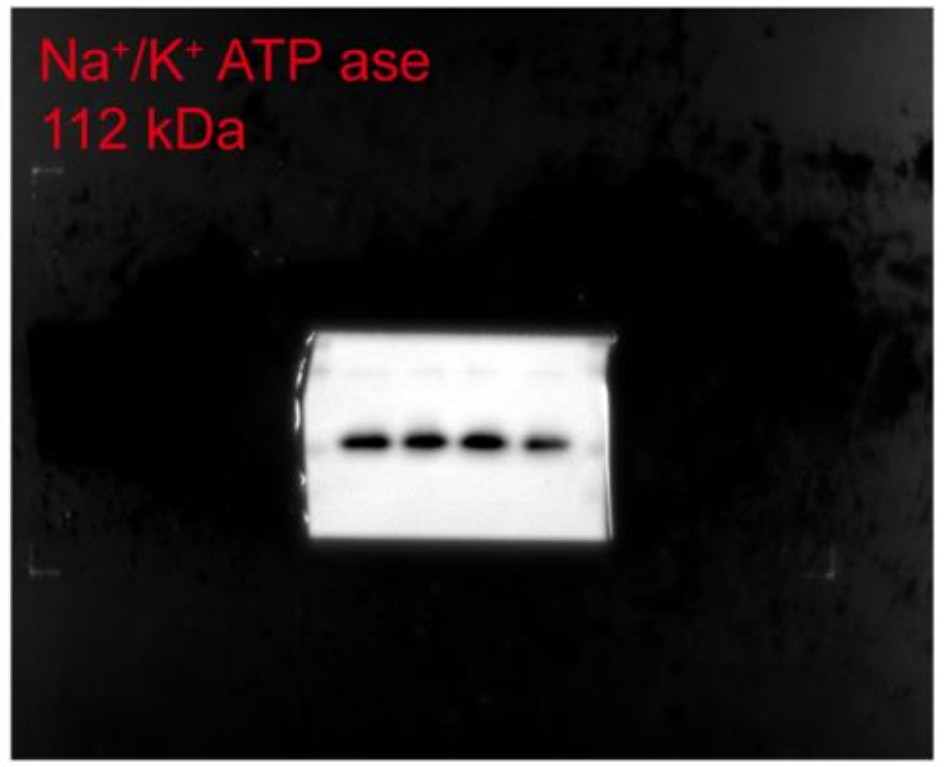

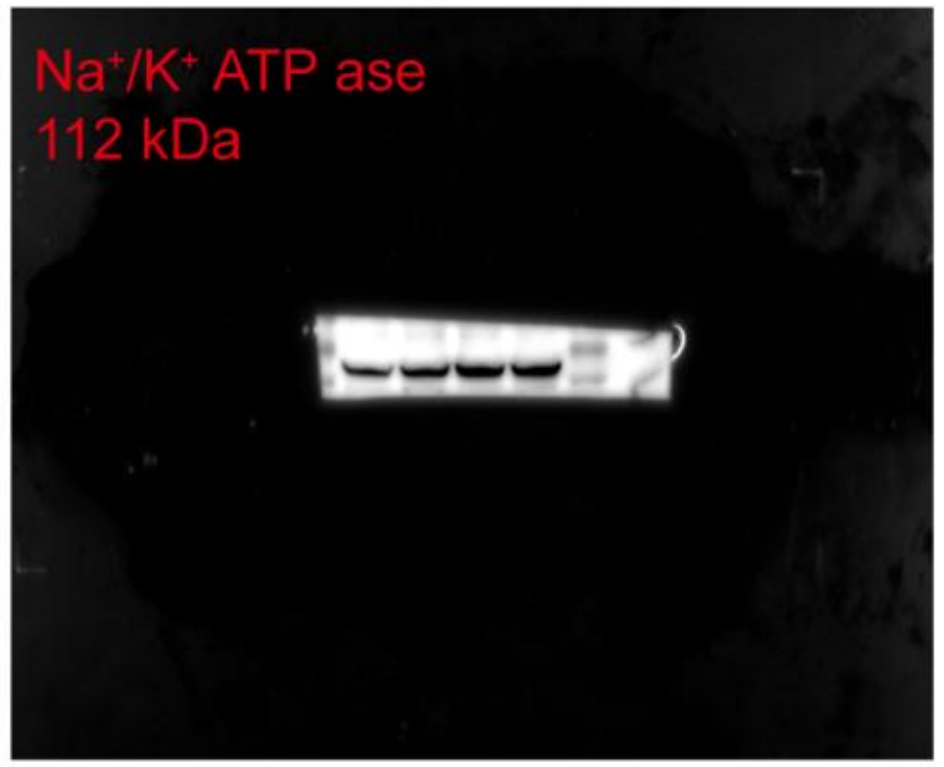

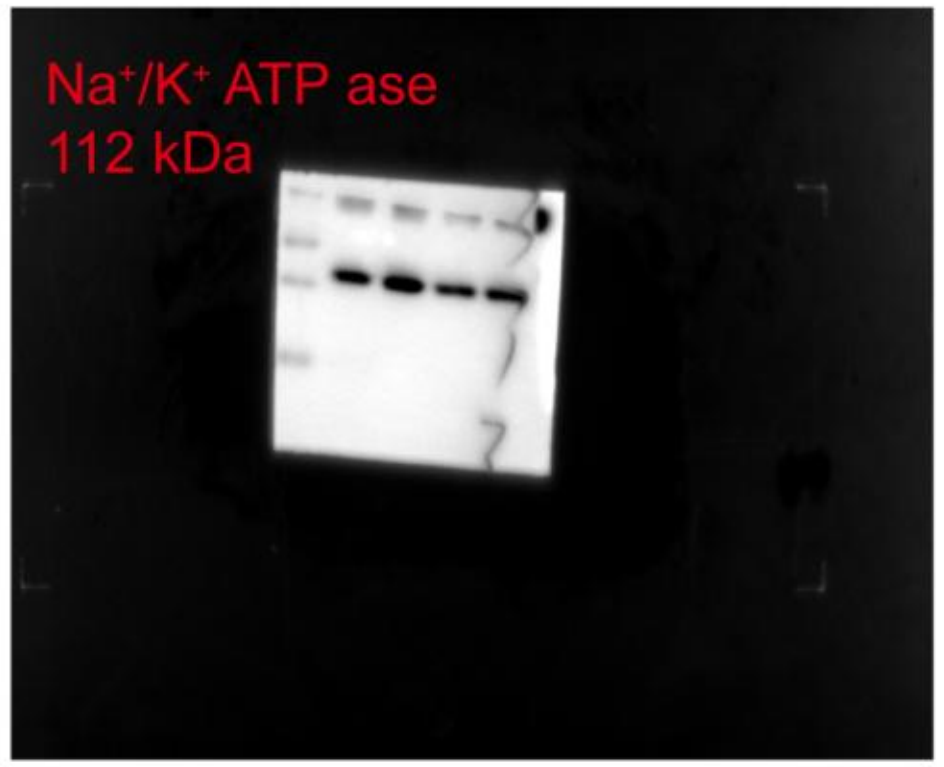

HIF-1α– Original Archived Western Blot Images from Three Independent Biological Replicates

	Replicate 1<br>Representative blot shown in Fig. S10A	Replicate 2<br>Independent biological replicate	Replicate 3<br>Independent biological replicate

HIF-1α (~93 kDa)

Tubulin (~56 kDa)

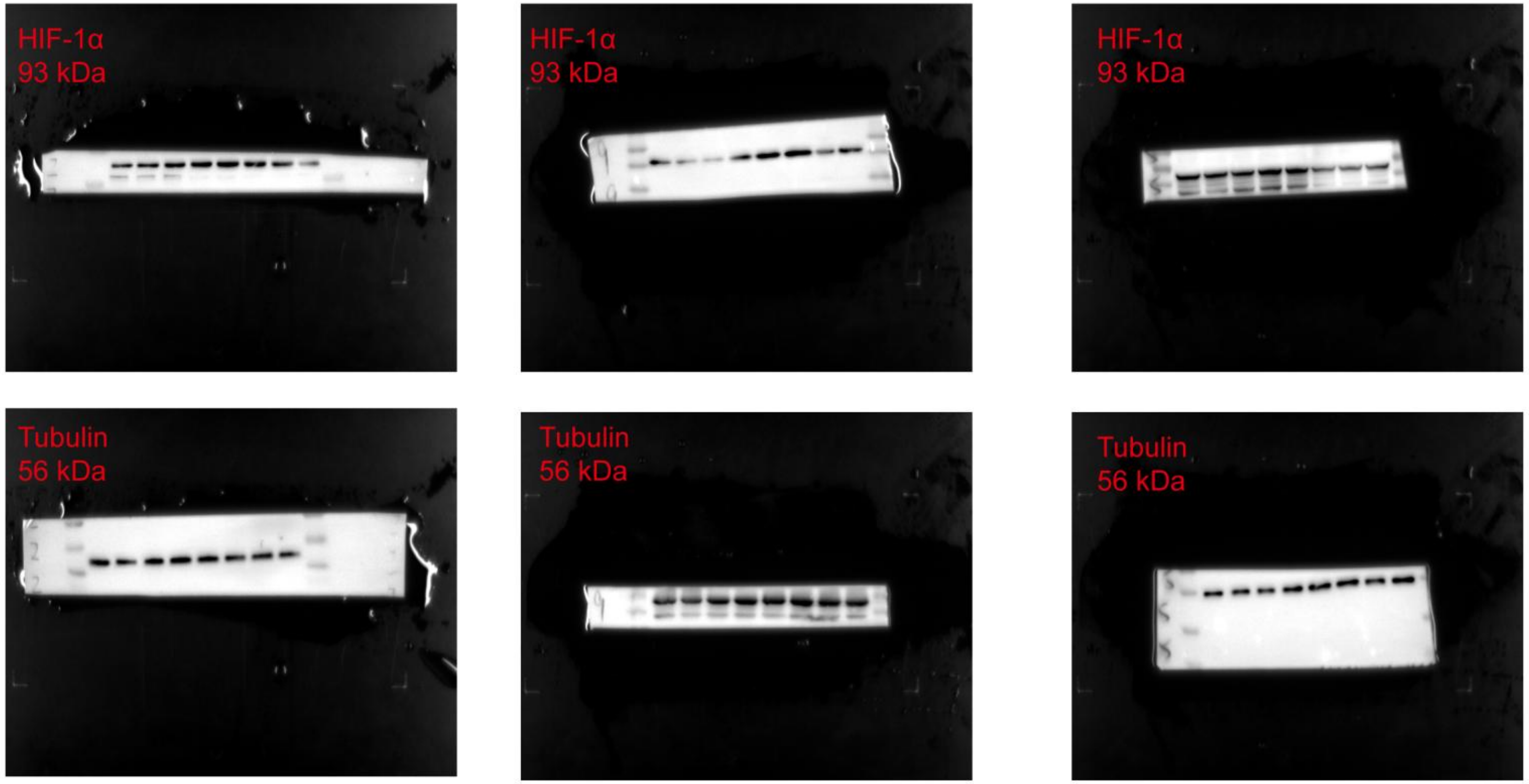

TFRC – Original Archived Western Blot Images from Three Independent Biological Replicates

	Replicate 1 Representative blot shown in Fig. S14A	Replicate 2 Independent biological replicate	Replicate 3 Independent biological replicate

TFRC (~95 kDa)

Tubulin (~56 kDa)

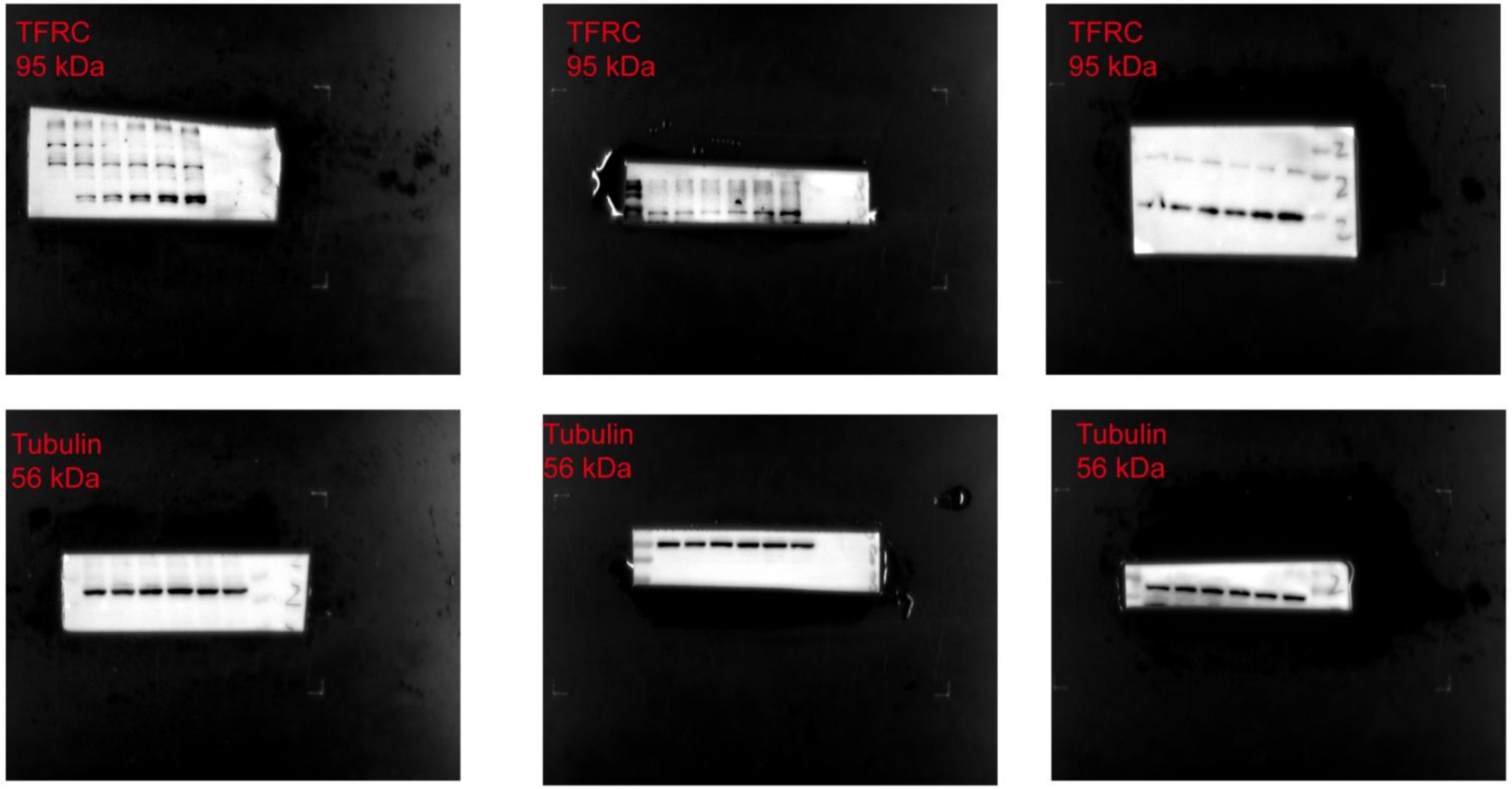

FTH1 – Original Archived Western Blot Images from Three Independent Biological Replicates

Replicate 1
Representative blot shown in Fig. S14A

Replicate 2
Independent biological replicate

Replicate 3
Independent biological replicate

FTH1 (~21 kDa)

Tubulin (~56 kDa)

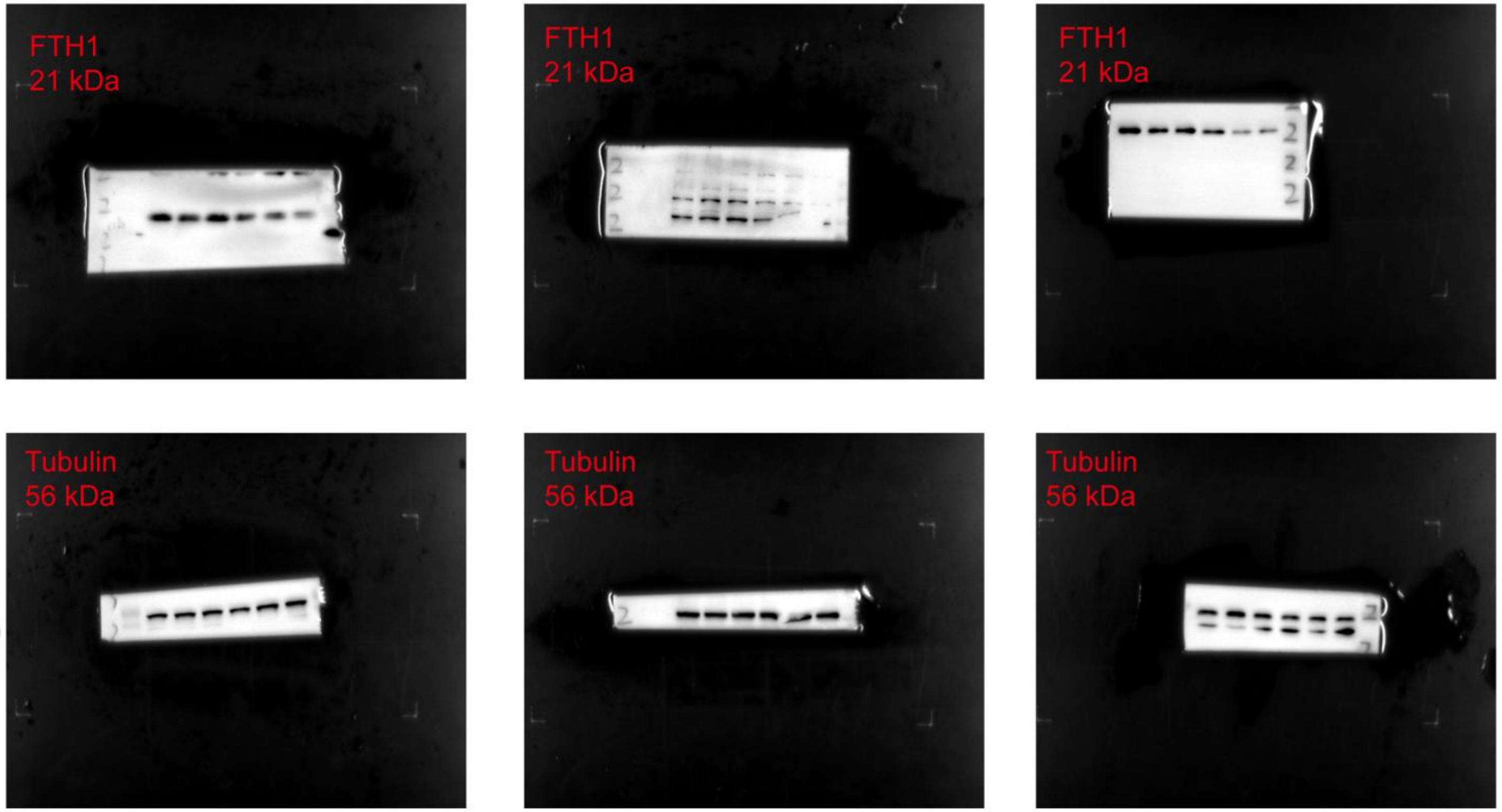

CRT – Original Archived Western Blot Images from Three Independent Biological Replicates

	Replicate 1<br>Representative blot shown in Fig. S17A	Replicate 2<br>Independent biological replicate	Replicate 3<br>Independent biological replicate

CRT (~60 kDa)

GAPDH (~37 kDa)

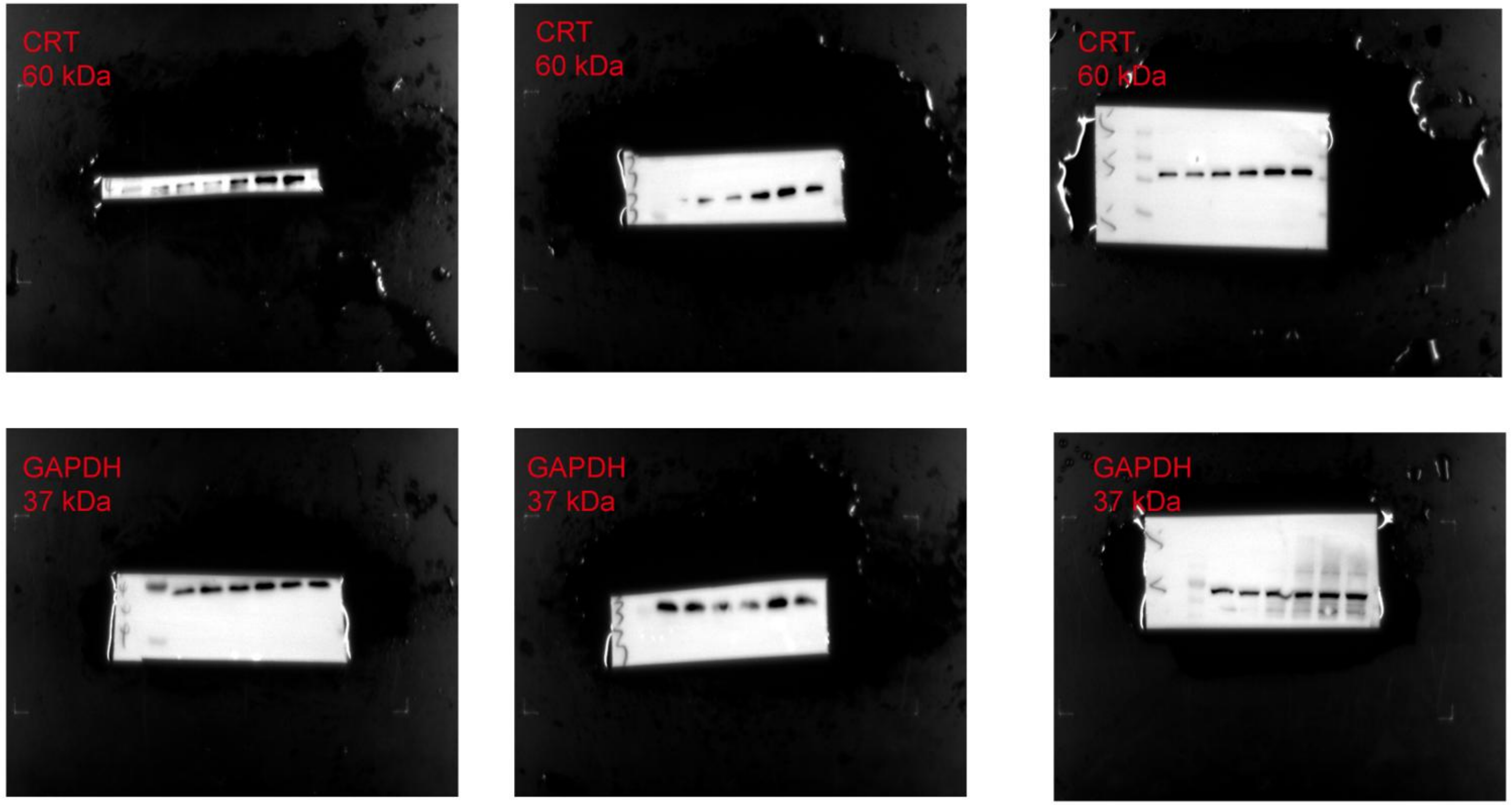

HMGB1 – Original Archived Western Blot Images from Three Independent Biological Replicates

Replicate 1
Representative blot shown in Fig. S17B

Replicate 2
Independent biological replicate

Replicate 3
Independent biological replicate

HMGB1 (~25 kDa)

Tubulin (~56 kDa)

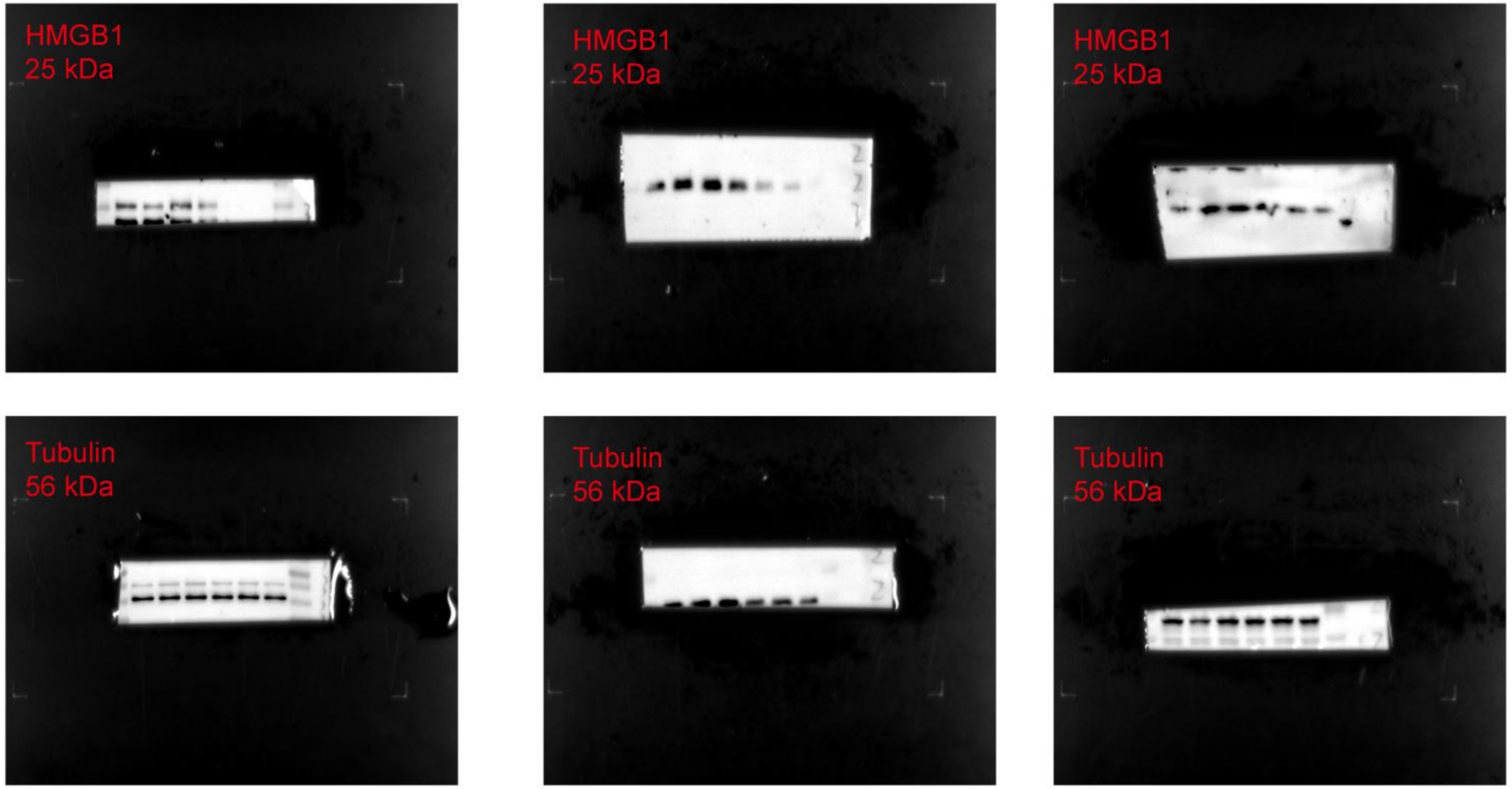

HIF-1α (Fig. S20A) – Original Archived Western Blot Images from Three Independent Biological Replicates

Replicate 1
Representative blot
shown in Fig. S20A

Replicate 2
Independent
biological replicate

Replicate 3
Independent
biological replicate

HIF-1α (~93 kDa)

GAPDH (~37 kDa)

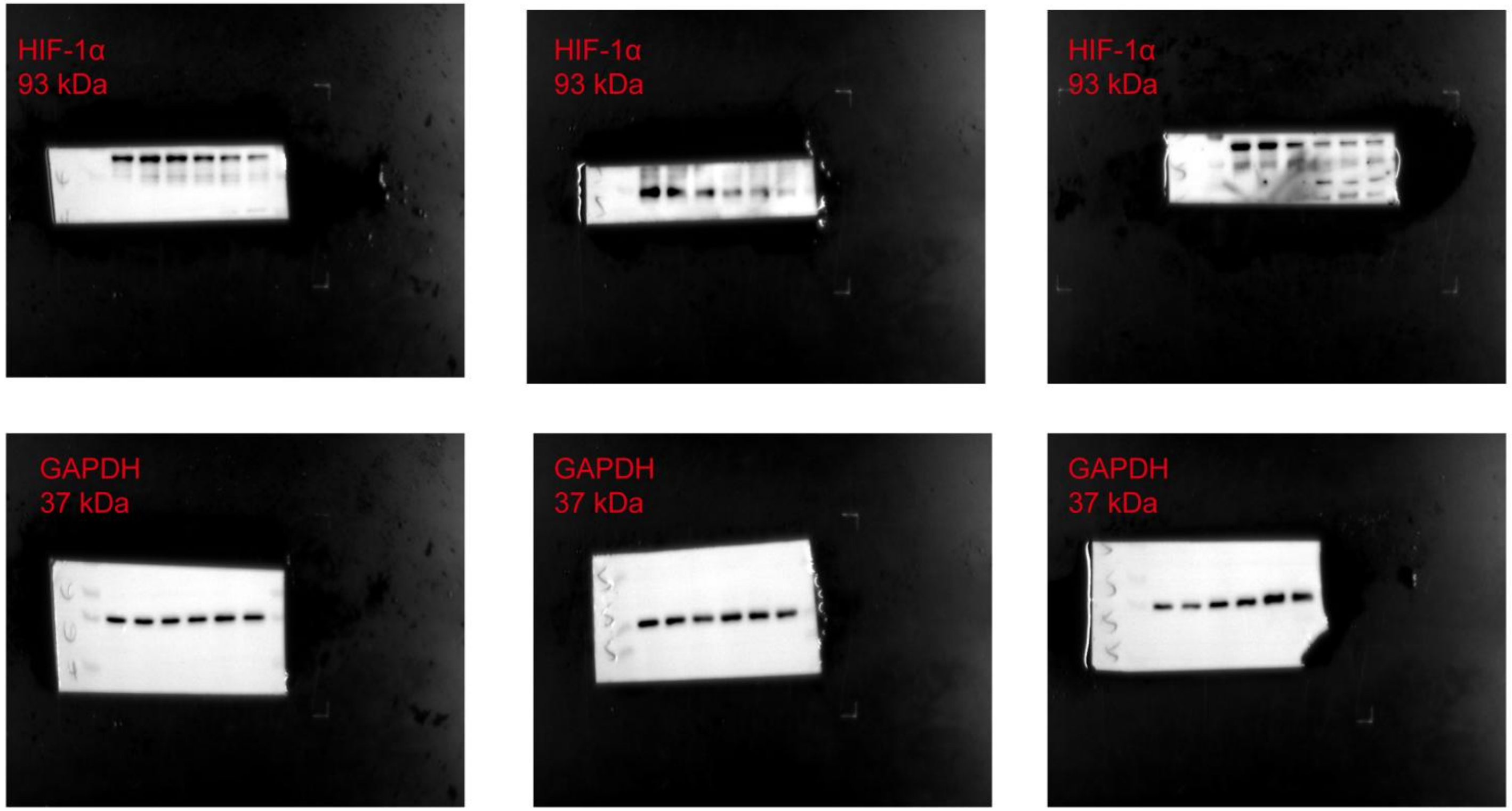

Supplement: Supplementary file 1 [file ADVS-9999-e78187-s002.pdf]
