# Supplementary material for: Ultrasound‐Activated Ce(IV)‐Integrated Biomimetic Nanoplatform for Oxygenation‐Reinforced Gas‐Sonodynamic Ferroptosis and cGAS–STING‐Driven Antitumor Immunity
Source: Adv Sci (Weinh). 2026 Oct 31:e78187. Online ahead of print. doi: 10.1002/advs.78187 (PMC13723706; doi:10.1002/advs.78187)
Supplement: Supplementary file 2 [file ADVS-9999-e78187-s001.docx]

**Ultrasound-Activated Ce(IV)-Integrated Biomimetic Nanoplatform for Oxygenation-Reinforced Gas-Sonodynamic Ferroptosis and cGAS–STING-Driven Antitumor Immunity**

*Mengli Ma, Yingying Wu,* *Yunjie Wang,* *Yang Li*, Yijia Zhang, Zhibo Zhang, Jinzhi Peng, Xiangyu Wu, Yi Luo, Weijing Zhang, Xin Ye, Weisong Lai, Shengyu Wang, Dan Du, Wengang Li*, Nan Zheng*, Fanghong Luo*, and Ting Wu**

*M. Ma, Y. Wu, Y. Wang, Y. Zhang, Z. Zhang, J. Peng, W. Zhang, X. Ye, W. Lai, S. Wang, D. Du, W. Li, F. Luo, T. Wu*

Cancer Research Center, School of Medicine, Xiamen University, Xiamen 361000, P. R. China

E-mail: lwg11861@163.com; luofanghong@xmu.edu.cn; [wuting78@189.cn](mailto:wuting78@189.cn)

*T. Wu*

Department of Basic Medicine, School of Medicine, Xiamen University, Xiang’an South Road, Xiang’an District, Xiamen 361000, P. R. China

*W. Li*

Department of Hepatobiliary Surgery, Xiang’an Hospital of Xiamen University, School of Medicine, Xiamen University, Xiamen, P. R. China

*Y. Li*

State Key Laboratory of Structural Chemistry & CAS Key Laboratory of Design and Assembly of Functional Nanostructures, Fujian Institute of Research on the Structure of Matter, Chinese Academy of Sciences, Fuzhou 350002, P. R. China

E-mail: [li.yang@fjirsm.ac.cn](mailto:li.yang@fjirsm.ac.cn)

*X. Wu, Y. Luo*

School of Basic Medicine, Fujian Medical University, Fuzhou, Fujian, P. R. China.

*N. Zheng*

School of Chemical Engineering, Dalian University of Technology, Dalian, Liaoning, China.

E-mail: [nzheng@dlut.edu.cn](mailto:nzheng@dlut.edu.cn)

M. Ma, Y. Wu, Y. Wang, and Y. Li contributed equally to this work.

**Keywords:** biomimetic nanoplatforms, sonodynamic therapy, carbon monoxide therapy, ferroptosis, cGAS–STING, antitumor immunity

Figure S1. SDS-PAGE analysis of membrane protein profiles. SDS-PAGE profiles of Ce-P4CO@M, TcEM, and TcEM@Ce-P4CO@M.

Figure S2. High-resolution XPS spectra of TcEM@Ce-P4CO@M. (A–C) High-resolution XPS spectra of O 1s, S 2p, and N 1s, respectively.

Figure S3. Colloidal stability of TcEM@Ce-P4CO@M in different media. Changes in (A) hydrodynamic size, (B) zeta potential, and (C) polydispersity index (PDI) of TcEM@Ce-P4CO@M during incubation in PBS, DMEM, and DMEM containing 10% FBS. Data are presented as mean ± SD (n = 3 independent measurements).

Figure S4. In vitro release profiles of MSA-2 from TcEM@Ce-P4CO@M. Cumulative release of MSA-2 from TcEM@Ce-P4CO@M at pH 7.4, 6.5, and 5.0 in the presence or absence of GSH. Data are presented as mean ± SD (n = 3 independent measurements).

Figure S5. Ultrasound-responsive spectral changes of P4CO-0P. (A–C) Time-dependent UV–vis absorption spectra of P4CO-0P under ultrasound irradiation at 0.5, 1.0, and 1.5 W cm^-2^, respectively.

Figure S6. Additional DPBF spectra during ultrasound irradiation. (A, B) Time-dependent DPBF absorption spectra under ultrasound irradiation at 0.5 and 1.0 W

cm^-2^, respectively.

Figure S7. Additional MB spectra during ultrasound irradiation. (A, B) Time-dependent MB absorption spectra under ultrasound irradiation at 0.5 and 1.0 W cm^-2^, respectively.

Figure S8. Schematic illustration of the probe reactions used for oxidative activity measurements. Reaction schemes of (A) DPBF, (B) MB, and (C) DTNB-based assays.

Figure S9. Ultrasound-induced thiol oxidation evaluated using a DTNB-based assay.

(A–C) Time-dependent absorption spectra under ultrasound irradiation at 0.5, 1.0, and 1.5 W cm^-2^, respectively. (D) Corresponding absorbance changes at 412 nm under different acoustic power densities.

Figure S10. Western blot analysis of HIF-1α expression under normoxic and CoCl_2_-induced hypoxia-mimetic conditions. (A) Representative Western blot images and (B) corresponding densitometric analysis of HIF-1α expression. Tubulin was used as the loading control. Groups: G1, PBS; G2, US; G3, P4CO@M + US; G4, Ce-P4CO@M + US; G5, PBS + CoCl_2_; G6, US + CoCl_2_; G7, P4CO@M + US + CoCl_2_; G8, Ce-P4CO@M + US + CoCl_2_. HIF-1α levels were normalized to Tubulin. Data are presented as mean ± SD (n = 3 independent experiments). Statistical significance was determined by two-way ANOVA followed by Tukey’s multiple-comparisons test. Brackets indicate the specific group comparisons shown in the figure. ****p* < 0.001.

Figure S11. Comparative cellular association and uptake of Ce-P4CO@M and TcEM@Ce-P4CO@M by 4T1 tumor cells and CD8^+^ T cells. (A) Representative flow-cytometric profiles of nanoparticle association with 4T1 cells and CD8^+^ T cells after incubation at 4 °C for 30 min. (B) Quantification of DiD^+^ cells corresponding to (A). (C) Representative flow-cytometric profiles of nanoparticle uptake by 4T1 cells and CD8^+^ T cells after incubation at 37 °C for 2 h. (D) Quantification of DiD^+^ cells corresponding to (C). Groups: G1, 4T1 + Ce-P4CO@M; G2, 4T1 + TcEM@Ce-P4CO@M; G3, CD8^+^ T cells + Ce-P4CO@M; G4, CD8^+^ T cells + TcEM@Ce-P4CO@M. For each cell type and temperature condition, the DiD-positive gate was defined using the corresponding unstained control and applied consistently to the matched nanoparticle-treated groups. Data are presented as mean ± SD (n = 3 independent experiments). For each temperature condition, statistical significance was determined by two-way ANOVA followed by Tukey’s multiple-comparisons test. Horizontal lines indicate the specific group comparisons shown in the figure. ***p* < 0.01, ****p* < 0.001.

Figure S12. CO binding attenuates TcEM@Ce-P4CO@M-induced mitochondrial oxidative stress and lipid peroxidation without markedly affecting overall ROS generation. Representative flow-cytometric profiles and corresponding quantitative analyses of 4T1 cells subjected to different treatments. (A, B) Intracellular CO-associated fluorescence detected using a CO-sensitive fluorescent probe and corresponding relative mean fluorescence intensity (MFI). (C, D) Intracellular ROS levels assessed using DCFH-DA and corresponding relative MFI. (E, F) Mitochondrial ROS levels evaluated using MitoSOX Red and corresponding relative MFI. (G, H) Lipid peroxidation assessed using C11-BODIPY 581/591 and corresponding relative oxidized C11-BODIPY MFI. Hemoglobin (Hb) was used as a CO-scavenging agent to evaluate the contribution of CO to mitochondrial oxidative stress and ferroptotic lipid peroxidation. G1, PBS; G2, Hb + US; G3, TcEM@Ce-P4CO@M + US; G4, Hb + TcEM@Ce-P4CO@M + US. Quantitative fluorescence intensities were normalized to the G1 group. Data are presented as mean ± SD (n = 3 independent experiments). Statistical significance was determined by one-way ANOVA followed by Tukey’s multiple-comparisons test. ***p* < 0.01, ****p* < 0.001, *****p* < 0.0001; *ns*, not significant.

Figure S13. Densitometric analysis of ferroptosis-associated antioxidant proteins after ultrasound-activated treatment. (A) Quantification of GPX4 protein expression normalized to Tubulin. (B) Quantification of SLC7A11 protein expression normalized to GAPDH. Groups: G1, PBS; G2, US; G3, MSA-2; G4, P4CO + US; G5, Ce-P4CO@M + US; G6, TcEM@Ce-P4CO@M + US. Data are presented as mean ± SD from three independent experiments. Statistical significance was determined by one-way ANOVA followed by Tukey’s multiple-comparisons test. ***p* < 0.01, ****p* < 0.001, *****p* < 0.0001.

Figure S14. Endogenous iron-homeostasis remodeling and intracellular iron accumulation after ultrasound-activated treatment. (A) Representative Western blot images of FTH1 and TFRC expression in 4T1 cells after the indicated treatments. Tubulin was used as the loading control. (B, C) Densitometric quantification of FTH1/Tubulin and TFRC/Tubulin, respectively, corresponding to the Western blots shown in (A). (D–F) Quantification of intracellular Fe^2+^, Fe^3+^, and total Fe concentrations, respectively, in 4T1 cells after the indicated treatments. Groups: G1, PBS; G2, US; G3, MSA-2; G4, P4CO + US; G5, Ce-P4CO@M + US; G6, TcEM@Ce-P4CO@M + US. Data are presented as mean ± SD (n = 3 independent experiments). Statistical significance was determined by one-way ANOVA followed by Tukey’s multiple-comparisons test. Horizontal lines indicate the specific group comparisons shown in the figure. **p* < 0.05, ***p* < 0.01, ****p* < 0.001, *****p* < 0.0001.

Figure S15. GPX4 expression under normoxic and CoCl₂-induced hypoxia-mimetic conditions after ultrasound-activated treatment. (A) Representative immunofluorescence images of GPX4 expression in 4T1 cells under normoxic or CoCl₂-induced hypoxia-mimetic conditions following the indicated treatments. GPX4 is shown in green and nuclei are counterstained with DAPI (blue). (B) Quantification of relative GPX4 fluorescence intensity corresponding to the images shown in (A). Under each oxygen condition, the treatment groups were PBS, US, P4CO@M + US, and Ce-P4CO@M + US. Scale bar: 25 μm. Data are presented as mean ± SD (n = 3 independent experiments). Statistical significance was analyzed by two-way ANOVA followed by Tukey’s multiple-comparisons test. ***p* < 0.01.

Figure S16. Quantitative analysis of DNA-damage and cGAS–STING-associated signaling proteins. (A) Densitometric quantification of γ-H2AX normalized to Tubulin corresponding to the Western blot shown in Figure 6E. (B) Quantification of the p-STING/STING ratio corresponding to Figure 6F. (C–E) Quantitative analyses of p-TBK1/TBK1, p-p65/p65, and p-IRF3/IRF3, respectively, corresponding to the Western blots shown in Figure 6G. Groups: G1, PBS; G2, US; G3, MSA-2; G4, P4CO + US; G5, Ce-P4CO@M + US; G6, TcEM@Ce-P4CO@M + US. Data are presented as mean ± SD from three independent experiments. Statistical significance was determined by one-way ANOVA followed by Tukey’s multiple-comparisons test. **p* < 0.05, ***p* < 0.01, ****p* < 0.001, *****p* < 0.0001.

Figure S17. Western blot validation of ICD-associated CRT and HMGB1 changes.

(A, B) Representative Western blot analyses of cellular CRT and HMGB1 expression, respectively, in 4T1 cells after the indicated treatments. GAPDH and Tubulin were used as loading controls for CRT and HMGB1, respectively. (C, D) Corresponding densitometric quantification of CRT/GAPDH and HMGB1/Tubulin ratios, respectively. Groups: G1, PBS; G2, US; G3, MSA-2; G4, P4CO + US; G5, Ce-P4CO@M + US; G6, TcEM@Ce-P4CO@M + US. Data are presented as mean ± SD from three independent experiments. Statistical significance was determined by one-way ANOVA followed by Tukey’s multiple-comparisons test. **p* < 0.05, ***p* < 0.01, ****p* < 0.001, *****p* < 0.0001.

Figure S18. Comet assay analysis of DNA fragmentation after ultrasound-activated nanoplatform treatment. Representative comet assay fluorescence images of 4T1 cells after the indicated treatments. Increased comet-tail formation was observed in the ultrasound-activated P4CO-containing groups, with the most pronounced DNA fragmentation in the TcEM@Ce-P4CO@M + US group. Groups: G1, PBS; G2, US; G3, MSA-2; G4, P4CO + US; G5, Ce-P4CO@M + US; G6, TcEM@Ce-P4CO@M + US. Scale bar: 10 μm.

Figure S19. Densitometric analysis of anti-TIM-3 and CD86 expression in engineered tumor-derived membranes. (A) Quantification of anti-TIM-3 protein expression normalized to Na^+^/K^+^-ATPase corresponding to the Western blot shown in Figure 7A. (B) Quantification of CD86 protein expression normalized to Na^+^/K^+^-ATPase corresponding to Figure 7A. Groups: CM, anti-TIM-3-CM, CD86-CM, and dual-engineered TcEM. Data are presented as mean ± SD (n = 3 independent experiments). Statistical significance was determined by one-way ANOVA followed by Tukey’s multiple-comparisons test. ns, not significant; ****p* < 0.001, *****p* < 0.0001.

Figure S20. HIF-1α protein expression in tumor tissues after different treatments.

(A) Representative Western blot analysis of HIF-1α expression in tumor tissues collected from the indicated treatment groups, with GAPDH used as the loading control. (B) Densitometric quantification of HIF-1α expression normalized to GAPDH. Groups: G1, PBS; G2, US; G3, MSA-2; G4, Ce-P4CO + US; G5, Ce-P4CO@M + US; G6, TcEM@Ce-P4CO@M + US. Reduced HIF-1α expression in the Ce-containing treatment groups is consistent with attenuation of the intratumoral hypoxia-associated response. Data are presented as mean ± SD (n = 3 independent experiments). Statistical significance was determined by one-way ANOVA followed by Tukey’s multiple-comparisons test. ***p* < 0.01, ****p* < 0.001.

Figure S21. TcEM@Ce-P4CO@M + US reduces PD-1^+^TIM-3^+^ exhaustion-associated CD8^+^T cells in primary and distant tumors. (A) Representative flow-cytometric plots of PD-1 and TIM-3 expression within tumor-infiltrating CD8^+^ T cells in primary tumors. (B) Quantification of PD-1^+^TIM-3^+^ cells within the CD8^+^ T-cell population corresponding to (A). (C) Representative flow-cytometric plots of PD-1 and TIM-3 expression within CD8^+^T cells in distant tumors. (D) Quantification of PD-1^+^TIM-3^+^ CD8^+^ T cells corresponding to (C). Groups: G1, PBS; G2, US; G3, MSA-2; G4, Ce-P4CO + US; G5, Ce-P4CO@M + US; G6, TcEM@Ce-P4CO@M + US. Data are presented as mean ± SD (n = 5 mice per group). Statistical significance was determined by one-way ANOVA followed by Tukey’s multiple-comparisons test. **p* < 0.05, ***p* < 0.01, *****p* < 0.0001.

Figure S22. Representative flow-cytometry gating strategies for tumor-infiltrating immune-cell subsets and tumor-draining lymph node dendritic cells.

For T-cell analysis, debris and doublets were first excluded, followed by gating of CD45^+^ immune cells and CD3^+^ T cells. CD4^+^ and CD8^+^ T-cell subsets were subsequently identified within the CD3⁺ population, and PD-1^+^TIM-3^+^ exhaustion-associated cells were further quantified within the CD8^+^ T-cell population. For regulatory T-cell analysis, CD45^+^CD3^+^CD4^+^ cells were sequentially gated, and Tregs were defined as CD25^+^Foxp3^+^ cells within the CD4^+^ T-cell population. For tumor-associated macrophage analysis, CD45^+^ cells were gated after exclusion of debris and doublets, followed by identification of CD11b^+^F4/80^+^ TAMs. CD86^+^ and CD206^+^ populations within the TAM compartment were operationally defined as M1-like and M2-like macrophages, respectively. For dendritic-cell analysis in tumor-draining lymph nodes (TDLNs), CD45^+^ immune cells were gated after exclusion of debris and doublets, followed by identification of CD11c^+^ DCs; mature DCs were defined as CD80⁺CD86⁺ cells within the CD11c^+^ population.

Figure S23. Gene set enrichment analysis of immune-associated transcriptional programs following TcEM@Ce-P4CO@M + US treatment. (A) Gene set enrichment analysis (GSEA) of the antigen-binding gene set in PBS- and TcEM@Ce-P4CO@M + US-treated tumors. (B) GSEA of the phagocytosis/recognition-related gene set. The observed enrichment of these gene sets further supports enhanced transcriptional programs associated with antigen recognition and innate immune processing following TcEM@Ce-P4CO@M + US treatment.
